# Expanding the actinomycetes landscape for phosphonate natural products through genome mining

**DOI:** 10.1039/d5cb00254k

**Published:** 2025-12-16

**Authors:** Alina Zimmermann, Shu-Ning Xia, Julia Moschny, Juan Pablo Gomez-Escribano, Judith Boldt, Ulrich Nübel, Imen Nouioui, Janina Krause, Mattis Kreins Irle, William W. Metcalf, Chambers C. Hughes, Yvonne Mast

**Affiliations:** a Leibniz Institute DSMZ -German Collection of Microorganisms and Cell Cultures Inhoffenstraße 7B 38124 Braunschweig Germany yvonne.mast@dsmz.de; b German Center for Infection Research (DZIF), Partner Site Tübingen 72076 Tübingen Germany; c Department of Microbial Bioactive Compounds, Interfaculty Institute of Microbiology and Infection Medicine (IMIT), University of Tübingen Auf der Morgenstelle 28 72076 Tübingen Germany; d German Center for Infection Research (DZIF), Partner Site Hannover-Braunschweig 38124 Braunschweig Germany; e Braunschweig Integrated Centre of Systems Biology (BRICS) Rebenring 56 38106 Braunschweig Germany; f Technische Universität Braunschweig, Institut für Mikrobiologie Spielmannstr. 7 38106 Braunschweig Germany; g Carl R. Woese Institute for Genomic Biology 1206 West Gregory Drive Urbana IL 61801 USA; h Cluster of Excellence EXC 2124: Controlling Microbes to Fight Infection, University of Tübingen 72076 Tübingen Germany

## Abstract

Phosphonate natural products (P-NPs) represent a unique and underexplored class of bioactive compounds with significant pharmaceutical and biotechnological potential. Many novel P-NPs with promising bioactivities were identified in recent years by genome mining of actinomycetes. The DSMZ strain collection harbors more than 6000 actinobacterial strains including an increasing number of genome sequenced strains. In this study, 940 genome-sequenced actinomycetes from the DSMZ and University of Tübingen strain collections were screened for the presence of phosphonate biosynthetic gene clusters (P-BGCs) by searching for the conserved *pepM* gene. This effort led to the identification of 54 potential phosphonate producer strains. Subsequent bioassays with a phosphonate-sensitive *E. coli* test strain showed activity for 17 strains, and ^31^P NMR spectroscopic analysis of culture supernatants confirmed phosphonate production for 21 strains, including the rare actinomycete *Kitasatospora fiedleri* DSM 114396^T^. The functionality of the unique *K. fiedleri* P-BGC was verified by *pepM* gene deletion, which abolished phosphonate production in *K. fiedleri*, whereas overexpression of a cluster-situated LuxR-like regulator improved phosphonate production. These findings highlight the P-NP biosynthetic potential of actinomycetes and pave the way for discovering novel bioactive phosphonates.

## Abstract 2

Screening 940 genome-sequenced actinomycetes revealed 54 strains with phosphonate BGCs; 17 inhibited a phosphonate-sensitive *E. coli* test strain, and 24 are likely novel phosphonate producers.

## Graphical abstract

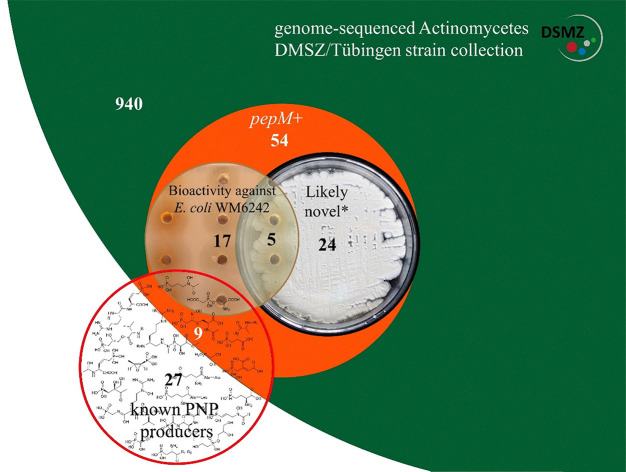

## Introduction

The rediscovery of known antibiotics and other specialized secondary metabolites poses a major challenge to the field of natural product (NP) discovery and development.^1^ The majority of antibiotics, as well as a range of anticancer, antifungal, and immunosuppressive agents were identified between 1940 and 1970 by systematically screening soil microorganisms for production of bioactive substances.^2^ This once successful pipeline is exhausted due to the frequent rate of rediscovery of known compounds.^3^ The reasons for this are multifaceted and include the reliance on common sampling sites, the use of standard strain-isolation techniques, and conventional cultivation conditions, all of which often result in the re-isolation of known or closely related species with similar secondary metabolite biosynthesis profiles.^4–6^ Furthermore, standard extraction protocols typically involve a limited range of organic solvents which creates a bias favoring the extraction of hydrophobic compounds.^7–9^ In antimicrobial assays, extracts are usually tested against a panel of standard test organisms, such as *Escherichia coli*, *Bacillus subtilis*, *Staphylococcus aureus etc.*, which in turn also contributes to rediscovery.^10^ Additionally, compounds that demonstrate prominent mass spectrometry (MS) patterns and ultra-violet (UV) spectroscopy signals are often prioritized even though they may represent “low hanging fruits” in discovery.^6,11^ As a result, these interconnected practices create a cycle leading to the rediscovery of previously known NPs, accentuating the necessity of innovative approaches to overcome these challenges.

Despite these challenges, the remarkable potential of microorganisms for NP discovery remains undeniable. The most versatile and prolific antibiotic producers belong to the phylum *Actinobacteria*, particularly to the genus *Streptomyces*.^12–14^ Most antimicrobial compounds commonly used clinically today are derived from these organisms.^2^ In these microorganisms, all genes required for the biosynthesis of a particular NP, as well as genes involved in regulation, secretion, and self-resistance mechanisms, are typically located together in a continuous region of the genome, comprising the so called biosynthetic gene cluster (BGC), an arrangement first discovered in 1979 by Rudd and Hopwood for the genes responsible for actinorhodin biosynthesis in *Streptomyces coelicolor* A3(2).^3,15,16^ Despite the extensive exploration of actinomycetes for NP production for the last 70 years, the advent of the genomics era has revealed that many novel compounds remain to be discovered.^17,18^ Current estimates indicate that only about 3% of the genomically encoded bacterial biosynthetic diversity has been experimentally characterized, with streptomycetes in particular representing a largely untapped genetic reservoir for novel NP biochemistry.^18^ Genome mining has played a pivotal role in bridging this gap by correlating genomic data with biosynthetic functions of encoded enzymes, and even in predicting the chemical structures of the resulting metabolic products.^5,19^ However, only certain types of BGCs allow for straightforward and reliable prediction of biochemistry and metabolic products by bioinformatics analysis of genomic sequence, including BGCs encoding polyketide synthases (PKS) and non-ribosomal peptide synthetases (NRPS).^20,21^ For other kinds of biosynthetic pathways, specific marker genes can serve as molecular footprints, enabling the identification of distinct structural features and underexplored classes of specialized metabolites, thereby facilitating the discovery of novel NP chemistry.^22^

One underexplored class of specialized metabolites are phosphonate-containing NPs (P-NPs). The unifying feature of P-NPs is the direct carbon–phosphorus bond. Early discovery efforts using bioassay-guided fractionation for isolating P-NPs yielded a commercialization orders of magnitude higher than the estimated 0.1% for NPs overall,^2,40^ highlighting the potential of small molecule P-NPs for pharmaceutical^41^ and agricultural applications.^42^ The global phosphonate market is set to grow, with many phosphonates demonstrating strong bioactivities suitable for various applications.^43,44^ One example is FR900098, a derivate of fosmidomycin, which was originally isolated from *Streptomyces rubellomurinus*.^23,24^ FR900098 is a candidate for malaria treatment due to its capability to inhibit the 1-deoxy-d-xylulose-5-phosphate (DXP) reductoisomerase in the non-mevalonate pathway for isoprenoid synthesis in *Plasmodium falciparum*.^25,26^ Fosfomycin,^27–29^ produced by *Streptomyces wedmorensis* DSM 41676^T^, *Streptomyces fradiae*, and *Pseudomonas syringae*,^30,31^ is a broad-spectrum antibiotic that has been used since the 1970s to treat various bacterial infections, including urinary tract and gastrointestinal infections.^32^ Fosfomycin targets the initial step of peptidoglycan biosynthesis by mimicking phosphoenolpyruvate (PEP), the natural substrate of UDP-*N*-acetylglucosamine enolpyruvyl transferase (MurA).^33–35^ P-NPs include phosphonates and phosphinates, with the latter featuring C–P–C or C–P–H bonds, and thereby phosphorus in an even lower redox state. Illustrative examples include the industrially used herbicide phosphinothricin tripeptide (PTT), also known as bialaphos, which is produced by *Streptomyces viridochromogenes* DSM 40736 and *Streptomyces mooreae* DSM 41527, formerly *S. hygroscopicus.*^36–38^ PTT acts as an inhibitor of glutamine synthetase by releasing the unusual amino acid phosphinothricin (PT), which mimics the native substrate glutamic acid, leading to bactericidal, fungicidal, and herbicidal effects.^36,39^ These compounds exemplify the diverse bioactivities of P-NPs, showcasing their potential as small molecular inhibitors.

Phosphonates and phosphinates frequently display structural similarities to primary metabolites such as phosphate esters, carboxylic acids, or other tetrahedral structures. This similarity underpins their bioactivity through “molecular mimicry”, allowing phosphonates to bind the same enzymatic partners as their cognate primary metabolites and act as small molecular inhibitors across various pathways, due to the widespread occurrence of this structural feature in biology.^44^ Furthermore, the unique P–C bond requires specialized catabolic pathways for the degradation into phosphates, preventing common degradation routes that involve the hydrolysis of phosphate esters or carboxylic acids.^45^

**Fig. 1:**
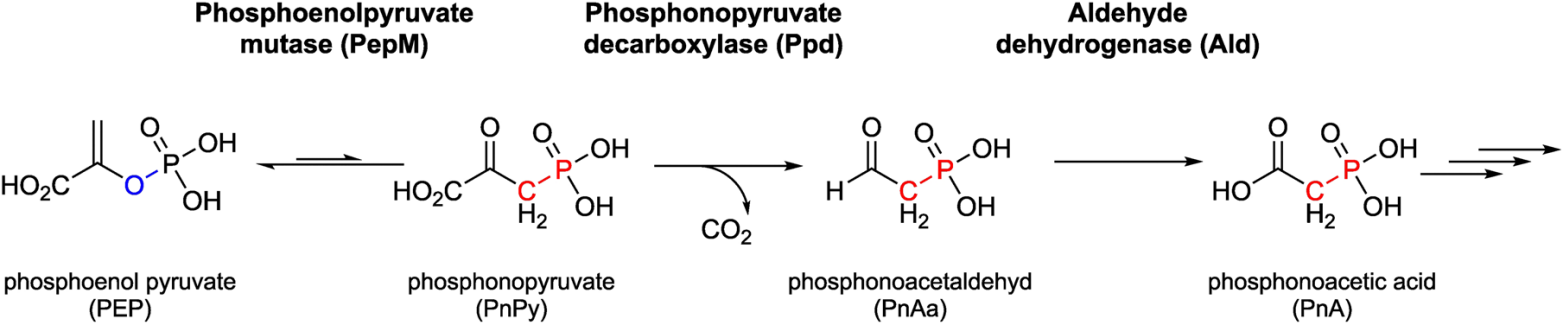
Phosphoenolpyruvate mutase (PepM) catalyzes the initial step shared in phosphonate biosynthesis creating the carbon–phosphorus bond by isomerization of phosphoenolpyruvic acid (PEP) to phosphonopyruvic acid (PnPy). Subsequent steps leading to phosphonoacetic acid (PnA), a significant branchpoint in phosphonate biosynthesis, are depicted: Phosphonopyruvate decarboxylase (Ppd) converts PnPy to phosphonoacetaldehyde (PnAa), which is then oxidized to PnA by an aldehyde dehydrogenase (Adh).

With a single exception,^46,47^ all known natural phosphonate biosynthetic pathways have a common initial biosynthetic step, namely the isomerization of phosphoenolpyruvate (PEP) to phosphonopyruvate (PnPy) by the enzyme phosphoenolpyruvate mutase (PepM)^48^ (Fig. 1). Due to the difference in P–O and P–C bond energies, this isomerization favors the formation of PEP at equilibrium. To generate the phosphonate product, the first reaction must be coupled to a subsequent enzymatic step that ensures thermodynamic favorability.^48^ This reaction is most commonly carried out by the PnPy decarboxylase (Ppd), which converts PnPy to phosphonoacetaldehyde (PnAa) while releasing a carbon dioxide molecule^49^ (Fig. 1). Phosphonate biosynthetic pathways show significant divergence after the first one or two biosynthetic steps.^49^ This lack of a widely conserved biosynthetic route is also reflected in the diverse gene composition of P-BGCs. As a result, predicting phosphonate products solely based on gene sequence information is often challenging.^50^ However, it has been found that the phylogeny of the conserved enzyme PepM strongly correlates with the *pepM* gene neighborhood, providing insights into phosphonate biosynthetic routes and products.^51^ Conversely, PepM phylogeny does not align with organismal phylogenies, indicating that horizontal gene transfer is a significant driver for the acquisition of P-BGCs.^51^ Due to the conserved nature of the initial step of phosphonate biosynthesis, together with the presence of the conserved motif EDKX_5_NS in the primary sequence of PepM, which distinguishes it from other members of the isocitrate lyase mutase protein family,^52–54^ the *pepM* gene represents a suitable marker to screen for novel phosphonate producers.

PepM-based genome mining screening has been successfully applied to 10 000 actinomycetes, resulting in the discovery of 19 novel P-NPs, including valinophos, which originated from *Streptomyces durhamensis* DSM 40539^T^,^55,56^ argolaphos, an antimicrobial that was first isolated from *Streptomyces monomycini* DSM 41801^T^,^55,57^ and phosphonocystoximate.^55^ Recently, additional novel P-NPs have been identified through PepM-based genome mining,^49,55–61^ such as a novel phosphonopeptide from *Bacillus velezensis*,^61^ a group of compounds termed phosphonoalamides isolated from *Streptomyces* sp. B-2790, *Streptomyces kutzneri* DSM 40907 and *Bacillus subtilis*,^49,60,62,63^ the antibiotic phosacetamycin produced by *Streptomyces aureus*,^58^ and the herbicide pantaphos from *Pantoea ananatis*.^59,64^ With the growing knowledge of phosphonate biosynthetic pathways, combined with the characterization of P-BGCs and their modification through genetic engineering, a strong foundation has been established for synthetic biology approaches.^65,66^ For example, the well-known herbicidal phosphonate glyphosate can now be obtained by fermenting *Streptomyces lividans* that expresses six genes from the argolaphos BGC to produce the precursor aminomethylphosphonate, which is then converted to glyphosate in one chemical step under aqueous conditions.^66^

Here, we present our results on the investigation of the phosphonate biosynthetic potential of 940 genome-sequenced actinomycetes from the DSMZ and University of Tübingen strain collections, focusing on an unexplored branch of phosphonate biosynthesis in the rare actinomycete strain *Kitasatospora fiedleri* DSM 114396^T^ as a representative producer.

## Results and discussion

### PepM screening leads to the identification of 45 unexplored phosphonate producers

In an effort to identify novel P-NPs, 940 actinomycetes genomes from the DSMZ (*n* = 781) and Tübingen (*n* = 159) strain collections were screened for the presence of *pepM*-like genes. Fifty-four strains (5.74%) from the two collections were found to contain PepM-encoding genes, defined as homologous proteins containing a perfect match for the conserved PEP mutase motif EDKX_5_NS (Table S1). From this subset of strains, nine had already been described as producers of P-NPs, including the ones mentioned above along with the phosalacine producer *Kitasatospora phosalacinea* DSM 43860^T^, 2-phosphinomethylmalic acid and desmethylphosphinothricin producer *Nonomuraea candida* DSM 45086^T^, and *Stackebrandtia nassauensis* DSM 44728^T^, a producer of phosphonoglycans. The remaining 45 strains did not show any association with known P-NPs, warranting further investigation.

### Prioritization of potential novel P-NP producers

**Fig. 2:**
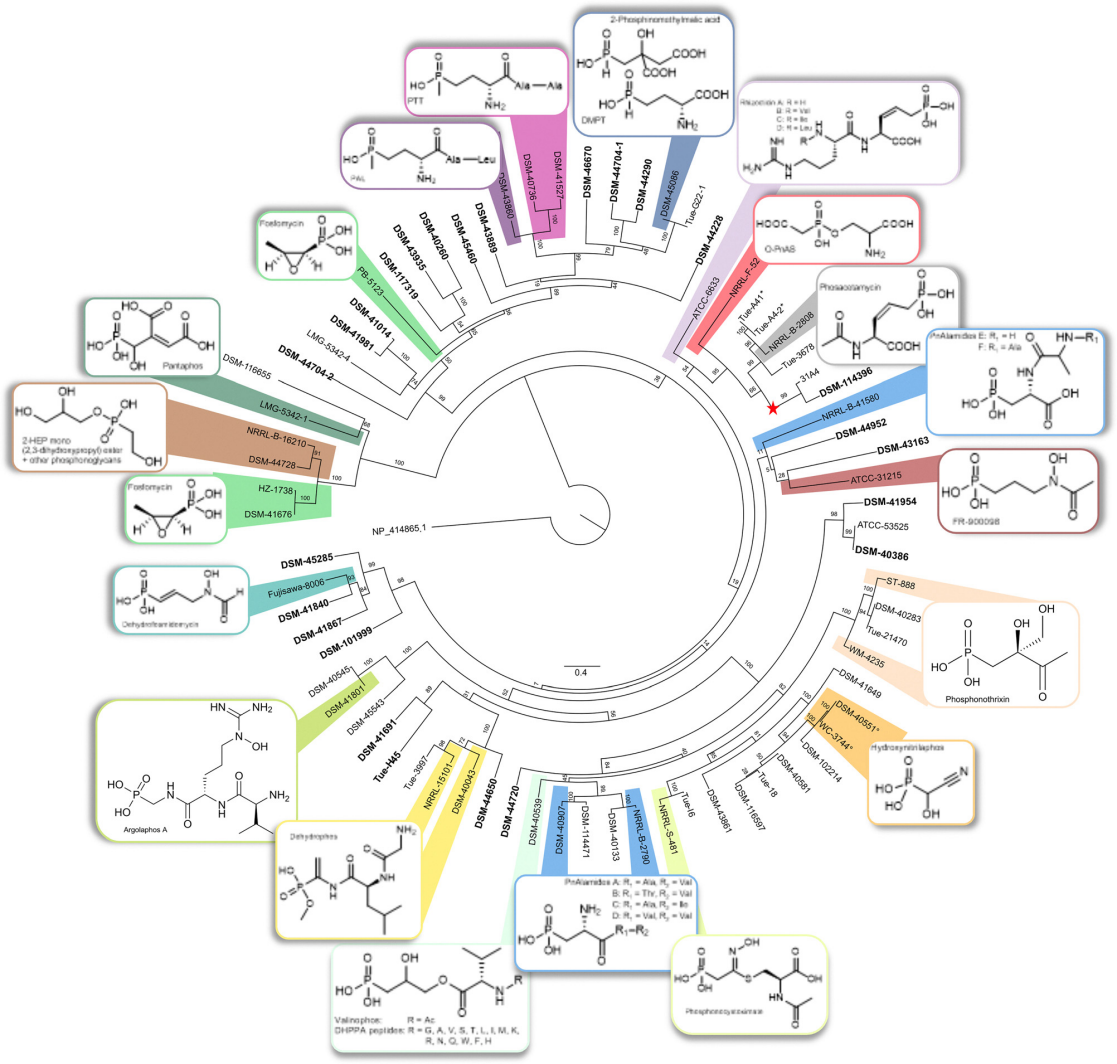
Maximum-likelihood tree of PepM amino acid sequences, inferred under the LG + I + G4 + F model and 1000 bootstrap iterations using RAxML v. 8.2.12,^84,85,91^ rooted by the outgroup 2-methylisocitrate lyase (NP_414865.1). The maximum likelihood (ML) phylogeny was inferred from a pairwise alignment generated using MAFFT v.7.^82,83^ The branches are scaled in terms of the expected number of substitutions per site. The numbers above the branches are support values from ML bootstrapping which were calculated with branch lengths. Nodes with sequences from known P-NP producers are color-coded and chemical structures of known products are displayed. Strain designations highlighted in bold are likely members of novel and unique Gene Cluster Families (GCFs). This classification is based on their lack of association with sequences from known P-NPs producers, as determined by a similarity cutoff of 0.612 in the BiG-SCAPE analysis. DHPPA = 2,3-dihydroxypropylphosphonate; 2-HEP = 2-hydroxyethylphosphonate; *O*-PnAS = *O*-phosphonoacetic acid serine; PAL = phosalacine; PnAlamides = phosphonoalamides; PTT = phosphinothricin tripeptide. The node containing the PepM sequence of *K. fiedleri* DSM 114396^T^, a putative novel producer of a phosphonoacetic acid-derived P-NP, was marked by a red star. *°PepM sequence from TÜ A41 is identical to TÜ A4-2 and PepM sequence from DSM 40551^T^ is identical to WC 3744.

To prioritize potential producers of novel P-NPs, we analyzed the PepM amino acid sequences for phylogenetic diversity. By including sequences from known phosphonate producers, we were able to assign clusters of high similarity to known compounds (Table S1). Our analysis revealed distinct phylogenetic branches, with several branches containing sequences from known P-NP producers, suggesting that phylogenetic proximity might be used as a criterion for identifying producers of known P-NPs (Fig. 2). For instance, one branch encompassed the known phosphonoalamide producer strains *Streptomyces* sp. NRRL B-2790^49^ and *Streptomyces kutzneri* DSM 40907,^62^ as well as *Streptomyces resistomycificus* DSM 40133^T^ and *Streptomyces badungensis* DSM 114471, suggesting the latter two may also produce phosphonoalamides. Another branch includes the phosphonothrixin producers *Streptomyces* sp. WM4235^67^ and *Saccharothrix* sp. ST-888,^68^ as well as *Streptomyces* sp. TÜ 21470 and *Kitasatospora purpeofusca* DSM 40283^T^; for the latter strain, an *ftx* gene cluster responsible for synthesizing phosphonothrixin has been reported.^67^ A branch characterized by low genetic divergence in the tree, as evidenced by short branch lengths, contained the hydroxynitrilaphos producer *Streptomyces regensis* NRRL WC-3744,^69^ along with five *Streptomyces* strains that are all likely hydroxynitrilaphos producers. The phylogenetic proximity of this branch to the phosphonocystoximate producer *Streptomyces* sp. NRRL S-481 aligns well with the previously reported similarity between the biosynthetic pathways leading to hydroxynitrilaphos and phosphonocystoximate and between their P-BGCs (Fig. 2).^55^ Only one *pepM* gene was found per genome, except for three strains - *Streptomyces* sp. I6, *Nocardia tenerifensis* DSM 44704^T^, and the pantaphos producer *P. ananatis* LMG 5342 - each containing two distinct P-BGCs with their own *pepM* gene, adding support to the hypothesis that PepM is a good marker for P-NP chemistry, since the same PepM does not seem to be shared by two different pathways in the same organism.

To support these findings and to further explore the uniqueness of the identified P-BGCs, a gene cluster network analysis was performed with BiG-SCAPE 2.0.^70^ All P-BGCs predicted by antiSMASH 7.1.0^53^ for the strains of the DSMZ and University of Tübingen collections, including 54 putative P-NP producers and 18 additional known producers previously reported in literature, were included in the analysis. Using a cutoff value of 0.612, we identified 45 gene cluster families (GCFs), of which 28 were singletons (Fig. S1 and Table S1). At this stringency, the resulting GCFs reflected established relationships among phosphonate-producing organisms: BGCs encoding structurally related phosphonates *via* similar biosynthetic pathways grouped together, whereas more divergent clusters remained separated. The largest cluster at this threshold comprised seven P-BGCs (GCF01), some of which were closely related to BGCs in GCF02 and GCF03, reflecting the high similarity shared between the pathways.^55^ Among the strains with these GCFs are the known hydroxynitrilaphos producer *Streptomyces regensis* NRRL WC-3744^69^ (GCF01) and the phosphonocystoximate producer *Streptomyces* sp. NRRL S-481^55^ (GCF02). The network analysis also enabled the detection of previously characterized P-BGCs in new species. For example, GCF12 linked the known pantaphos P-BGC from *P. ananatis* LMG 5342 with a previously unknown type-VI Hvr-like P-BGC in *Streptomyces thermoviolaceus* DSM 116655 (Fig. S1). In our PepM tree, the *S. thermoviolaceus* and *P. ananatis* sequences clustered as nearest neighbors, reflecting the limited distribution of *hvr* operon homologs in proteobacteria and actinobacteria, as also shown by Polidore *et al.*^59^

Five GCFs, each containing two P-BGCs, were not connected to any known producer in the network analysis. Among the 28 identified singletons, only ten corresponded to known P-NP producers, such as the valinophos producer *Streptomyces durhamensis* DSM 40539^T^ or the FR900098 producer *Streptomyces rubellomurinus* ATCC 31215.Overall, sequences from 24 strains across 22 GCFs/singletons, out of a total of 45 GCFs, showed no network connections to any known producers, suggesting they encode novel P-BGCs with low similarity to characterized ones. Therefore, these 24 strains were identified as likely producers of novel P-NPs (Fig. S1).

To assess P-NP production, 35 potential P-NP producers (the subset that had genome data available with the exclusion of biosafety level 2 strains) were cultivated on a panel of eight to ten different solid cultivation media, and antimicrobial assays by agar-block diffusion were conducted using the phosphonate-sensitive *Escherichia coli* strain WM6242,^24^ as well as *E. coli* K12 (parental control) and *Kocuria rhizophila* DSM 348 as Gram-negative and Gram-positive test organisms, respectively (Table S2). *K. rhizophila*, formerly known as *Micrococcus luteus*, is commonly employed in antibiotic susceptibility testing and for the detection of antibiotic residues in food products. The fosfomycin producer *Streptomyces fradiae* DSM 40943 was included as a positive control. Culture supernatants from *S. fradiae* DSM 40943 exhibited stronger activity against the phosphonate-sensitive *E. coli* WM6242 strain than against *E. coli* K12, suggesting that the observed inhibition was likely due to the production of fosfomycin, expected to be more active against WM6242 due to the induced expression of a phosphonate uptake system.^24,71,72^ Of the strains sharing a GCF with known phosphonate producers in the gene cluster network analysis, 19 were tested, of which 12 showed activity against *E. coli* WM6242, and seven of them additionally exhibited activity against *E. coli* K12. Given the prediction that these strains likely produce P-NPS similar to known compounds, this may reflect an investigation bias towards readily produced P-NPs. Of the 16 presumed novel P-NP strains included in the bioassay tests, five showed bioactivity against *E. coli* WM6242. Notably, all five strains that inhibited *E. coli* WM6242 also showed weaker activity against *E. coli* K12. This suggests either enhanced uptake of a single antimicrobial compound – such as a phosphonate – by *E. coli* WM6242, or the production of multiple bioactive metabolites, consistent with the high number of predicted BGCs per strain (36 on average, although some predictions may be influenced by contig fragmentation).

Eighteen strains showed no activity against the phosphonate-sensitive *E. coli* WM6242 under any tested conditions, suggesting that (i) the encoded P-NPs may lack antibiotic activity, (ii) were produced at low concentrations, (iii) the corresponding P-BGCs remained silent under the applied cultivation conditions, or (iv) that the phosphonate compound may not be active against *E. coli* WM6242 regardless of the overexpressed phosphonate uptake system (Table S2). Of the 18 strains, 12 exhibited antimicrobial activity against *K. rhizophila*, suggesting the production of non-phosphonate antimicrobials.

### Confirmation of phosphonate production by 21 actinomycetes using ^31^P NMR analysis

A subgroup of 26 strains was selected for phosphonate production analysis with ^31^P NMR. Phosphonate-containing compounds show characteristic ^31^P NMR chemical shifts above +8 ppm, in contrast to most other phosphorus-containing biomolecules, which typically resonate between −25 and +5 ppm. This distinct difference provides a reliable and convenient method to detect phosphonate compounds in complex culture samples.^40^ The 26 strains were grown in 10 different cultivation media and supernatants were subjected to ^31^P NMR analysis. 21 strains displayed spectra with chemical shifts consistent with phosphonates under at least two cultivation conditions. Confirmed phosphonate production included the known argolaphos producer *Streptomyces monomycini* DSM 41801^T^, along with other established phosphonate producers and associated producers, such as the recently identified phosphonoalamide producers *Streptomyces kutzneri* DSM 40907^62^ and *Streptomyces resistomycificus* DSM 40133^T^,^49^ as well as two strains from the largest cluster in the gene network analysis (GCF01-GCF03), likely associated with hydroxynitrilaphos or phosphonocystoximate production in *Kitasatospora setae* DSM 43861^T^ and *Kitasatospora atroaurantiaca* DSM 41649^T^, respectively. Phosphonate production was detected for nine out of the ten tested strains from the likely novel P-NP producer group (24 likely novel P-NP producer strains), including *Kitasatospora fiedleri* DSM 114396^T^, *Streptomyces aureocirculatus* DSM 40386^T^, and *Streptomyces iranensis* DSM 41954^T^ (Fig. S2–S6). For the latter (Fig. S3), we have recently demonstrated that inactivation of the *pepM* gene using a newly developed cloning vector system results in the loss of phosphonate production, proving the functionality of the identified P-BGC in *S. iranensis* DSM 41954^T^.^73^

### Characterization of the phosphonate biosynthetic gene cluster in the rare actinomycete *Kitasatospora fiedleri* DSM 114396

The rare actinomycete *Kitasatospora fiedleri* DSM 114396^T^ demonstrated phosphonate production, evidenced by characteristic chemical shifts in ^31^P NMR (Fig. S6). We recently described this strain as a novel type strain and published a high-quality genome assembly (NCBI accession GCA_948472415).^74^ In the PepM-phylogenetic tree, the node containing the PepM amino acid sequence from the *K. fiedleri* P-BGC did not cluster with any known P-NP producer (Fig. 2). Consistently, in the gene cluster network analysis, the *K. fiedleri* P-BGC clustered together with that from *Streptomyces* sp. 31A4^51,55^ in GCF09 but showed no connection to a cluster of any known P-NP producer (Fig. S1).

A gene synteny analysis performed with NCBI BLAST against the type strains of *Kitasatospora cineracea* DSM 44780^T^ and *Kitasatospora niigatensis* DSM 44781^T^, the most closely related type strains to *K. fiedleri* and lacking a P-BGC,^74^ suggested that the P-BGC most likely spans from position 3401998 (stop codon of CDS *kfp*01 with locus_tag QMQ26_RS15690 encoding a putative aldehyde dehydrogenase) to position 3428393 (start codon of CDS *kfp*25 with locus_tag QMQ26_RS15810 encoding a putative NUDIX domain-containing protein) (Fig. S7 and S11). An analysis with clinker^75^ of all putative P-BGCs highly similar to *K. fiedleri*'s (22 records in NCBI's “RefSeq Genome Database”, from 21 strains, 3 of which belong to *K. fiedleri* species) confirmed the above mentioned boundaries of the P-BGC (Fig. S8). The presence of this P-BGC across phylogenetically diverse members of the *Streptomycetaceae*, coupled with its absence in the closest phylogenetic neighbors *K. cineracea* and *K. niigatensis*, suggests that *K. fiederli* likely acquired the cluster *via* horizontal gene transfer. A detailed analysis of the P-BGC DNA sequence supports the accuracy of both the sequence and its gene annotation (Fig. S8 and S9). All genes present in the suggested P-BGC run in the same orientation, and with little or no intergenic space, indicating they might form an operon (Fig. S9). The gene encoding the putative PepM enzyme is located between position 3404408 and 3403515 of the deposited chromosome sequence (*kfp*02, locus_tag QMQ26_ RS15695). Notably, the cluster contains a gene *kfp*24, encoding a putative transcriptional regulator of the LuxR family (locus_tag QMQ26_RS15805) (Fig. 4 and Fig. S8). This gene is located immediately upstream of the BGC, shares the same transcriptional orientation as the downstream genes, and is separated from them by a 670 bp intergenic region, likely harboring the main promoter driving expression of the BGC. The *kfp*24 gene was also the only putative transcriptional regulatory gene identified within the entire genomic region proposed to encompass the complete P-BGC of *K. fiedleri*.

### Genetic manipulation of the P-BGC confirms phosphonate biosynthesis in *Kitasatospora fiedleri* DSM 114396^T^

**Fig. 3:**
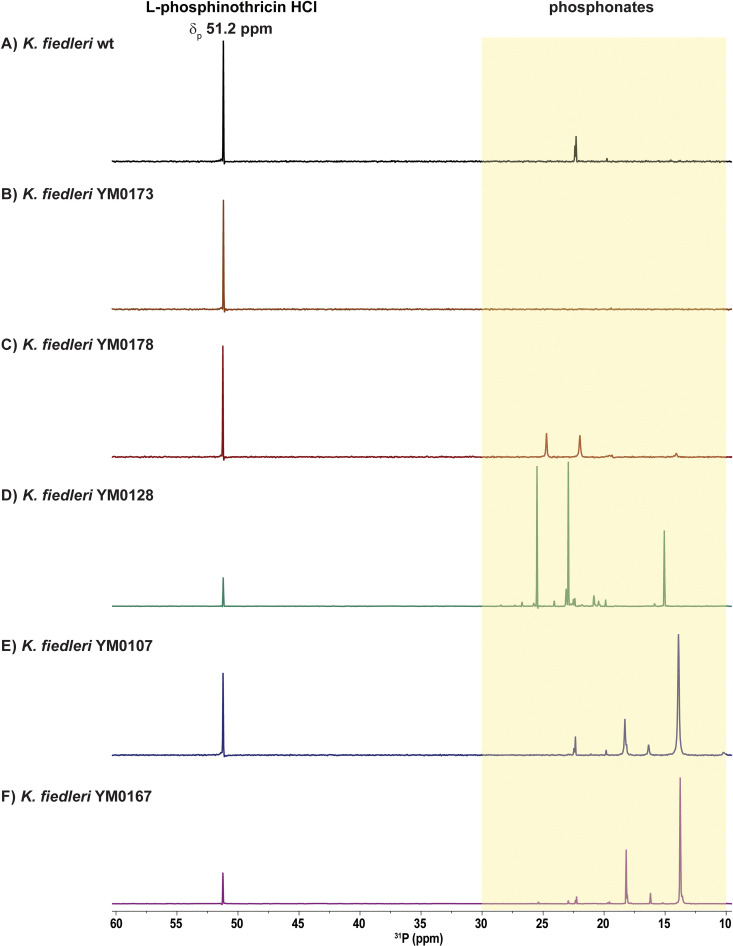
^31^P NMR spectra of concentrated culture supernatants from *Kitasatospora fiedleri* DSM 114396^T^ and mutant strains: (A) wildtype *K. fiedleri* strain. (B) Deletion mutant *K. fiedleri* Δ*pepM-ppdAB::neo* (YM0173). (C) Deletion mutant complemented with *pepM-ppdAB* (YM0178). (D) *pepM-ppdAB* overexpression mutant (YM0128). (E) *luxR*-overexpression mutant (YM0107). (F) *pepM-ppdAB* and *luxR*-overexpression mutant (YM0167). l-Phosphinothricin HCl (10 mM in H_2_O, *δ*_P_ 51.2 ppm) was used as a chemical shift reference.

To confirm the role of the identified *K. fiedleri* P-BGC in phosphonate biosynthesis, the genes *kfp*02-04, encoding the predicted first two steps of the pathway, *pepM-ppdAB*, were inactivated by replacement with a kanamycin resistance gene (*neo*) *via* homologous recombination with construct pDS0107 (Table S3). PCR analysis confirmed the gene replacement, verifying the successful generation of the deletion mutant *K. fiedleri* Δ*pepM-ppdAB*::*neo* (YM0173). Under the previously defined production conditions, YM0173 failed to produce any compounds with phosphonate-specific peak patterns in the ^31^P NMR (Fig. 3B). Complementation of the deletion mutant with ectopic expression of *pepM-ppdAB* (*kfp*02-*kfp*04), using pRM4- (pDS0105) or pIJ10257-based constructs (pDS0106), restored phosphonate production, as measured by ^31^P NMR (Fig. 3C). These results confirmed that the identified P-BGC is responsible for the production of P-NP compound(s) in *K. fiedleri*.

A common strategy to enhance metabolite production is the overexpression of genes encoding the first enzymes of the pathway. Thus, we mobilized the *pepM-ppdAB* overexpression constructs pDS0105 (pRM4_*kfp*02-*kfp*04) and pDS0106 (pIJ10257_*kfp*02-*kfp*04) to *K. fiedleri* by conjugation. Cultivation of the resulting strains led to a differential peak pattern in ^31^P NMR, with increased signal intensity and the appearance of new chemical shifts relative to the wild-type strain (Fig. 3A and D). To establish the connection between these three enzymes and phosphonate production, we introduced pDS0105 and pDS0106 into the heterologous hosts *Streptomyces albus* and *Streptomyces lividans*, which do not inherently produce phosphonates. Following cultivation, phosphonate-specific chemical shifts were observed for the heterologous expression strains *S. albus* and *S. lividans* in ^31^P NMR, confirming the role of the introduced genes in phosphonate biosynthesis (see SI Fig. S10).

As detailed above, we identified *kfp*24 as a gene encoding a putative pathway-specific transcriptional regulator of the LuxR-family. Members of this family typically function as transcriptional activators. Therefore, it was expected that overexpression of *kfp*24 should lead to overproduction of the metabolic product of the pathway. Thus, the gene *kfp*24 was cloned under the control of the constitutive promoter *ermE*p* in either pRM4 or pIJ10257 integrative vectors (resulting in pDS0101 and pDS0102, respectively) (Table S3). The *kfp*24-overexpression constructs pDS0101 and pDS0102 were mobilized to *K. fiedleri* wild-type to generate strains YM0107 and YM0108, respectively. Subsequently, the *kfp*02-*kfp*04 (*pepM-ppdAB*) overexpression constructs pDS0105 and pDS0106 were introduced into YM0107 and YM0108, respectively, resulting in strains YM0167 and YM0168, respectively (Tables S3 and S4). ^31^P NMR analysis of culture supernatants from all these mutants resulted in increased and new phosphonate-specific chemical shifts in ^31^P NMR, supporting that the LuxR-family regulator Kfp24 is the pathway-specific transcriptional activator of the P-BGC (Fig. 3E and F). Here, ^31^P NMR signals are interpreted strictly as evidence for phosphonate biosynthesis, leaving the precise accumulation of the terminal product and the full extent of the Kfp24 regulon to be determined. Furthermore, we intentionally avoided making strong claims about bioactivity because the assays were performed on complex extracts, and it would be inappropriate to attribute activity to the phosphonate without unambiguous compound-level confirmation. While the precise structure and bioactivity of the phosphonate compound(s) produced by *K. fiedleri* remains under investigation, our combined genetic, bioinformatic, and ^31^P NMR data unequivocally demonstrate the production of a phosphonate metabolite. Isolation and structure elucidation efforts are ongoing and will be reported separately.

### *K. fiedleri* DSM 114396^T^ contains a unique P-BGC putatively linked to a phosphonoacetic acid-derived P-NP pathway

**Fig. 4:**
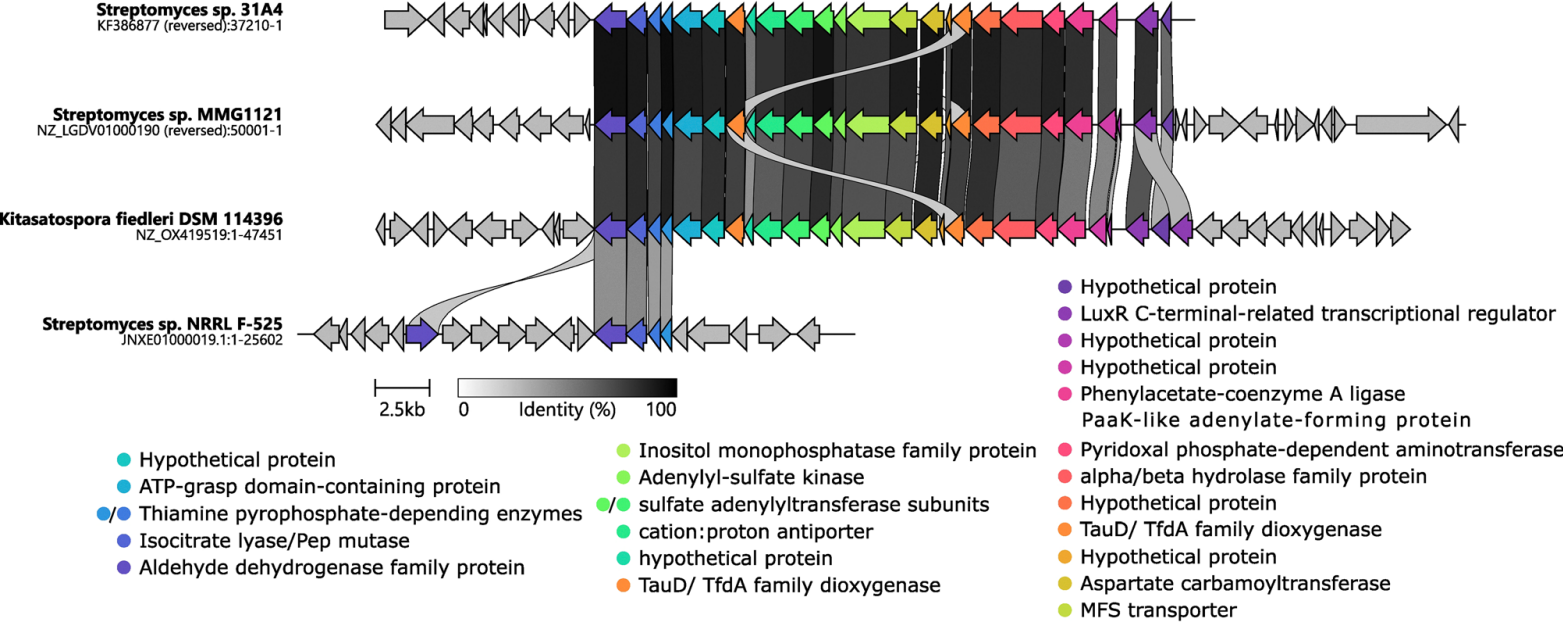
Clinker alignment of the P-BGC of *K. fiedleri* DSM 114396^T^ and previously identified similar P-BGCs from Streptomyces sp. 31A4, Streptomyces sp. MMG112, and the well characterized P-BGC from Streptomyces sp. NRRL F-525. The orientation of the genes is given as for *K. fiedleri* chromosome sequence NZ_OX419519. The four first genes of the P-BGC, the aldehyde dehydrogenase, PEP mutase, and two encoding the phosphonopyruvate decarboxylase, are fully conserved in arrangement across all four P-BGCs. The second putative aldehyde dehydrogenase found in proximity in Streptomyces sp. NRRL F-525 is also highlighted due to the high identity (discussed with Fig. S12).

*K. fiedleri* DSM 114396^T^ harbors a unique P-BGC that network analysis placed alongside *Streptomyces* sp. 31A4 within GCF09 (Fig. S1). PepM from *Streptomyces* sp. 31A4 clustered very closely together with PepM from *Streptomyces* sp. MMG1121,^55^ and all three P-BGCs share the same synteny with high sequence identity (Fig. 4 and Fig. S8A–D). All three P-BGCs carry genes encoding the canonical PepM accompanied by Ppd (and in all cases this enzyme is encoded by two separate genes). Notably, all three P-BGCs also carry a gene encoding a putative aldehyde dehydrogenase (Adh), which, in the case of *K. fiedleri* (gene *kfp*01), we propose as one of the boundaries of the P-BGC, as detailed above and in the SI. The amino acid sequence of Kfp01 shares 82.32%, 81.88%, and 57.04% identity with the homologs from *Streptomyces* sp. 31A4, *Streptomyces* sp. MMG1121, and *Streptomyces* sp. NRRL F-525, respectively. The later has been investigated by Freestone *et al.*, 2017 which found the strain to be a producer of *O*-phosphonoacetic acid serine (*O*-PnAS) but the P-BGC that is shared between *K. fiedleri*, *S.* sp. 31A4 and *S.* sp. MMG1121 clearly extends beyond the P-BGC responsible for *O*-PnAS production in *S.* sp. NRRL F-525 (Fig. 4). All of these predicted enzymes have fully conserved catalytic and phosphonate-binding pocket residues, as compared to the well-characterized enzyme PhnY from *Sinorhizobium meliloti* 1021 (Fig. 4 and Fig. S6).^76^ Based on this conservation, and as previously proposed for *Streptomyces* sp. 31A4,^65^ we propose that the *K. fiedleri* DSM 114396^T^ P-BGC encodes the biosynthesis of a phosphonate natural product with phosphonoacetic acid as an intermediate, as depicted in Fig. 1.

### A highly conserved subcluster encodes 3'-phosphoadenosine-5'-phosphosulfate metabolism

The gene products of *kfp*10 to *kfp*12 show high sequence similarity to widely conserved bacterial enzymes, including two putative sulfate adenylyltransferase subunits (*kfp*10, *kfp*11), and an adenylyl-sulfate kinase (*kfp*12). Along with *kfp*13, encoding a putative inositol monophosphatase family protein, this subcluster shows similarity to the *cysDNCQ* genes in *E. coli*^77^ based on predicted amino acid sequence comparisons. The encoded enzymes are involved in synthesizing and recycling the universal sulfur donor 3′-phosphoadenosine-5′-phosphosulfate (PAPS), a key precursor for cysteine and methionine biosynthesis. The predicted functions for *kfp*10-13 indicate that all genes necessary for the synthesis, accumulation and recurrent utilization of PAPS are present in the *K. fiedleri* P-BGC. Although associated with primary metabolism, this set of enzymes may have adapted to function in specialized metabolism. Notably, components of the *cysDNCQ* genes have been identified in the BGCs of mitomycin C and azinomycin B, suggesting a role in specialized metabolism.^78,79^ Such evolutionary transitions from primary to specialized metabolism have been reported before; for example, in *S. viridochromogenes* the aconitase-like enzyme Pmi has evolved to specialize in PTT synthesis, likely diverging from the typical TCA cycle function carried out by its counterpart, AcnA.^80^

Furthermore, BLASTp analysis of the amino acid sequences of four genes − *kfp*05, *kfp*07, *kfp*17, and *kfp*20 − in the *K. fiedleri* P-BGC predicted their function as putative enzymes, involved in previously characterized phosphonate biosynthetic pathways.^51,58,60,63,78^ This included *kfp*05, which is predicted to code for ATP-grasp-domain-containing protein. ATP-grasp proteins are known for catalyzing ATP-dependent peptide bond formation between amino acids, as well as for mediating unique reactions such as esterification of valine onto a primary alcohol by VlpF in the valinophos producer *S. durhamensis*, resulting in the formation of 2,3-dihydroxypropylphosphonate-valine (DHPPA-Val) (Fig. S13A).^56,58,60,63^ In the *K. fiedleri* P-BGC, the *kfp*07 and *kfp*17 genes encode putative TauD/TfdA family dioxygenases. Based on previous studies on *Burkholderia pseudomallei*, these enzymes are proposed to hydroxylate PnA to 2-hydroxy-phosphonoacetate (2-HPnA), suggesting a role of 2-HPnA as an alternative head group for phosphonolipids in *Burkholderia* species (Fig. S13B).^51^ Additionally, *kfp*20 encodes a putative pyridoxal phosphate (PLP)-dependent aminotransferase. This type of enzyme is known to function as amino-group donors and has been implicated in phosphonopeptide synthesis, specifically catalyzing the transamination of PnPy to phosphonoalanine (PnAla) during phosphonoalamide biosynthesis (Fig. S13C).^49,63^ Altogether, the predicted functions of the *K. fiedleri* P-BGC genes suggest that the cluster encodes a phosphonopeptide biosynthesis pathway derived from PnA. However, for a substantial proportion of the BGC genes, the subsequent steps in biosynthesis remain unclear, suggesting that the cluster-encoded product may represent a novel P-NP.

## Conclusion

Our investigation focused on uncovering novel P-NP producers from actinomycetes of the DSMZ and Tübingen strain collection. Screening 940 genome-sequences revealed 54 strains harboring *pepM*, of which 45 represent previously uncharacterized P-NP producers. Based on PepM phylogeny and gene cluster network analysis, we prioritized 24 strains with unique biosynthetic potential. Bioassays confirmed bioactivity for 17 of putative P-NP producer strains. ^31^P NMR analysis of culture supernatants confirmed the production of phosphonates in 21 actinomycetes, nine of which identified as potential producers of novel P-NPs through bioinformatic analysis, supporting the effectiveness of our approach. Among these, the rare actinomycete *K. fiedleri* DSM 114396^T^ was selected for further analysis due to its unique P-BGC. Deletion of the *pepM*-like gene *kfp*02 BGC abolished phosphonate production, whereas genetic complementation restored it, as confirmed by ^31^P NMR, thereby validating the functionality of the P-BGC in phosphonate biosynthesis. Furthermore, overexpression of a cluster-situated LuxR-like regulator gene (*kfp*24) and minimal P-BGC genes (*kfp*02-04) improved the phosphonate-specific signal pattern in ^31^P NMR. Detailed cluster analysis points to a phosphonopeptide as the likely biosynthetic product.

Altogether, our results illustrate the effectiveness of combining genome-based phylogenetic analysis, gene cluster networking, and bioassays to prioritize and explore strains for novel phosphonate discovery. Phosphonate isolation and structure elucidation is ongoing and will be reported in due course.

## Experimental

### Identification of genome-sequenced actinomycetes harboring a P-BGC

A total of 940 genome sequences from the DSMZ and Tübingen actinomycetes strain collections were screened for the presence of P-BGCs using antiSMASH (versions 6.0 to 7.1.0;^53,81^ the specific version deployed on the date of genome availability). Screening was performed manually as new sequencing data became available, with each genome reviewed for predicted P-BGCs and phosphonate-like clusters.

### Identification of *pepM* genes

A total of 72 genome sequences of the studied strains (*n* = 54) and additional known phosphonate producers (*n* = 18) were subjected to antiSMASH analysis version 7.1.0,^53^ using GenBank annotation files from the assemblies published with NCBI as input and relaxed strictness settings. Sequences from *Streptomyces* sp. 31A4 and *Streptomyces silvensis* ATCC 53525 were included in the analyses to explore branches of interest with low phylogenetic proximity to characterized P-NP producers. Neither strain is held by DSMZ or the University of Tübingen collections. The secondary metabolite clusters identified as phosphonate or phosphonate-like clusters were screened for the presence of the *pepM* gene using NCBI BlastP and evaluated to ensure that the encoded amino acid sequences conform to the consensus motif of PepM (EDKX_5_NS).

### Phylogenetic tree construction

A multiple sequence alignment of 74 PepM amino acid sequences and the sequence of 2-methylisocitrate lyase (NP_414865.1), which represents an outgroup to PepM, was created with MAFFT v.7 using the E-INS-I consistency-based method with default settings.^82,83^ Maximum likelihood (ML) phylogeny was interfered from the alignment with RAxML v. 8.2.12.^84^ For ML, rapid bootstrapping and subsequent search for the best tree was used. The best amino acid substitution matrix was determined with empirical frequencies with RAxML using a ML starting tree.^85^

### Network analysis of P-BGCs

The P-BGC regions (*n* = 74) that were predicted by antiSMASH 7.1.0 to yield a phosphonic acid product were clustered into GCFs with BiG-SCAPE version 2.0^70^ using a cut-off value of 0.612, including singletons in the output, and allowing the mixing of all classes of natural products (–mix). The BiG-SCAPE cutoff value (here, 0.612) sets the threshold for the maximal distance of links that group BGCs into families based on their overall similarity in domain content, arrangement, and sequence. The value was chosen to best reflect known relationships among P-NP producers—at this threshold, closely related clusters (with similar products and pathways) are grouped together. The gene cluster network obtained from BiG-SCAPE was visualized with Cytoscape v.3.10.3.

### Bacterial strains, plasmids and cultivation conditions

Plasmid vectors and constructs, and bacterial strains, are listed in Tables S3 and S4 respectively. pGus21 and pRM4 and their DNA sequence were a gift from Günther Muth^86^ (Eberhard Karls Universität Tübingen, Geschwister-Scholl-Platz, 72074 Tübingen, Germany). pIJ10257 was a gift from JIC StrepStrains (John Innes Centre, Norwich Research Park, NR4 7UH, Norwich, UK). pTC192km was as gift from Antonio Rodriguez (University of Leon, Spain). *Streptomyces albus* J1074 and *Escherichia coli* ET12567/pUZ8002^87,88^ were a gift from JIC StrepStrains. *Escherichia coli* WM6242 was a gift from William Metcalf (University of Illinois at Urbana-Champaign, USA). *Streptomyces lividans* T7 was sourced from the Tübingen strain collection. DSM strains were sourced from the German Collection of Microorganisms and Cell Cultures (Leibniz-Institut DSMZ - Deutsche Sammlung von Mikroorganismen und Zellkulturen GmbH, Inhoffenstraße 7 B, 38124 Braunschweig, Germany).

*E. coli* cultivation and general molecular biology techniques were performed following established methods^89^ and instructions provided by suppliers. *Streptomyces* strains were cultivated in SFM (MS) for preparation of spore stocks, and for mobilization of plasmid constructs to *Streptomyces* strains by conjugation from *E. coli*, all of which was done according to established methods.^90^ Apramycin and kanamycin were used at 50 mg L^−1^, fosfomycin at 20 mg L^−1^, final concentration.

Actinomycetes strains were cultivated for phosphonate production in the following solid media, for 7 days at 28 °C or until confluent growth was reached: OM medium (20 g oat meal, 1 g K_2_HPO_4_, 20 g agar and 5 mL trace metal mix 1 dissolved in 1 L distilled water, pH adjusted to 7.3. Trace metal mix 1 : 2 g FeCl_3_·6H_2_O, 0.1 g CaCl_2_·2H_2_O, 0.1 g MnCl_2_·2H_2_O, 0.1 g (NH_4_)_6_Mo_7_O_24_·4H_2_O, 0.1 g Na_2_B_4_O_7_·10H_2_O, dissolved in 1 L distilled water), HM medium (4 g yeast extract, 10 g malt extract, 4 g glucose and 1 g K_2_HPO_4_ and 16 g agar dissolved in 1 L distilled water, pH adjusted to 7.3), SFM medium (20 g mannitol, 20 g soy flour and 1 g K_2_HPO_4_, 20 g agar dissolved in 1 L distilled water, pH adjusted to 7.3), R5 medium (103 g saccharose, 10 g glucose, 0.25 g K_2_SO_4_, 10.12 g MgCl_2_, 0.1 g casamino acids, 5 g yeast extract, 5.73 g TES, 18 g agar and 2 mL trace metal mix 1 dissolved in 955 mL distilled water, pH adjusted to 7.3), NL200 medium (20 g mannitol, 20 g cornsteep powder and 1 g KH_2_PO_4_ and 16 g agar dissolved in 1 L distilled water, pH adjusted to 7.5), NL300 medium (20 g mannitol, 20 g cottonseed powder and 1 g KH_2_PO_4_ and 16 g agar dissolved in 1 L distilled water, pH adjusted to 7.5), NL400 medium (20 g mannitol, 20 g cottonseed powder and 1 g KH_2_PO_4_ and 16 g agar dissolved in 1 L distilled water, pH adjusted to 7.5), NL410 medium (10 g glucose, 10 g glycerol, 5 g oat meal, 10 g soy meal, 5 g yeast extract, 5 g casamino acids, 1 g CaCO_3_, 1 g KH_2_PO_4_ and 16 g agar dissolved in 1 L distilled water, pH adjusted to 7.0), NL500 medium (10 g soluble starch, 10 g glucose, 10 g glycerol, 15 g fish meal, 10 g sea salts and 16 g agar dissolved in 1 L distilled water, pH adjusted to 8.0 and rechecked after 1 h) and NL800 medium (5 g glucose, 10 g glycerol, 10 g soluble starch, 5 g soy flour, 2 g yeast extract, 1 g NaCl, 1 g CaCl_2_ and 16 g agar dissolved in 1 L distilled water, pH adjusted to 7.2).

### Bioassay with phosphonate-sensitive *E. coli* strain

Antibiotic activity was analyzed in agar-block diffusion assays using *E. coli* WM6242, *E. coli* K12, and *Kocuria rhizophila* DSM 348 as indicator organisms. Test plates were prepared in l-agar medium (for all strains including non-induced *E. coli* WM6242) or LB-G medium supplemented with IPTG to a final concentration of 200 µM (for phosphonate transporter induced *E. coli* WM6242). For *E. coli* strains, an overnight liquid culture of the indicator organism in L medium was used to inoculate a day culture (also in L medium), which was incubated at 37 °C and 180 rpm orbital shaking until reaching an OD_600_ of around 0.8; this day culture was used to inoculate the molted agar media at 0.1% (v/v) concentration. For *K. rhizophila* test plates, molted l-agar was inoculated with overnight culture at 0.1% (v/v) concentration. Agar blocks with a diameter of 8 mm were excised from sections of the plates displaying mature, confluent growth and placed on the test plates inoculated with the test organisms, followed by incubation overnight at 37 °C. After incubation, the diameters of all observed inhibition zones were measured. Due to the large number of samples, and the qualitative nature of the experiment, only one biological replicate was prepared for each condition.

### Molecular cloning

Restriction endonucleases, T4-DNA ligase, Q5 and One-Taq DNA polymerases, DNA Polymerase I-Large (Klenow) Fragment, were purchased from New England Biolabs (New England Biolabs GmbH, Brüningstraße 50; Geb. B852, (Industriepark Höchst), D-65926 Frankfurt am Main, Germany) and used according to provider's instructions. Sequences of oligonucleotides used in this study are given in Table S5.

### Construction of pDS0105 and pDS0106 for overexpression of *kfp*02–*kfp*04

The coding sequences of *ppdB*, *ppdA*, and *pepM* (*kfp*02, *kfp0*3, and *kfp0*4) were PCR amplified as a continuous DNA fragment, with the same sequence and arrangement as in the wild-type genome (Table S6); optimal oligonucleotides JP555 and JP556 were used as PCR primers with gDNA as template, and the JP555–JP556 PCR product was used as template for a PCR with oligonucleotides JP549 and JP550 as primers. The JP549–JP550 PCR product was first blunt-end cloned in pBluescript II KS(+) (pKS) linearized with SmaI. The insert was then excised with NdeI/HindIII and ligated to the expression vectors pRM4 or pIJ10257 linearized with the same enzymes, obtaining constructs pDS0105 or pDS0106, respectively. The correctness of all plasmids was verified by Sanger sequencing.

### Construction of pDS0107 for replacement of *kfp*02*–kfp*04 with *neo*

The upstream homologous region was PCR amplified with oligonucleotides JP553 and JP554 and blunt-end cloned in pKS linearized with SmaI; after confirmation of the correct insert by Sanger sequencing, a clone with the insert in orientation M13R-JP553 was selected to allow use the HindIII site of pKS. The downstream homologous region was PCR amplified with oligonucleotides JP551 and JP552, and blunt-end cloned in pKS linearized with SmaI; after confirmation of the correct insert by Sanger sequencing, a clone with the insert in orientation M13F-JP552 was selected. This construct was linearised with SpeI-XbaI and ligated with the upstream homologous region excised XbaI-HindIII and the kanamycin resistance marker neo excised with XbaI-HindIII from pTC192-km; the resulting cassette JP551-downstream-JP552_SpeI/XbaI_neo_HindIII_JP553-upstream-JP554_XbaI was excised from pKS with BamHI (from JP551 and carried from pKS polylinker) and ligated to pGus21 linearised with BamHI, resulting in construct pDS0107.

### Construction of pDS0101 and pDS0102 for overexpression of putative LuxR-like regulator gene *kfp*24 [pGE505 and pGE507]

*kfp*24 (putative LuxR-like regulator gene) was amplified by PCR with oligonucleotides JP525 and JP526, and blunt-end cloned in pKS linearized with SmaI. After confirmation of the correct insert by Sanger sequencing, it was excised with NdeI/HindIII and ligated to the expression vectors pIJ10257 or pRM4 linearized with the same enzymes, obtaining constructs pDS0101 or pDS0102, respectively.

### Construction of *K. fiedleri* DSM 114396^T^ Δ*kfp*02*kfp*04*::neo* deletion mutant (YM0173) and complemented strains (YM0178)

pDS0107 was mobilized to *K. fiedleri* DSM 114396^T^ by conjugation from *E. coli* ET12567/pUZ8002 and exconjugants selected for resistance to kanamycin. Several exconjugants were cultivated on HM without antibiotic to facilitate segregation through sporulation. Spores from exconjugants were plated at low density (100–150 CFU per plate) on HM supplemented with kanamycin and X-Gluc; GusA-negative colonies were replicated on HM supplemented with apramycin and X-Gluc to confirm loss of vector, and on HM with kanamycin and X-Gluc to confirm presence of the *neo* marker gene. Kanamycin-resistant, apramycin-sensitive, GusA-negative clones were selected for genotype test. Mutant clones were verified by multiple PCR reactions: with oligonucleotides pepMupfw/pepMuprv, RTpepM2fw/RTpepM2rv, JP569/JP570, JP571/JP572, JP573/JP574, and JP575/JP576 as primer pairs (no amplicon expected for mutants), JP508/JP503 and JP555/JP556 (differential amplicon size), JP581/JP582 and JP583/JP584 (exclusive mutant, one primer anneals within *neo*, the other outside homologous regions) (Table S5); all PCR products were assessed in gel electrophoresis and verified by Sanger sequencing with the same oligonucleotides as primers.

To test genetic complementation of the mutants, *kfp*02–04 expression constructs pDS0106 was mobilized to selected mutant clones by conjugation from *E. coli* ET12567/pUZ8002 and exconjugants selected for resistance to hygromycin B.

### Construction of *K. fiedleri* DSM 114396^T^*kfp*24 overexpression strains (YM0107 and YM0108)

pDS0101 or pDS0102 were mobilized to *K. fiedleri* DSM 114396^T^ by conjugation from *E. coli* ET12567/pUZ8002 and exconjugants selected for resistance to hygromycin B or apramycin, respectively.

### Construction of *K. fiedleri* DSM 114396^T^*kfp*02-04 overexpression strains (YM0128 and YM0129)

pDS0105 or pDS0106 were mobilized to *K. fiedleri* DSM 114396^T^ by conjugation from *E. coli* ET12567/pUZ8002 and exconjugants selected for resistance to apramycin or hygromycin B, respectively.

### Construction of *K. fiedleri* DSM 114396^T^*kfp*24 (putative LuxR-like regulator gene) and *pepMppdAB* overexpression strains (YM0167 and YM0168)

pDS0105 or pDS0106 were mobilized to *Kitasatospora* sp. YM0107 or YM0108, respectively by conjugation from *E. coli* ET12567/pUZ8002 and exconjugants selected for resistance to apramycin or hygromycin B, respectively.

### ^31^P NMR analysis for phosphonates detection

DSM 40043^T^, DSM 40133^T^, DSM 40283^T^, DSM 40386^T^, DSM 40581^T^, DSM 40907, DSM 41649^T^, DSM 41691^T^, DSM 41801^T^, DSM 41840^T^, DSM 41867^T^, DSM 41954^T^, DSM 43861^T^, DSM 44228^T^, DSM 45285^T^, DSM 46670^T^, DSM 101999^T^, DSM 102214^T^, I6, DSM 40736, DSM 114396^T^, TUE 18, TUE H45, TUE 3678, TUE 3997, TUE 21470 (for full species names see Table S1) were cultivated in 100 mL Erlenmeyer baffled flasks containing 30 mL of HM or R5 standard medium,^90^ with orbital shaking (180 rpm) at 28 °C for 3–4 days. These cultures (5 mL) were used to inoculate 30 mL of OM, HM, SFM, R5, NL200, NL300, NL400, NL410 medium (prepared without agar according to the recipes stated above), GUBC medium (10 g saccharose, 5 g meat extract, 5 g casamino acids, 5 g glycerol, 5 mL 1 M Na_2_HPO_4_/KH_2_PO_4_ pH 7.3, 2 mL Hunter's base dissolved in 1 L distilled water, pH adjusted to 7.3, and 10 mL Balch's vitamins added after autoclaving. Hunter's concentrated base: 20 g nitrilotriacetic acid, 14 g KOH, 59.3 g MgSO_4_·7H_2_O, 6.67 g CaCl_2_·2H_2_O, 0.0185 g (NH_4_)_6_Mo_7_O_24_·4H_2_O, 0.198 g FeSO_4_·7H_2_O, 0.25 g EDTA, 1.095 g ZnSO_4_·7H_2_O, 0.5 g FeSO_4_·7H_2_O, 0.154 g MnSO_4_·H_2_O, 0.0392 g CuSO_4_·5H_2_O, 0.025 g Co(NO_3_)_2_·7H_2_O, 0.0177 g Na_2_B_4_O_7_·10H_2_O dissolved in 1 L distilled water, pH adjusted to 6.8. Balch's vitamins: 5 mg *p*-aminobenzoic acid, 2 mg folic acid, 2 mg biotin, 5 mg nicotinic acid, 5 mg calcium pantothenate, 5 mg riboflavin, 5 mg thiamine HCl, 10 mg pyridoxine HCl (B6), 100 µg cyanocobalamin (B12), 5 mg thioctic acid (lipoic acid) dissolved in 1 L distilled water, pH adjusted to 7.0, sterilised by filtration), and ISP4 medium (10 g soluble starch, 2 g CaCO_3_, 1 g K_2_HPO_4_, 1 g MgCl_2_, 1 g NaCl, 2 g (NH_4_)SO_4_ and 1 mL trace metal mix 2 dissolved in 1 L distilled water, pH adjusted to 7.3. Trace metal mix 2 : 1 g FeSO_4_·7H_2_O, 1 g MnCl_2_·2H_2_O, 1 g ZnSO_4_·7H_2_O dissolved in 1 L distilled water) in 100 mL baffled flasks. After 7 days, cultures were harvested by centrifugation at 4000 rcf and 4 °C for 15 min. Supernatants were applied to C18 solid phase extraction (SPE) cartridges (1 g), and the aqueous flow-through was collected. Aqueous phases were diluted with methanol to an 80% final methanol concentration, filtered, and dried using a rotary evaporator. ^31^P NMR spectra were recorded in 20% D_2_O at 243 MHz on a Bruker Avance III HDX 600 MHz spectrometer fitted with a 5 mm Prodigy BBO H&F CryoProbe. Phosphorus chemical shifts are reported in ppm relative to a 10 mM l-phosphinothricin HCl reference (*δ*_P_ 51.2) using a double-chamber coaxial NMR tube. NMR data were analyzed using MestReNova 14.3.0.

## Author contributions

YM and CCH conceived the study. AZ, SNX, JM, JPGE, JB, CCH and YM designed the research and developed the methodology. AZ, SNX, JM, JPGE, JK and MI conducted experiments. All authors carried out the investigation. AZ, SNX, JM, JPGE curated the data. AZ, SNX and JPGE prepared the visualizations. AZ, JPGE and YM drafted the initial manuscript. JPGE, JB, UN, IN, WWM, CCH and YM supervised the work. WWM gave scientific advice. All authors reviewed, edited and approved the final version of the manuscript.

## Conflicts of interest

There are no conflicts to declare.

## Supplementary Material

CB-OLF-D5CB00254K-s001

## Data Availability

The genome accession numbers for all strains analyzed in this study are listed in the supplementary information (SI) (Table S1). Genome assemblies were obtained from NCBI. Strains are available from the DSMZ repository, with all strain-related metadata accessible through the BacDive metadatabase. Genome sequences were analyzed for biosynthetic gene clusters using antiSMASH (v6.0–7.1.0). Gene cluster families were generated using BiG-SCAPE (v2.0) and visualized with Cytoscape (v3.10.3); the clustering parameters and cutoff values are provided in the Methods section. The sequence of the *Kitasatospora fiedleri* DSM 114396^T^ genome, including the annotated phosphonate biosynthetic gene cluster (P-BGC), is available at NCBI under accession number GCA_948472415. *Kitasatospora fiedleri* phosphonate BGC description has been submitted to MIBiG (mibig.secondarymetabolites.org) and will be accessible upon the next database release with accession BGC0003187. All other supporting data, including raw ^31^P NMR spectra, gene synteny alignments, and experimental protocols, are provided in the SI. See DOI: https://doi.org/10.1039/d5cb00254k. Plasmids and strains generated in this study are available from the corresponding author upon request.

## References

[cit1] Miethke M., Pieroni M., Weber T., Brönstrup M., Hammann P., Halby L. (2021). *et al.*, Towards the sustainable discovery and development of new antibiotics. Nat. Rev. Chem..

[cit2] Bérdy J. (2012). Thoughts and facts about antibiotics: Where we are now and where we are heading. J. Antibiot..

[cit3] Hemmerling F., Piel J. (2022). Strategies to access biosynthetic novelty in bacterial genomes for drug discovery. Nat. Rev. Drug Discovery.

[cit4] Berdy B., Spoering A. L., Ling L. L., Epstein S. S. (2017). In situ cultivation of previously uncultivable microorganisms using the ichip. Nat. Protoc..

[cit5] Handayani I., Saad H., Ratnakomala S., Lisdiyanti P., Kusharyoto W., Krause J. (2021). *et al.*, Mining Indonesian Microbial Biodiversity for Novel Natural Compounds by a Combined Genome Mining and Molecular Networking Approach. Mar. Drugs.

[cit6] Nouioui I., Boldt J., Zimmermann A., Makitrynskyy R., Pötter G., Jando M. (2024). *et al.*, Biotechnological and pharmaceutical potential of twenty-eight novel type strains of *Actinomycetes* from different environments worldwide. Curr. Res. Microb. Sci..

[cit7] Crüsemann M., O’Neill E. C., Larson C. B., Melnik A. V., Floros D. J., da Silva R. R. (2017). *et al.*, Prioritizing Natural Product Diversity in a Collection of 146 Bacterial Strains Based on Growth and Extraction Protocols. J. Nat. Prod..

[cit8] Seidel V. , in Initial and Bulk Extraction of Natural Products Isolation, ed S. D. Sarker, L. Nahar, Natural Products Isolation, Humana Press, Totowa, NJ, 2012, p. 27–41 10.1007/978-1-61779-624-1_2 22367892

[cit9] Sarker S. D. , Latif Z. and Gray A. I., in Natural Product Isolation, ed S. D. Sarker, Z. Latif, A. I. Gray, Natural Products Isolation, Humana Press, Totowa, NJ, 2005, p. 1–25

[cit10] Katz L., Baltz R. H. (2016). Natural product discovery: past, present, and future. J. Ind. Microbiol. Biotechnol..

[cit11] Panter F D., Bader C., Müller R. (2021). Synergizing the potential of bacterial genomics and metabolomics to find novel antibiotics. Chem. Sci..

[cit12] Demain A. L. (2014). Importance of microbial natural products and the need to revitalize their discovery. J. Ind. Microbiol. Biotechnol..

[cit13] Baltz R. H. (2007). Antimicrobials from actinomycetes: back to the future. Microbe.

[cit14] Alam K., Mazumder A., Sikdar S., Zhao Y. M., Hao J., Song C. (2022). *et al.*, *Streptomyces*: The biofactory of secondary metabolites. Front. Microbiol..

[cit15] Medema M. H., Cimermancic P., Sali A., Takano E., Fischbach M. A. (2014). A Systematic Computational Analysis of Biosynthetic Gene Cluster Evolution: Lessons for Engineering Biosynthesis. PLoS Comput. Biol..

[cit16] Rudd B. A. M., Hopwood D. A. (1979). Genetics of Actinorhodin Biosynthesis by *Streptomyces coelicolor* A3(2). Microbiology.

[cit17] Nett M., Ikeda H., Moore B. S. (2009). Genomic basis for natural product biosynthetic diversity in the actinomycetes. Nat. Prod. Rep..

[cit18] Gavriilidou A., Kautsar S. A., Zaburannyi N., Krug D., Müller R., Medema M. H. (2022). *et al.*, Compendium of specialized metabolite biosynthetic diversity encoded in bacterial genomes. Nat. Microbiol..

[cit19] Zerikly M., Challis G. L. (2009). Strategies for the Discovery of New Natural Products by Genome Mining. ChemBioChem.

[cit20] Fischbach M. A., Walsh C. T. (2006). Assembly-Line Enzymology for Polyketide and Nonribosomal Peptide Antibiotics:  Logic, Machinery, and Mechanisms. Chem. Rev..

[cit21] Machado H., Tuttle R. N., Jensen P. R. (2017). Omics-based natural product discovery and the lexicon of genome mining. Curr. Opin. Microbiol..

[cit22] Ziemert N., Alanjary M., Weber T. (2016). The evolution of genome mining in microbes–a review. Nat. Prod. Rep..

[cit23] Okuhara M., Kuroda Y., Goto T., Okamoto M., Terano H., Kohsaka M. (1980). *et al.*, Studies on new phosphonic acid antibiotics studies on new phosphonic acid antibiotics. J. Antibiot..

[cit24] Eliot A. C., Griffin B. M., Thomas P. M., Johannes T. W., Kelleher N. L., Zhao H. (2008). *et al.*, Cloning, Expression, and Biochemical Characterization of *Streptomyces rubellomurinus* Genes Required for Biosynthesis of Antimalarial Compound FR900098. Chem. Biol..

[cit25] Takahashi S., Kuzuyama T., Watanabe H., Seto H. (1998). A 1-deoxy-d-xylulose 5-phosphate reductoisomerase catalyzing the formation of 2-C-methyl-d-erythritol 4-phosphate in an alternative nonmevalonate pathway for terpenoid biosynthesis. Proc. Natl. Acad. Sci. U. S. A..

[cit26] Rohmer M., Rohmer M. (1999). The discovery of a mevalonate-independent pathway for isoprenoid biosynthesis in bacteria, algae and higher plants. Nat. Prod. Rep..

[cit27] Stapley E. O., Hendlin D., Mata J. M., Jackson M., Wallick H., Hernandez S. (1969). *et al.*, Phosphonomycin. I. Discovery and *in vitro* biological characterization. Antimicrob. Agents Chemother..

[cit28] Hidaka T., Goda M., Kuzuyama T., Takei N., Hidaka M., Seto H. (1995). Cloning and nucleotide sequence of fosfomycin biosynthetic genes of *Streptomyces wedmorensis*. Mol. Gen. Genet. MGG..

[cit29] Woodyer R. D., Shao Z., Thomas P. M., Kelleher N. L., Blodgett J. A. V., Metcalf W. W., van der Donk W. A., Zhao H. (2006). Heterologous Production of Fosfomycin and Identification of the Minimal Biosynthetic Gene Cluster. Chem. Biol..

[cit30] Kim S. Y., Ju K. S., Metcalf W. W., Evans B. S., Kuzuyama T., van der Donk W. A. (2012). Different biosynthetic pathways to fosfomycin in *Pseudomonas syringae* and *Streptomyces* species. Antimicrob. Agents Chemother..

[cit31] Shoji J., Kato T., Hinoo H., Hattori T., Hirooka K., Matsumoto K. (1986). *et al.*, Production of fosfomycin (phosphonomycin) by *Pseudomonas syringae*. J. Antibiot..

[cit32] Falagas M. E., Giannopoulou K. P., Kokolakis G. N., Rafailidis P. I. (2008). Fosfomycin: Use Beyond Urinary Tract and Gastrointestinal Infections. Clin. Infect. Dis..

[cit33] Kahan F. M., Kahan J. S., Cassidy P. J., Kropp H. (1974). The Mechanism of Action of Fosfomycin (phosphonomycin). Ann. N. Y. Acad. Sci..

[cit34] Petek M., Baebler Š., Kuzman D., Rotter A., Podlesek Z., Gruden K. (2010). *et al.*, Revealing fosfomycin primary effect on *Staphylococcus aureus* transcriptome: modulation of cell envelope biosynthesis and phosphoenolpyruvate induced starvation. BMC Microbiol..

[cit35] Eschenburg S., Priestman M., Schönbrunn E. (2005). Evidence That the Fosfomycin Target Cys115 in UDP-*N*-acetylglucosamine Enolpyruvyl Transferase (MurA) Is Essential for Product Release. J. Biol. Chem..

[cit36] Bayer E., Gugel K. H., Hägele K., Hagenmaier H., Jessipow S., König W. A. (1972). *et al.*, Stoffwechselprodukte von Mikroorganismen. 98. Mitteilung. Phosphinothricin und Phosphinothricyl-Alanyl-Alanin. Helv. Chim. Acta.

[cit37] Schwartz D., Grammel N., Heinzelmann E., Keller U., Wohlleben W. (2005). Phosphinothricin tripeptide synthetases in *Streptomyces viridochromogenes* Tü494. Antimicrob. Agents Chemother..

[cit38] Schwartz D., Berger S., Heinzelmann E., Muschko K., Welzel K., Wohlleben W. (2004). Biosynthetic Gene Cluster of the Herbicide Phosphinothricin Tripeptide from *Streptomyces viridochromogenes* Tü494. Appl. Environ. Microbiol..

[cit39] Wendler C., Putzer A., Wild A. (1992). Effect of Glufosinate (Phosphinothricin) and Inhibitors of Photorespiration on Photosynthesis and Ribulose-1,5-Bisphosphate Carboxylase Activity. J. Plant Physiol..

[cit40] Ju K. S., Doroghazi J. R., Metcalf W. W. (2014). Genomics-Enabled Discovery of Phosphonate Natural Products and their Biosynthetic Pathways. J. Ind. Microbiol. Biotechnol..

[cit41] Falagas M. E., Vouloumanou E. K., Samonis G., Vardakas K. Z. Fosfomycin (2016). Clin. Microbiol. Rev..

[cit42] Thompson C. J. and Seto H., in Chapter 6 - Bialaphos, ed Vining L. C., Stuttard C., Genetics and Biochemistry of Antibiotic Production, Butterworth-Heinemann, Boston, 1995, p. 197–222

[cit43] Widler L., Jahnke W., Green R. J. (2012). The Chemistry of Bisphosphonates: From Antiscaling Agents to Clinical Therapeutics. Adv. Anticancer Agents Med. Chem..

[cit44] Metcalf W. W., van der Donk W. A. (2009). Biosynthesis of Phosphonic and Phosphinic Acid Natural Products. Annu. Rev. Biochem..

[cit45] Horsman G. P., Zechel D. L. (2017). Phosphonate Biochemistry. Chem. Rev..

[cit46] Ntai I., Phelan V. V., Bachmann B. O. (2006). Phosphonopeptide K-26 biosynthetic intermediates in *Astrosporangium hypotensionis*. Chem. Commun..

[cit47] Ntai I., Manier M. L., Hachey D. L., Bachmann B. O. (2005). Biosynthetic Origins of C−P Bond Containing Tripeptide K-26. Org. Lett..

[cit48] Elise B., Michael M., Barry R. J. (1988). Dunaway-Mariano Debra. Catalysis and thermodynamics of the phosphoenolpyruvate/phosphonopyruvate rearrangement. Entry into the phosphonate class of naturally occurring organophosphorus compounds. J. Am. Chem. Soc..

[cit49] Kayrouz C. M., Zhang Y., Pham T. M., Ju K. S. (2020). Genome Mining Reveals the Phosphonoalamide Natural Products and a New Route in Phosphonic Acid Biosynthesis. ACS Chem. Biol..

[cit50] Villarreal-Chiu J. F., Quinn J. P., McGrath J. W. (2012). The genes and enzymes of phosphonate metabolism by bacteria, and their distribution in the marine environment. Front. Microbiol..

[cit51] Yu X., Doroghazi J. R., Janga S. C., Zhang J. K., Circello B., Griffin B. M. (2013). *et al.*, Diversity and abundance of phosphonate biosynthetic genes in nature. Proc. Natl. Acad. Sci. U. S. A..

[cit52] Chen C. C. H., Han Y., Niu W., Kulakova A. N., Howard A., Quinn J. P. (2006). *et al.*, Structure and kinetics of phosphonopyruvate hydrolase from *Variovorax* sp. Pal2: new insight into the divergence of catalysis within the PEP mutase/isocitrate lyase superfamily. Biochemistry.

[cit53] Blin K., Shaw S., Augustijn H. E., Reitz Z. L., Biermann F., Alanjary M. (2023). *et al.*, AntiSMASH 7.0: new and improved predictions for detection, regulation, chemical structures and visualisation. Nucleic Acids Res..

[cit54] Heinzelmann E., Kienzlen G., Kaspar S., Recktenwald J., Wohlleben W., Schwartz D. (2001). The phosphinomethylmalate isomerase gene pmi, encoding an aconitase-like enzyme, is involved in the synthesis of phosphinothricin tripeptide in *Streptomyces viridochromogenes*. Appl. Environ. Microbiol..

[cit55] Ju K. S., Gao J., Doroghazi J. R., Wang K. K. A., Thibodeaux C. J., Li S. (2015). *et al.*, Discovery of phosphonic acid natural products by mining the genomes of 10 000 actinomycetes. Proc. Natl. Acad. Sci. U. S. A..

[cit56] Zhang Y., Chen L., Wilson J. A., Cui J., Roodhouse H., Kayrouz C. (2022). *et al.*, Valinophos Reveals a New Route in Microbial Phosphonate Biosynthesis That Is Broadly Conserved in Nature. J. Am. Chem. Soc..

[cit57] Zhang Y., Pham T. M., Kayrouz C., Ju K. S. (2022). Biosynthesis of Argolaphos Illuminates the Unusual Biochemical Origins of Aminomethylphosphonate and Nε-Hydroxyarginine Containing Natural Products. J. Am. Chem. Soc..

[cit58] Evans B. S., Zhao C., Gao J., Evans C. M., Ju K. S., Doroghazi J. R. (2013). *et al.*, Discovery of the Antibiotic Phosacetamycin via a New Mass Spectrometry-Based Method for Phosphonic Acid Detection. ACS Chem. Biol..

[cit59] Polidore A. L. A., Furiassi L., Hergenrother P. J., Metcalf W. W. (2021). A Phosphonate Natural Product Made by *Pantoea ananatis* is Necessary and Sufficient for the Hallmark Lesions of Onion Center Rot. mBio.

[cit60] Cui J. J., Zhang Y., Ju K. S. (2024). Phosphonoalamides Reveal the Biosynthetic Origin of Phosphonoalanine Natural Products and a Convergent Pathway for Their Diversification. Angew. Chem., Int. Ed..

[cit61] Wilson J., Cui J., Nakao T., Kwok H., Zhang Y., Kayrouz C. (2023). *et al.*, Discovery of Antimicrobial Phosphonopeptide Natural Products from *Bacillus velezensis* by Genome Mining. Appl. Environ. Microbiol..

[cit62] Nouioui I., Zimmermann A., Hennrich O., Xia S., Rössler O., Makitrynskyy R. (2024). *et al.*, Challenging old microbiological treasures for natural compound biosynthesis capacity. Front. Bioeng. Biotechnol..

[cit63] Cui J., Ju K. S. (2024). Biosynthesis of *Bacillus* Phosphonoalamides Reveals Highly Specific Amino Acid Ligation. ACS Chem. Biol..

[cit64] Polidore A. L. A., Caserio A. D., Zhu L., Metcalf W. W. (2023). Complete Biochemical Characterization of Pantaphos Biosynthesis Highlights an Unusual Role for a SAM-Dependent Methyltransferase. Angew. Chem., Int. Ed..

[cit65] Freestone T. S., Ju K. S., Wang B., Zhao H. (2017). Discovery of a Phosphonoacetic Acid Derived Natural Product by Pathway Refactoring. ACS Synth. Biol..

[cit66] Chu L., Luo X., Zhu T., Cao Y., Zhang L., Deng Z. (2022). *et al.*, Harnessing phosphonate antibiotics argolaphos biosynthesis enables a synthetic biology-based green synthesis of glyphosate. Nat. Commun..

[cit67] Bown L., Hirota R., Goettge M. N., Cui J., Krist D. T., Zhu L. (2023). *et al.*, A Novel Pathway for Biosynthesis of the Herbicidal Phosphonate Natural Product Phosphonothrixin Is Widespread in Actinobacteria. J. Bacteriol..

[cit68] Takahashi E., Kimura T., Nakamura K., Arahira M., Iida M. (1995). Phosphonothrixin, a Novel Herbicidal Antibiotic Produced by *Saccharothrix* sp. ST-888 I. Taxonomy, Fermentation, Isolation and Biological Properties. J Antibiot (Tokyo).

[cit69] Cioni J. P., Doroghazi J. R., Ju K. S., Yu X., Evans B. S., Lee J. (2014). *et al.*, Cyanohydrin Phosphonate Natural Product from *Streptomyces regensis*. J. Nat. Prod..

[cit70] Navarro-Muñoz J. C., Selem-Mojica N., Mullowney M. W., Kautsar S. A., Tryon J. H., Parkinson E. I. (2020). *et al.*, A computational framework to explore large-scale biosynthetic diversity. Nat. Chem. Biol..

[cit71] Hendlin D., Stapley E. O., Jackson M., Wallick H., Miller A. K., Wolf F. J. (1969). *et al.*, Phosphonomycin, a New Antibiotic Produced by Strains of *Streptomyces*. Science.

[cit72] Stasi R., Neves H. I., Spira B. (2019). Phosphate uptake by the phosphonate transport system PhnCDE. BMC Microbiol..

[cit73] Gomez-Escribano J. P., Zimmermann A., Xia S. N., Döppner M., Moschny J., Hughes C. C. (2025). *et al.*, Application of a replicative targetable vector system for difficult-to-manipulate streptomycetes. Appl. Microbiol. Biotechnol..

[cit74] Zimmermann A., Nouioui I., Pötter G., Neumann-Schaal M., Wolf J., Wibberg D. (2023). *et al.*, *Kitasatospora fiedleri* sp. nov., a novel antibiotic-producing member of the genus *Kitasatospora*. Int. J. Syst. Evol. Microbiol..

[cit75] Gilchrist C. L. M., Chooi Y. H. (2021). Clinker & clustermap.js: automatic generation of gene cluster comparison figures. Bioinformatics.

[cit76] Agarwal V., Peck S. C., Chen J. H., Borisova S. A., Chekan J. R., van der Donk W. A. (2014). *et al.*, Structure and function of phosphonoacetaldehyde dehydrogenase: the missing link in phosphonoacetate formation. Chem. Biol..

[cit77] Badri A., Williams A., Xia K., Linhardt R. J., Koffas M. A. G. (2019). Increased 3′-Phosphoadenosine-5′-phosphosulfate Levels in Engineered *Escherichia coli* Cell Lysate Facilitate the In Vitro Synthesis of Chondroitin Sulfate A. Biotechnol. J..

[cit78] Zhao Q., He Q., Ding W., Tang M., Kang Q., Yu Y. (2008). *et al.*, Characterization of the Azinomycin B Biosynthetic Gene Cluster Revealing a Different Iterative Type I Polyketide Synthase for Naphthoate Biosynthesis. Chem. Biol..

[cit79] Mao Y., Varoglu M., Sherman D. H. (1999). Molecular characterization and analysis of the biosynthetic gene cluster for the antitumor antibiotic mitomycin C from *Streptomyces lavendulae* NRRL 2564. Chem. Biol..

[cit80] Schinko E., Schad K., Eys S., Keller U., Wohlleben W. (2009). Phosphinothricin-tripeptide biosynthesis: An original version of bacterial secondary metabolism?. Phytochemistry.

[cit81] Blin K., Shaw S., Kloosterman A. M., Charlop-Powers Z., van Wezel G. P., Medema M. H. (2021). *et al.*, antiSMASH 6.0: improving cluster detection and comparison capabilities. Nucleic Acids Res..

[cit82] Kuraku S., Zmasek C. M., Nishimura O., Katoh K. (2013). aLeaves facilitates on-demand exploration of metazoan gene family trees on MAFFT sequence alignment server with enhanced interactivity. Nucleic Acids Res..

[cit83] Katoh K., Rozewicki J., Yamada K. D. (2019). MAFFT online service: multiple sequence alignment, interactive sequence choice and visualization. Brief Bioinform..

[cit84] Edler D., Klein J., Antonelli A., Silvestro D. (2021). raxmlGUI 2.0: A graphical interface and toolkit for phylogenetic analyses using RAxML. Methods Ecol. Evol..

[cit85] Darriba D., Posada D., Kozlov A. M., Stamatakis A., Morel B., Flouri T. (2020). ModelTest-NG: A New and Scalable Tool for the Selection of DNA and Protein Evolutionary Models. Mol. Biol. Evol..

[cit86] Sigle S., Steblau N., Wohlleben W., Muth G. (2016). Polydiglycosylphosphate Transferase PdtA (SCO2578) of *Streptomyces coelicolor* A3(2) Is Crucial for Proper Sporulation and Apical Tip Extension under Stress Conditions. Appl. Environ. Microbiol..

[cit87] Paget M. S. B., Chamberlin L., Atrih A., Foster S. J., Buttner M. J. (1999). Evidence that the Extracytoplasmic Function Sigma Factor ςE Is Required for Normal Cell Wall Structure in *Streptomyces coelicolor* A3(2). J. Bacteriol..

[cit88] MacNeil D. J., Gewain K. M., Ruby C. L., Dezeny G., Gibbons P. H., MacNeil T. (1992). Analysis of *Streptomyces avermitilis* genes required for avermectin biosynthesis utilizing a novel integration vector. Gene.

[cit89] Sambrook J. , Fritsch E. F. and Maniatis T., Molecular cloning: a laboratory manual. Cold Spring Harbor Laboratory, Cold Spring Harbor, N.Y., 1989

[cit90] Kieser T. , Bibb M. J., Buttner M. J., Chater K. F. and Hopwood D. A., Practical Streptomyces Genetics. ed, Kieser T., Bibb M. J., Buttner M. J., Chater K. F., Hopwood D. A., John Innes Foundation, John Innes Centre, Norwich Research Park, Colney, Norwich NR4 7UH, England, 2000. p. 613

[cit91] Kozlov A. M., Darriba D., Flouri T., Morel B., Stamatakis A. (2019). RAxML-NG: a fast, scalable and user-friendly tool for maximum likelihood phylogenetic inference. Bioinformatics..

