# Supplementary material for: Expanding the actinomycetes landscape for phosphonate natural products through genome mining
Source: RSC Chem Biol. 2025 Dec 16;7(2):298–312. doi: 10.1039/d5cb00254k (PMC12723261; doi:10.1039/d5cb00254k)
Supplement: CB-007-D5CB00254K-s001 [file CB-007-D5CB00254K-s001.pdf]

Supplementary Information (SI) for RSC Chemical Biology.
This journal is © The Royal Society of Chemistry 2025

Supporting Information to: "Expanding the Actinomycetes Landscape for Phosphonate Natural Products through Genome Mining"

Alina Zimmermann[1,2], *Shu-Ning Xia*[2,3], *Julia Moschny*[2,3], *Juan Pablo Gomez-Escribano*[1], *Judith Boldt*[1,4], *Ulrich Nübel*[1,4,5,6], *Imen Nouioui*[1], *Janina Krause*[3], *Mattis Kreins Irle*[1], *William W. Metcalf*[7], *Chambers C. Hughes*[2,3,8], *Yvonne Mast*[1,2,5,6*]

[1]Leibniz Institute DSMZ -German Collection of Microorganisms and Cell Cultures, Inhoffenstraße 7B, 38124 Braunschweig, Germany

[2]German Center for Infection Research (DZIF), Partner Site Tübingen, 72076 Tübingen, Germany

[3]Department of Microbial Bioactive Compounds, Interfaculty Institute of Microbiology and Infection Medicine (IMIT), University of Tübingen, Auf der Morgenstelle 28, 72076 Tübingen, Germany

[4]German Center for Infection Research (DZIF), Partner Site Hannover-Braunschweig, 38124 Braunschweig, Germany

[5]Braunschweig Integrated Centre of Systems Biology (BRICS), Rebenring 56, 38106 Braunschweig, Germany

[6]Technische Universität Braunschweig, Institut für Mikrobiologie, Spielmannstr. 7, 38106 Braunschweig, Germany

[7]Carl R. Woese Institute for Genomic Biology, 1206 West Gregory Drive, Urbana, IL 61801, United States of America

[8]Cluster of Excellence EXC 2124: Controlling Microbes to Fight Infection, University of Tübingen, 72076 Tübingen, Germany

Table of Contents

Table of Contents 2

Figure S1. Gene Cluster Families of identified and known P-BGCs 4

Figure S2. ^{31}P NMR spectra of concentrated supernatant from *Kibdelosporangium banguiense* DSM 46670 and *Saccharopolyspora spinosa* DSM 44228 in different media..5

Figure S3. ^{31}P NMR spectra of concentrated supernatant from *Streptomyces aureocirculatus* DSM 40386 and *Streptomyces iranensis* DSM 41954 in different media 6

Figure S4. ^{31}P NMR spectra of concentrated supernatant from *Streptomyces mutomycini* DSM 41691 and *Kitasatospora cheerisanensis* DSM 101999 in different media 7

Figure S5. ^{31}P NMR spectra of concentrated supernatant from *Streptomyces glauciniger* DSM 41867 and *Streptomyces seoulensis* DSM 41840 in different media 8

Figure S6. ^{31}P NMR spectra of concentrated supernatant from *Kitasatospora fiedleri* DSM 114396 and *Streptomyces sp.* Tü H45 in different media 9

Figure S7. Synteny analysis for defining the boundaries of the putative P-BGC. 10

Figure S8. *clinker* analysis for defining the boundaries of the putative P-BGC. 12

Figure S9. Visualization of the putative P-BGC and GC-Frame plot in Artemis. 18

Figure S10. ^{31}P NMR spectra of concentrated supernatants from heterologous expression strains...................... 19

Figure S11. BLAST analysis of amino acid sequences from the *Kitasatospora fiedleri* P-BGC 20

Figure S12. Alignment of aldehyde dehydrogenase enzymes from P-BGCs.. 20

Figure S13. Overview of characterized enzyme functions in phosphonate biosynthetic pathways detected as predicted proteins encoded in *K. fiedleri* pBGC.. 22

Table S1. Identified potential phosphonate producer strains from the DSMZ and Tübingen collection and known producers of reduced phosphorus compounds included in the analysis 23

Table S2. Bioassay results in the test of putative phosphonate producers against *Escherichia coli* WM6242, *E. coli* K12 and *Kocuria rhizophila.* 37

Table S3. Plasmids used in this study... 38

Table S4. Strains used in this study 38

Table S5. Oligonucleotides used in this study 39

Table S6. Proposed phosphonate biosynthetic gene cluster from *Kitasatospora fiedleri* DSM 114396, based on the chromosome sequence with NCBI accession NZ_OX419519.1 41

Figure S1. Gene Cluster Families of identified and known P-BGCs

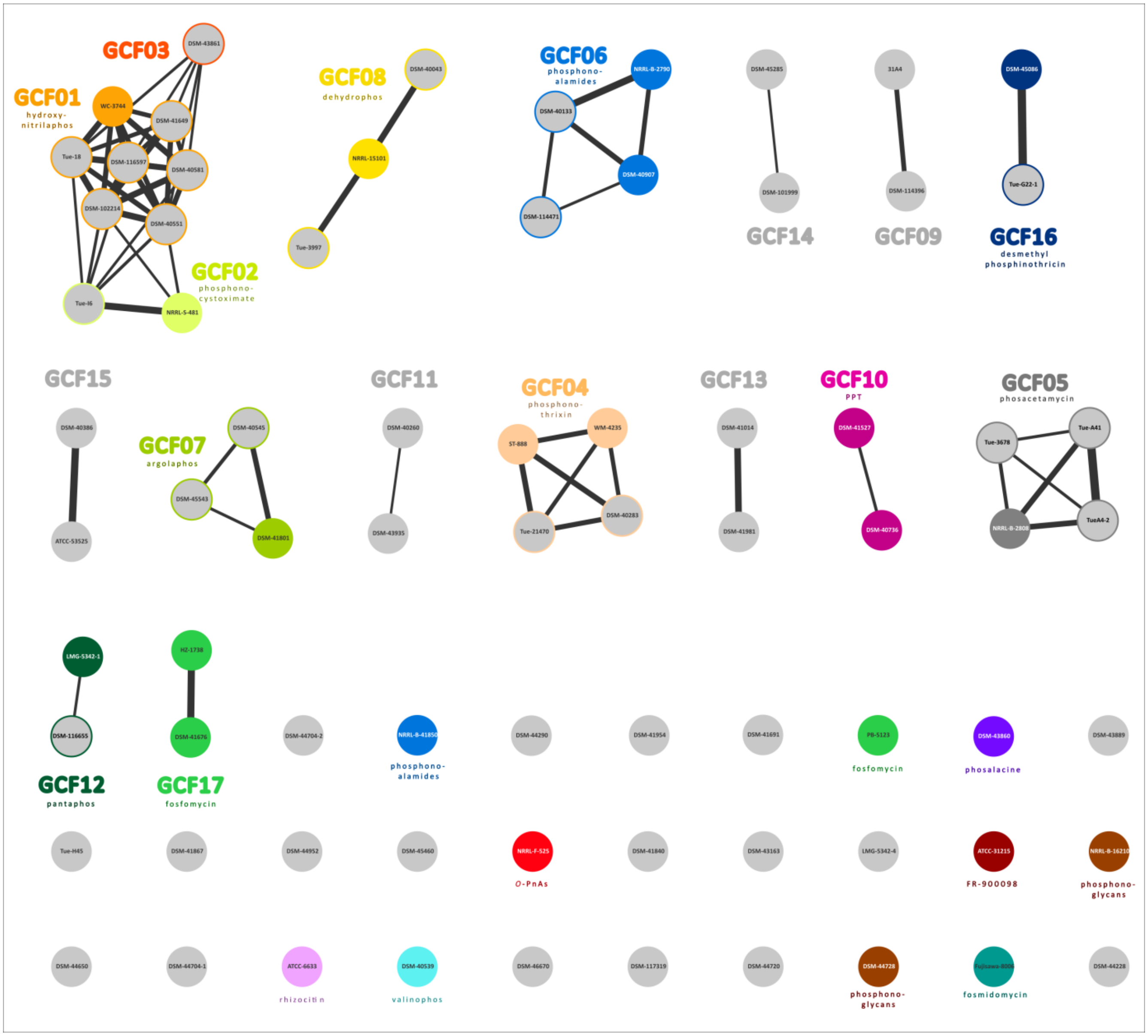

Figure S1. Gene cluster family (GCF) network as obtained from the BiG-SCAPE analysis and visualized with Cytoscape. Each node corresponds to a BGC. Colored spheres represent GCFs/singletons with sequences from previously identified phosphonate producers, light grey spheres are P-BGCs with unknown phosphonate product (for references see Table S1). Edges between two nodes represent a distance between the P-BGCs below a BiG-SCAPE cutoff threshold of 0.612 and distances are further represented by weighted lining of edges (lines get thicker with decreased distance).

Figure S2. ^{31}P NMR spectra of concentrated supernatant from *Kibdelosporangium banguiense* DSM 46670 and *Saccharopolyspora spinosa* DSM 44228 in different media

Baffled flasks containing OM medium, HM medium, SFM medium, R5 medium, GUBC medium, ISP4 medium, NL200 medium, NL300 medium, NL400 medium and NL410 medium were inoculated with starter culture. Cultures were incubated on a rotary shaker for 7 days. Culture supernatants were concentrated and analyzed by ^{31}P NMR spectroscopy. L-phosphinothricin HCl (10 mM in H_2O, δ_P 51.2 ppm) was used as a chemical shift reference. Signals with chemical shifts > 8 ppm are putative phosphonate-containing compounds.

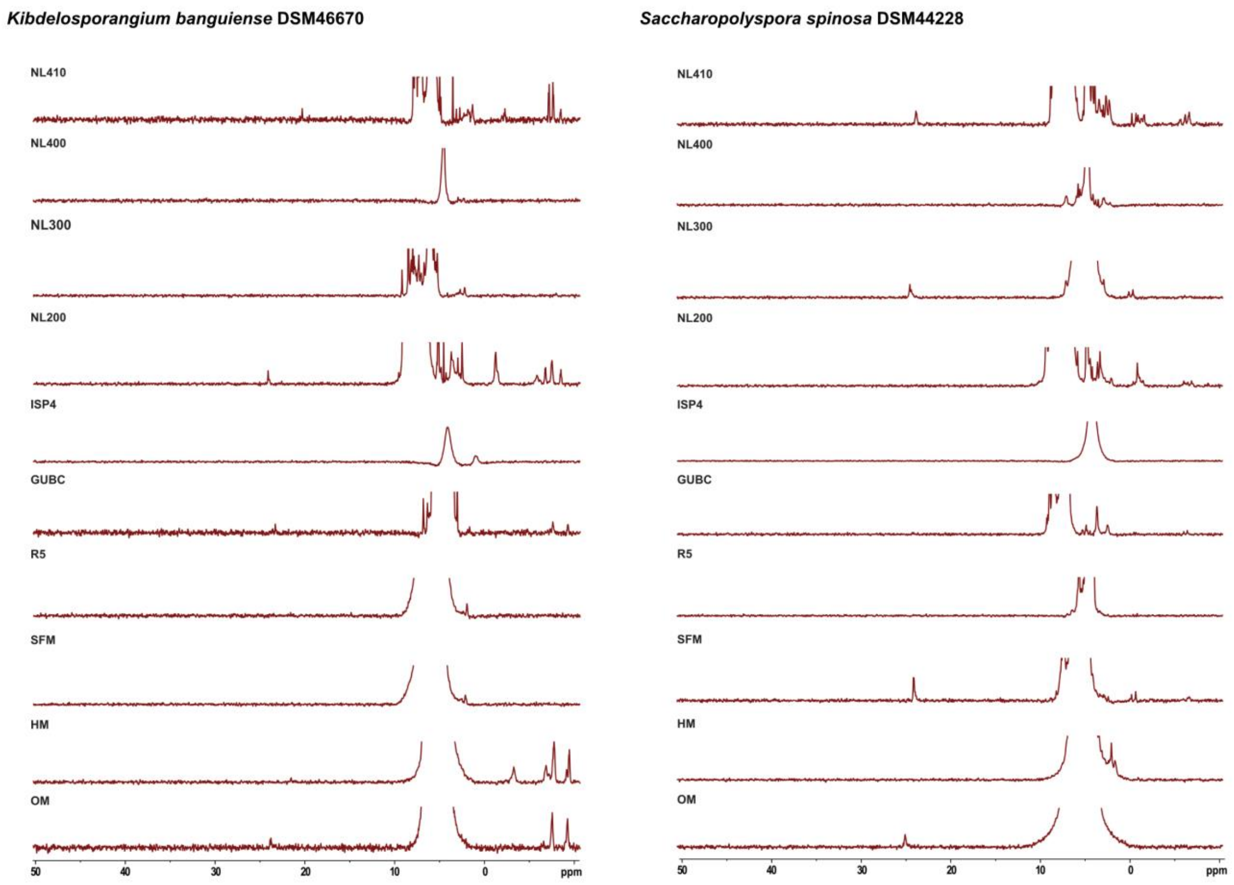

Figure S3. ^{31}P NMR spectra of concentrated supernatant from *Streptomyces aureocirculatus* DSM 40386 and *Streptomyces iranensis* DSM 41954 in different media

Baffled flasks containing OM medium, HM medium, SFM medium, R5 medium, GUBC medium, ISP4 medium, NL200 medium, NL300 medium, NL400 medium and NL410 medium were inoculated with starter culture. Cultures were incubated on a rotary shaker for 7 days. Culture supernatants were concentrated and analyzed by ^{31}P NMR spectroscopy. L-phosphinothricin HCl (10 mM in H_2O, δ_P 51.2 ppm) was used as a chemical shift reference. Signals with chemical shifts > 8 ppm are putative phosphonate-containing compounds.

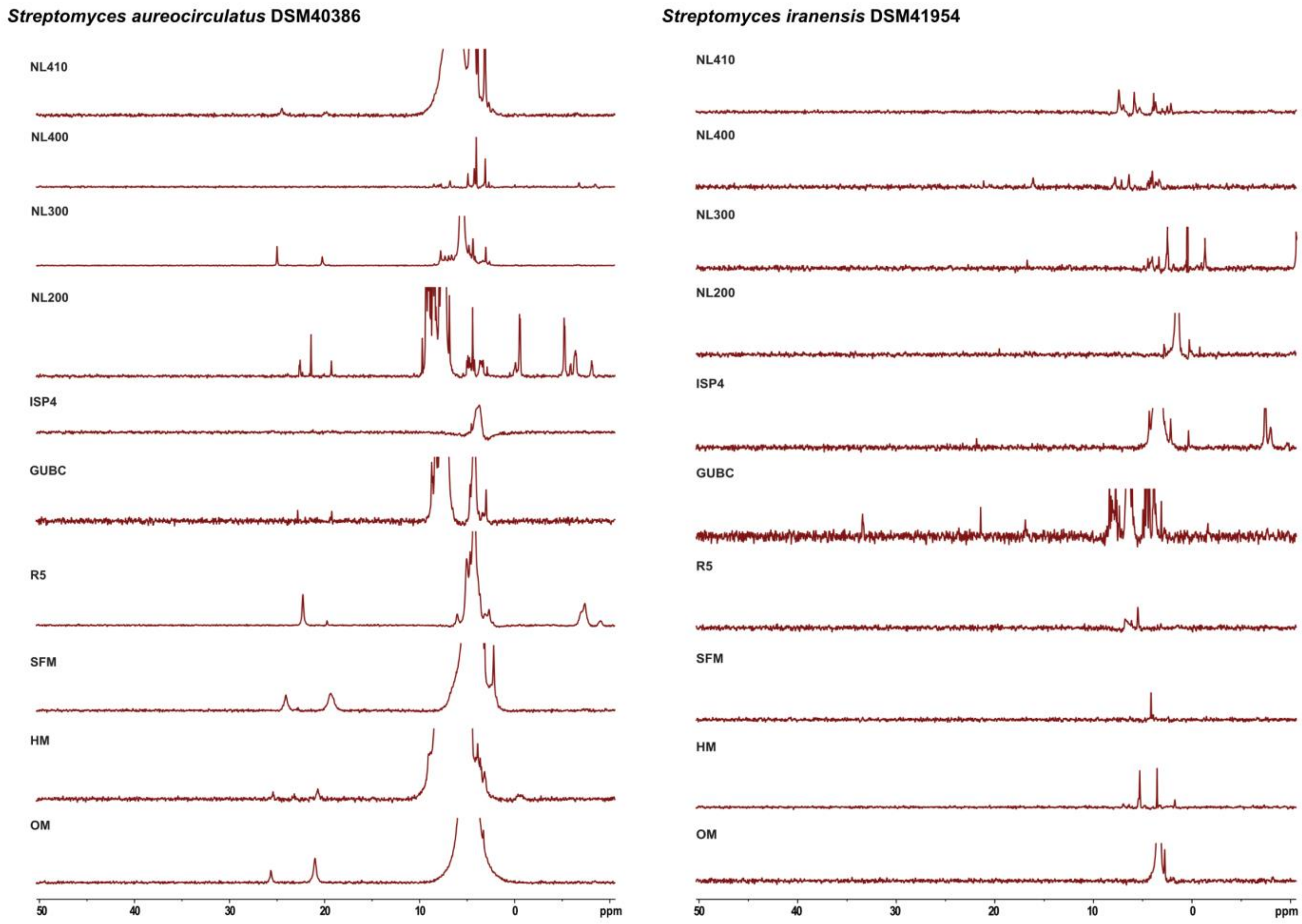

Figure S4. ^{31}P NMR spectra of concentrated supernatant from *Streptomyces mutomycini* DSM 41691 and *Kitasatospora cheerisanensis* DSM 101999 in different media

Baffled flasks containing OM medium, HM medium, SFM medium, R5 medium, GUBC medium, ISP4 medium, NL200 medium, NL300 medium, NL400 medium and NL410 medium were inoculated with starter culture. Cultures were incubated on a rotary shaker for 7 days. Culture supernatants were concentrated and analyzed by ^{31}P NMR spectroscopy. L-phosphinothricin HCl (10 mM in H_2O, δ_P 51.2 ppm) was used as a chemical shift reference. Signals with chemical shifts > 8 ppm are putative phosphonate-containing compounds.

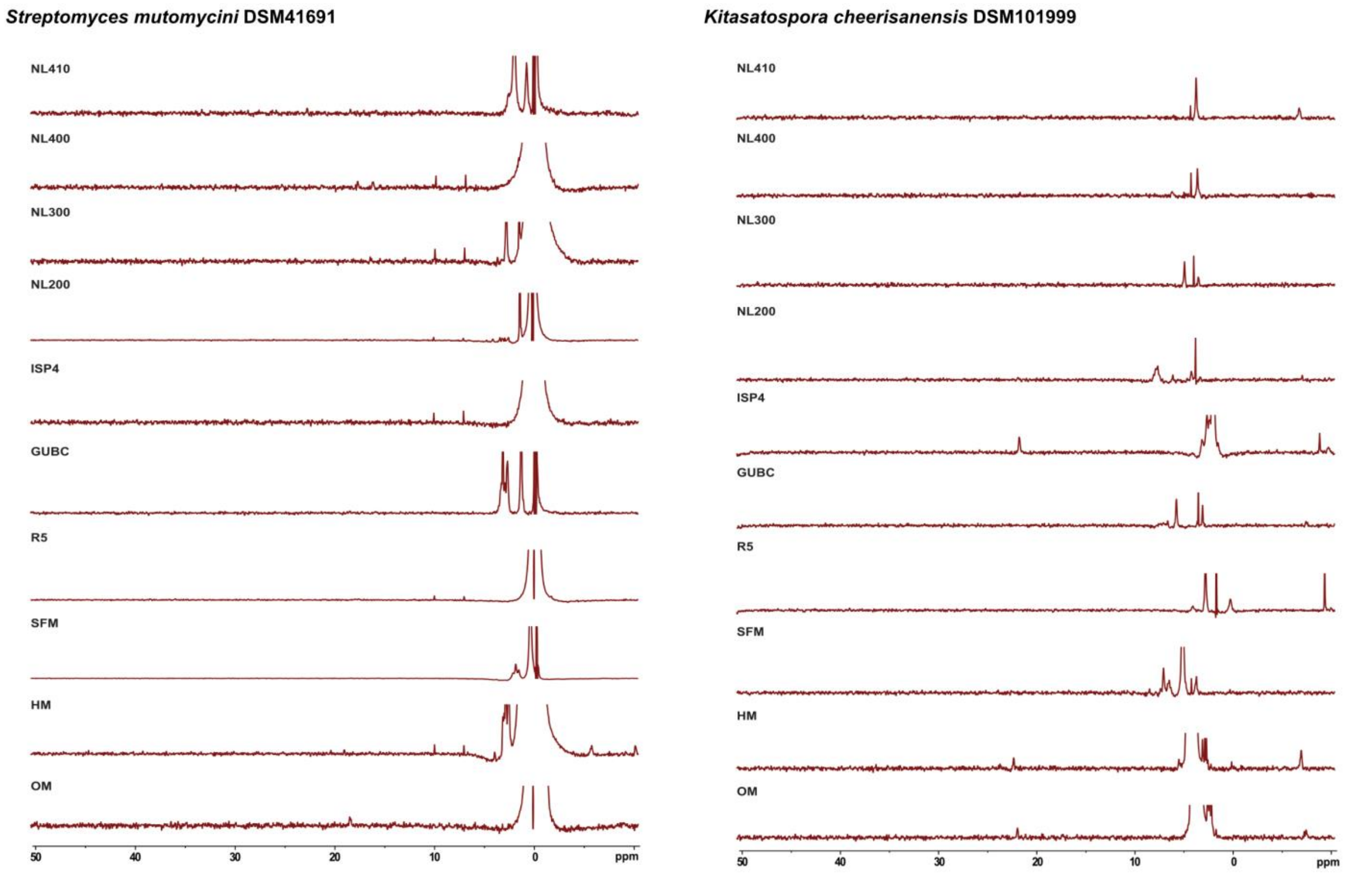

Figure S5. ^{31}P NMR spectra of concentrated supernatant from *Streptomyces glauciniger* DSM 41867 and *Streptomyces seoulensis* DSM 41840 in different media

Baffled flasks containing OM medium, HM medium, SFM medium, R5 medium, GUBC medium, ISP4 medium, NL200 medium, NL300 medium, NL400 medium and NL410 medium were inoculated with starter culture. Cultures were incubated on a rotary shaker for 7 days. Culture supernatants were concentrated and analyzed by ^{31}P NMR spectroscopy. L-phosphinothricin HCl (10 mM in H_2O, δ_P 51.2 ppm) was used as a chemical shift reference. Signals with chemical shifts > 8 ppm are putative phosphonate-containing compounds.

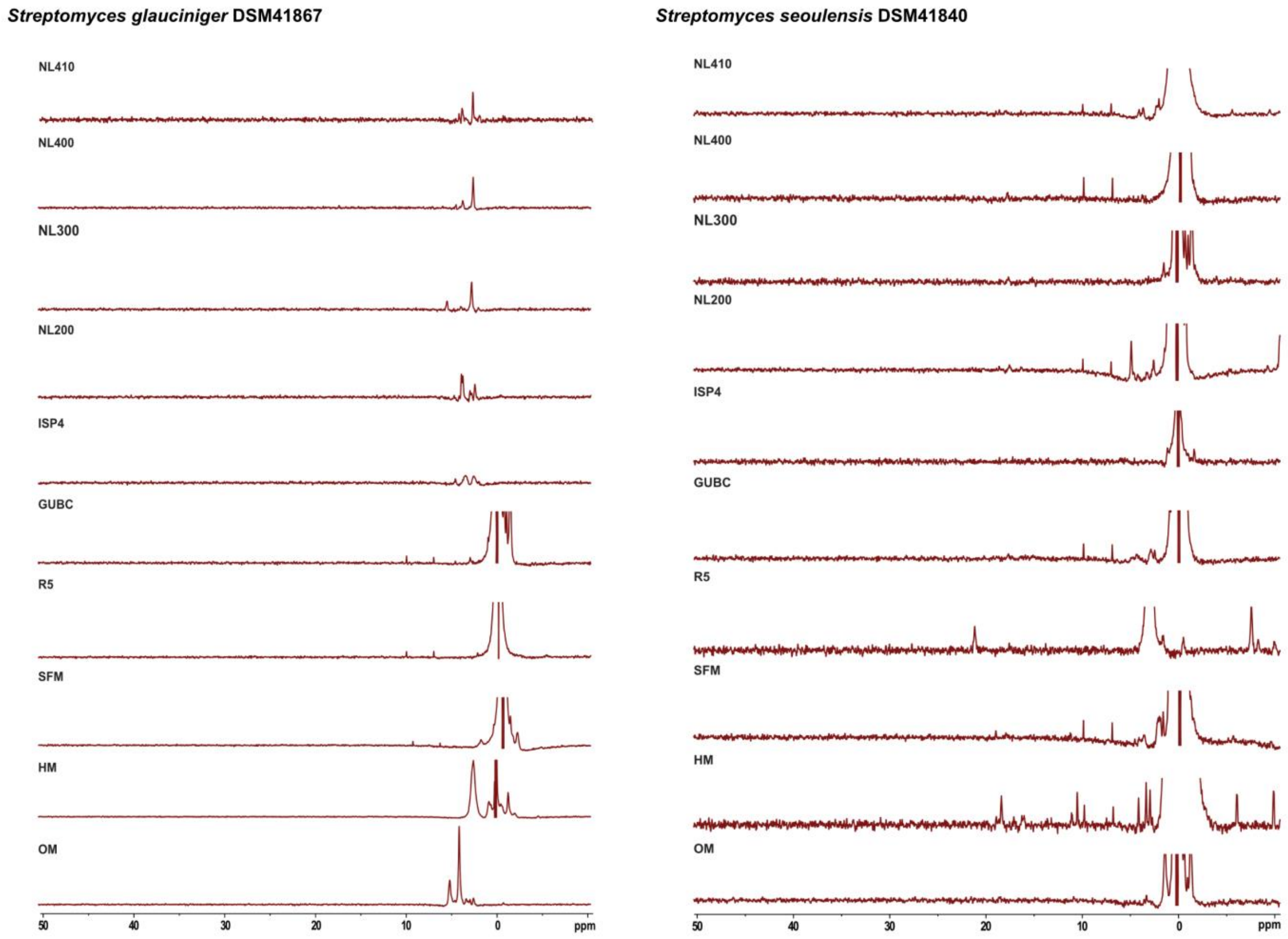

Figure S6. **^{31}P NMR spectra of concentrated supernatant from *Kitasatospora fiedleri* DSM 114396 and *Streptomyces sp.* Tü H45 in different media**

Baffled flasks containing OM medium, HM medium, SFM medium, R5 medium, GUBC medium and ISP4 medium were inoculated with starter culture. Cultures were incubated on a rotary shaker for 7 days. Culture supernatants were concentrated and analyzed by ^{31}P NMR spectroscopy. L-phosphinothricin HCl (10 mM in H_2O, δ_P 51.2 ppm) was used as a chemical shift reference. Signals with chemical shifts > 8 ppm are putative phosphonate-containing compounds.

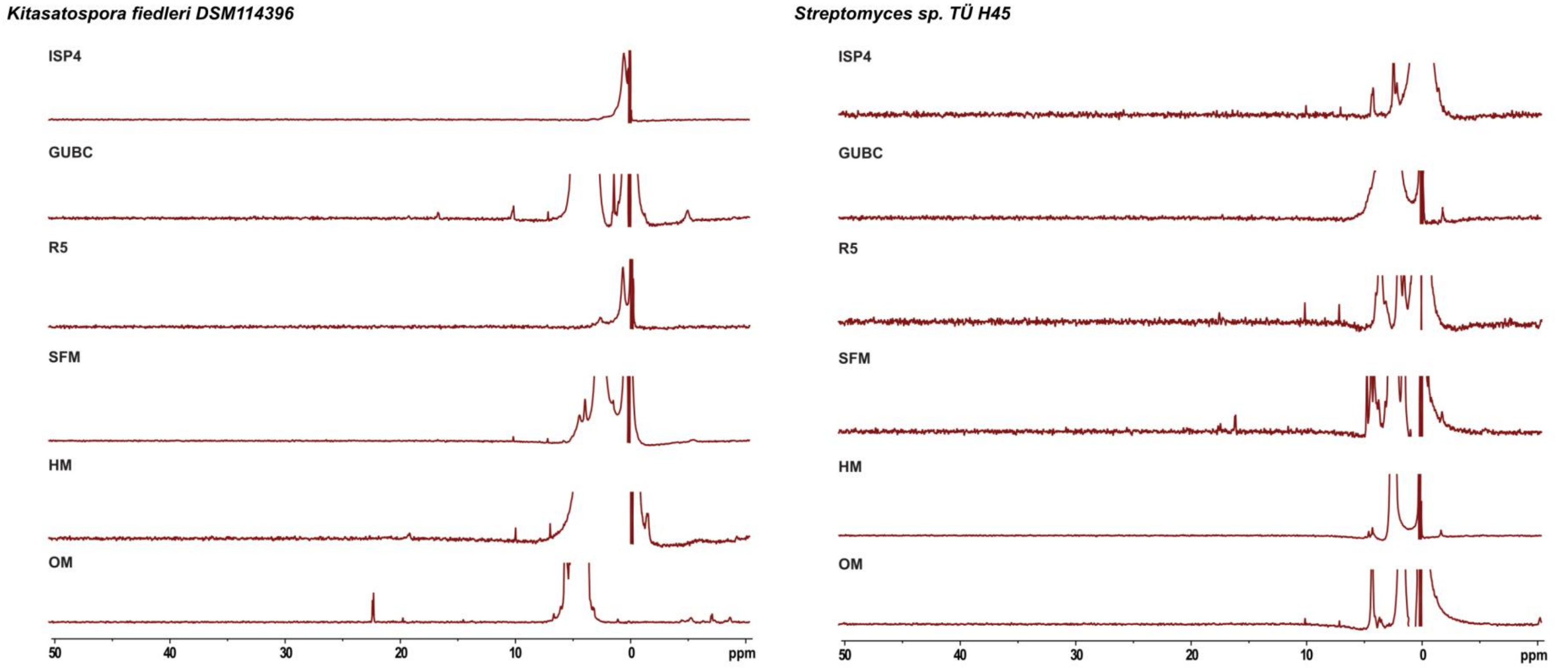

Figure S7. Synteny analysis for defining the boundaries of the putative P-BGC.

A. Similarity search with NCBI BLAST blastn program, using as query *Kitasatospora fiedleri* DSM 114396 chromosome sequence with accession NZ_OX419519, limited to the range 3391998-3439448, against the NCBI Database "Refseq prokaryote representative genomes (contains refseq assembly) Update date 2025/05/12" limited to "*Kitasatospora cineracea* (taxid:88074)", the closest known species to DSM 114396 and for which several genome assemblies are available. The result shows the highly conserved regions (see also *Fig. S8* **ABDG**) surrounding the putative phosphonate biosynthetic gene cluster, therefore helping to determine the boundaries of the gene cluster. The highly conserved region around position 3409997 in the image represents the segment 3410635 to 3413339 and contains the genes with locus_tag QMQ26_RS15735, QMQ26_RS15740 and QMQ26_RS15745, all of them homologs of the highly conserved genes with locus_tag QMQ26_RS09285 (*cysN*), QMQ26_RS09280 (*cysD*), and QMQ26_RS09275 (*cysC*), that span from position 1960006 to 1962829 of the chromosome of DSM 114396, and encode the highly conserved enzymes sulphate adenylyltransferase subunit 1 CysN [EC:2.7.7.4; KEEG K00956], sulphate adenylyltransferase subunit 2 CysD [EC:2.7.7.4; KEEG K00957], and adenylylsulphate kinase CysC [EC:2.7.1.25; KEEG K00860] from sulphur metabolism in bacteria (see also Fig. S3 and S5). The upstream end (right in Figure S3) is supported by a synteny analysis with the type strains of *Kitasatospora cineracea* and *Kitasatospora niigatensis*, the most closely related species to *K. fiedleri* lacking a P-BGC[1] (Figure S3).The downstream end (left in the figure) is well-supported also by highly conserved homologous genes beyond the aldehyde dehydrogenase, with the first gene (encoding a chloride channel protein) showing 69-97% identity to *Kitasatospora* strains in the NCBI non-redundant protein database. **B and C.** Artemis Comparison Tool (ACT) visualisation of the NCBI BLAST analysis of DSM 114396 chromosome (NZ_OX419519) against *K. cineracea* DSM 44780 contig NZ_RJVJ01000001 (**B**) or against *Kitasatospora niigatensis* DSM 44781 contig NZ_RKQG01000001 (**C**), using NCBI megablast with default options. Note that the display of the bottom sequence has been flipped in both B and C to match the orientation of the top one. **D.** ACT visualisation of the NCBI BLAST analysis of NZ_OX419519 against *Kitasatospora setae* KM-6054 (the type strain of the genus) chromosome (NC_016109.1) showing that the synteny restarts only with the gene with locus_tag QMQ26_RS15885 encoding a tRNA-Thr, as a result of being *K. setae* a more distant species.

A

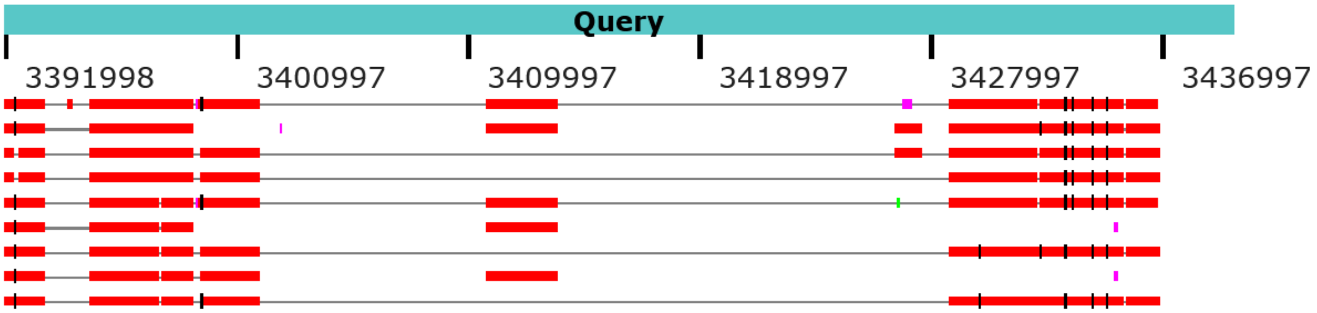

B

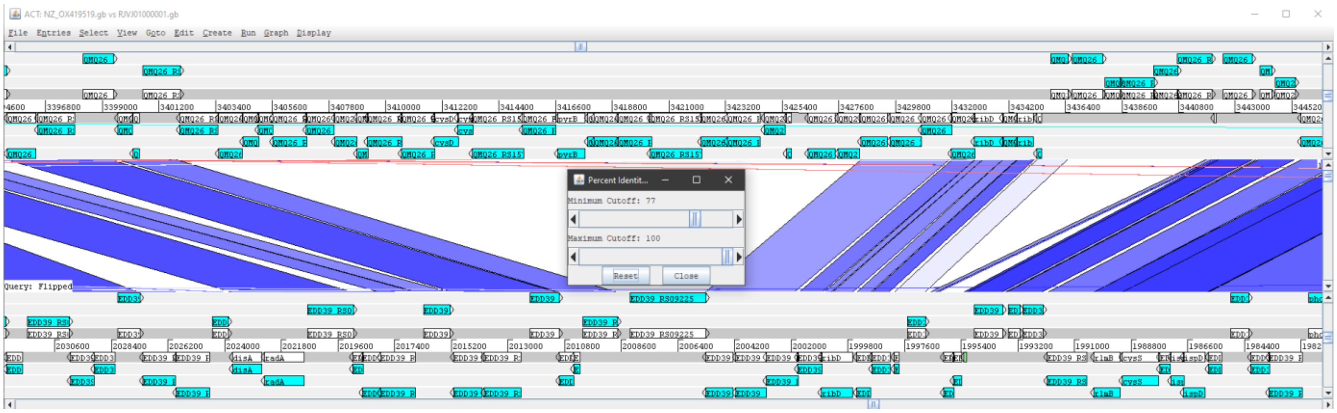
ACT: NZ_OX419519.gb vs RJVJ01000001.gb
File Entries Select View Goto Edit Create Run Graph Display
Percent Identit...
Minimum Cutoff: 77
Maximum Cutoff: 100
Reset
Close
Query: Flipped

C

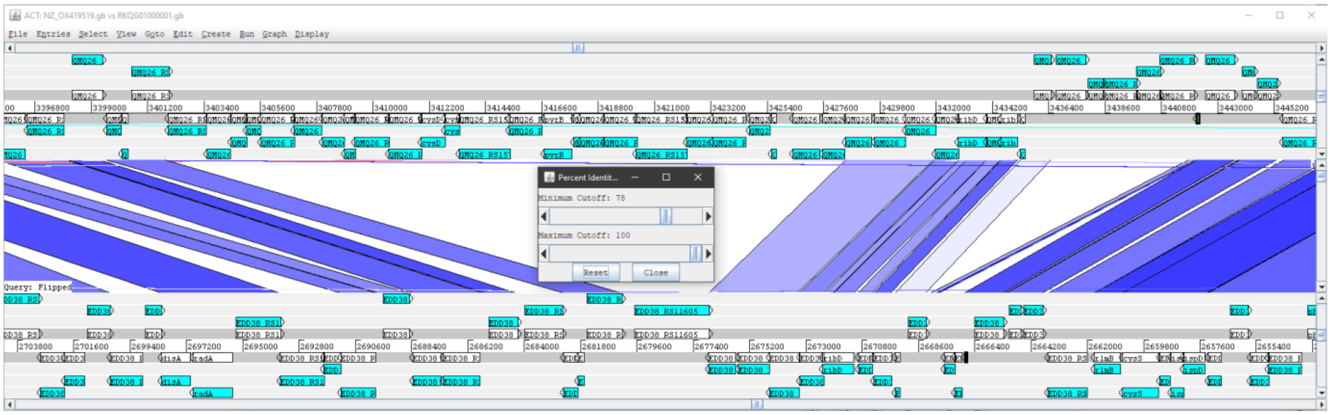
ACT: NZ_OX419519.gb vs RKQG01000001.gb
File Entries Select View Goto Edit Create Run Graph Display
Percent Identit...
Minimum Cutoff: 78
Maximum Cutoff: 100
Reset
Close
Query: Flipped

D

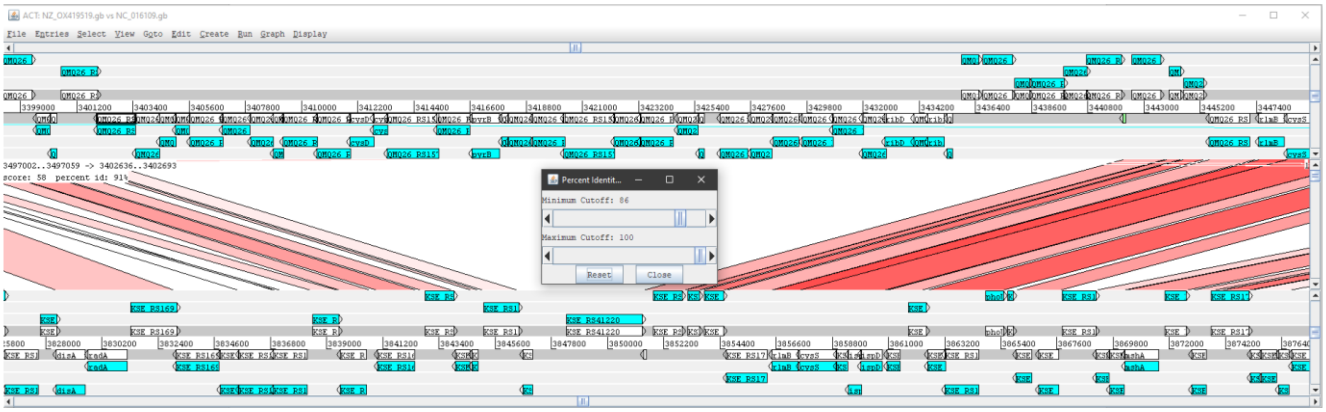
ACT: NZ_OX419519.gb vs NC_016109.gb
File Entries Select View Goto Edit Create Run Graph Display
3497002..3497059 -> 3402636..3402693
score: 58 percent id: 91%
Percent Identit...
Minimum Cutoff: 86
Maximum Cutoff: 100
Reset
Close

Figure S8. clinker analysis for defining the boundaries of the putative P-BGC.

An analysis at NCBI BLAST, using the range 3391998 to 3439448 of NZ_OX419519.1 was performed against the "RefSeq Genome Database", limited to "Actinobacteria (taxid: 201174)", and using the program "megablast" with default parameters. The graphical summary of the results is shown in panel **A**. The gene cluster, as delimited by synteny analysis with *Kitasatospora cineracea* DSM 44780 is framed in a blue box. All sequences that provided coverage of the BGC (and not just for the homologues of the highly conserved *cysCDN* genes for sulfur metabolism to the right of the 3409997 mark) originated from whole genome sequencing of microorganisms classified as *Streptomyces* or *Kitasatospora* species (table **B**); these alignments were thoroughly analysed, and the segments of the NCBI RefSeq records spanning 50 kb (or to the beginning or end in case the deposited sequence was not long enough) starting from 10 kb downstream of the aldehyde dehydrogenase gene, were downloaded as fully annotated GenBank files. These sequence files, together with the sequence comprising 3391998 to 3439448 of NZ_OX419519, were used for similarity analysis with CLINKER (with the default "Minimum alignment sequence identity" of 0.30). Table **B** shows the strains (with the accession numbers of the sequences used) included in CLINKER analysis, ordered according to CLINKER's similarity of the PepM encoded by each strain as compared to *K. fiedleri* PepM. **C.** Legend for the colour used in the CLINKER plot to group homologous genes. **D.** CLINKER plot, with BGCs sorted according to, first, phylogeny proximity (*Kitasatospora* strains at the top), and finally the order was adapted to highlight similarity and synteny along the entire BGC and surrounding genes; the plot is aligned at the aldehyde dehydrogenase gene (in red). The BGC from some strains is unfortunately broken in several contigs, what breaks the homology in CLINKER's plot, but rearranging these contigs would show complete synteny (not shown). It is noticeable that all strains carry the same genes and in the same order, including the gene encoding a LuxR-family transcriptional regulator (in fuchsia pink) and NUDIX-domain encoding gene (in red); curiously the LuxR gene is located to the other extreme of the BGC in the Strep*tomyces kanasensis/Streptomyces changanensis* group, that seems to also lack the NUDIX-domain encoding gene. Another clear difference is the presence of a gene encoding a putative FAD/NAD(P)-binding protein between the LuxR and NUDIX genes, only in the strains *Streptomyces* sp. NPDC053429, *Streptomyces* sp. NPDC051109 and *Streptomyces* sp. ISL-100. Other small differences might well be due to sequencing errors (that brake the reading frame of a gene and therefore the annotation of encoded protein) or differences in gene annotation (although by using RefSeq database all annotations are expected to be comparable as made with the same or similar pipeline by NCBI).

A phylogenetic analysis was performed with TYGS server to assess phylogenetic relationship among the *Kitasatospora* strains that carry a similar P-BGC to *K. fiedleri*. The full genome assemblies were downloaded from NCBI and submitted as queries to TYGS. Figure in panel **E** shows the strains included in the analysis and the taxonomical judgment provided by TYGS. Panel **F** shows the similarity statistics. Panel **G** shows the full-genome phylogenetic tree. The results indicate that the three strains that show full synteny of the P-BGC and surrounding genes in **D**, *Kitasatospora* sp. NPDC088783, *Kitasatospora* sp. SolWspMP and *Kitasatospora* sp. NPDC090308, actually belong to *K. fiedleri* species. The other two *Kitasatospora* strains that carry an almost identical P-BGC but with different surrounding genes in **D** (*Kitasatospora* sp. Root107 and *Kitasatospora* sp. Root187) fall in a different cluster in the phylogenetic tree, even farer-away than *Kitasatospora cineracea/Kitasatospora niigatensis* and *Kitasatospora setae*, strains all that do not carry any P-BGC.

A

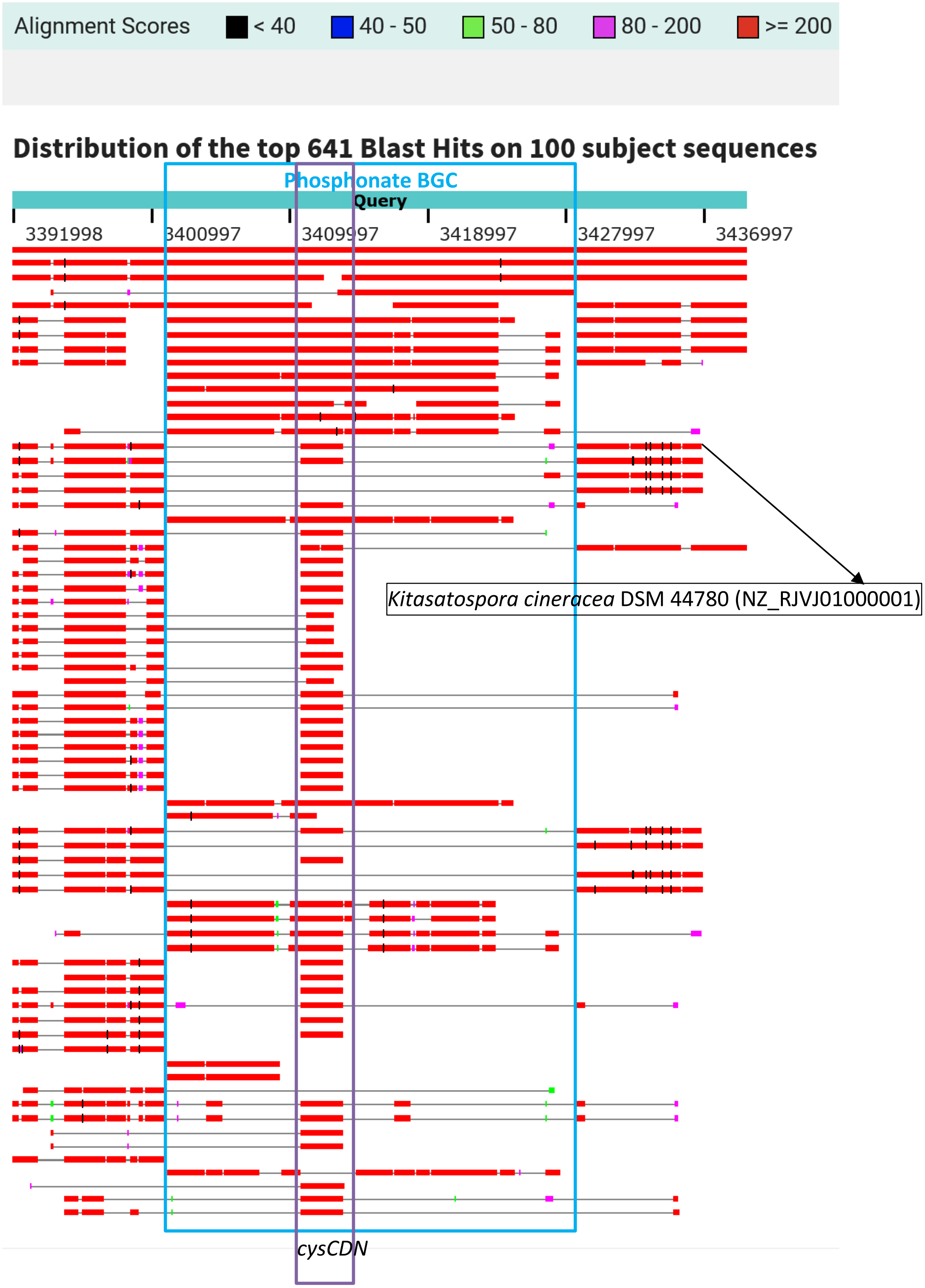
Alignment Scores
< 40
40 - 50
50 - 80
80 - 200
>= 200
Distribution of the top 641 Blast Hits on 100 subject sequences
Phosphonate BGC
Query
3391998
3400997
3409997
3418997
3427997
3436997
Kitasatospora cineracea DSM 44780 (NZ_RJVJ01000001)
cysCDN

B

**Strain source of PepM as compared against *K. fiedleri* DSM 114396 PepM**	**Identity**	**Similarity**
Kitasatospora sp. NPDC088783_185_F4.ctg-0008 NZ_JBIVKJ010000008	1	1
Kitasatospora sp. SolWspMP NZ_QLLT01000023	1	1
Kitasatospora sp. NPDC090308_182_E10.ctg-0012 NZ_JBIVXY010000012	1	1
Streptomyces sp. ISL-100 ISL100_59 NZ_JAGGOK010000059	0.87	0.91
Streptomyces sp. NPDC051109_71_F2.ctg-0006 NZ_JBITFF010000006	0.86	0.91
Streptomyces sp. MMG1121 P433contig3.1 NZ_LGDV01000190	0.86	0.91
Streptomyces sp. MMG1121 KF386872	0.86	0.91
Streptomyces sp. NPDC091268_165_H6.ctg-0011 NZ_JBIVVE010000011	0.85	0.91
Streptomyces sp. NPDC053429_373_F9.ctg-0003 NZ_JBITXY010000003	0.85	0.9
Streptomyces sp. R35 CP163440	0.84	0.91
Streptomyces sp. NPDC060198_T5_A10.ctg-0004 NZ_JBHWZJ010000004	0.84	0.91
Streptomyces sp. 31A4 KF386877	0.84	0.9
Streptomyces sp. NBC_00247 NZ_CP108093	0.84	0.9
Kitasatospora sp. Root107 contig_18 NZ_LMCT01000010	0.83	0.9
Kitasatospora sp. Root187 contig_15 NZ_LMHX01000007	0.83	0.9
Streptomyces odontomachi ODS25 24 NZ_JAMJWG010000024	0.81	0.89
Streptomyces sp. NPDC019224_87_D10.ctg-0005 NZ_JBEYCF010000005	0.8	0.89
Streptomyces kanasensis ZX01 contig00030 NZ_LNSV01000030	0.8	0.88
*Streptomyces changanensis*HL-66 NZ_CP102332	0.8	0.88
Streptomyces sp. CSDS2 NODE_17 NZ_JAUEPB010000017	0.8	0.88
Streptomyces kanasensis NPDC016311 NZ_JBIBSF010000001	0.8	0.87
Streptomyces kanasensis NPDC086832 NZ_JBIVAV010000006	0.8	0.87

C

- QMQ26_RS15720_TauD-TfdA family dioxygenase/QMQ26_RS15770_TauD-TfdA family dioxygenase
- QMQ26_RS15735_sulfate adenylyltransferase subunit 1_cysN
- QMQ26_RS15730_cation-proton antiporter
- QMQ26_RS15725_hypothetical protein/QMQ26_RS15745_adenylyl-sulfate kinase CysC_cysC
- QMQ26_RS15710_acetyl-CoA carboxylase family/QMQ26_RS15715_hypothetical protein
- QMQ26_RS15705_phosphonopyruvate decarboxylase subunit B_ppdB
- QMQ26_RS15700_phosphonopyruvate decarboxylase subunit A_ppdA
- QMQ26_RS15695_phosphoenolpyruvate mutase_pepM
- QMQ26_RS15690_Aldehyde dehydrogenase_adh
- QMQ26_RS15800_hypothetical protein
- QMQ26_RS15795_hypothetical protein
- QMQ26_RS15790_hypothetical protein
- QMQ26_RS15785_histidinol-phosphate transaminase
- QMQ26_RS15780_S9 family peptidase
- QMQ26_RS15775_hypothetical protein
- QMQ26_RS15765_hypothetical protein
- QMQ26_RS15760_aspartate carbamoyltransferase PyrB_pyrB
- QMQ26_RS15755_MFS transporter
- QMQ26_RS15750_inositol monophosphatase family
- QMQ26_RS15740_sulfate adenylyltransferase subunit CysD_cysD
- QMQ26_RS15810_NUDIX domain-containing protein [hydrolase activity]
- QMQ26_RS15805_LuxR C-terminal-related transcriptional regulator_luxR

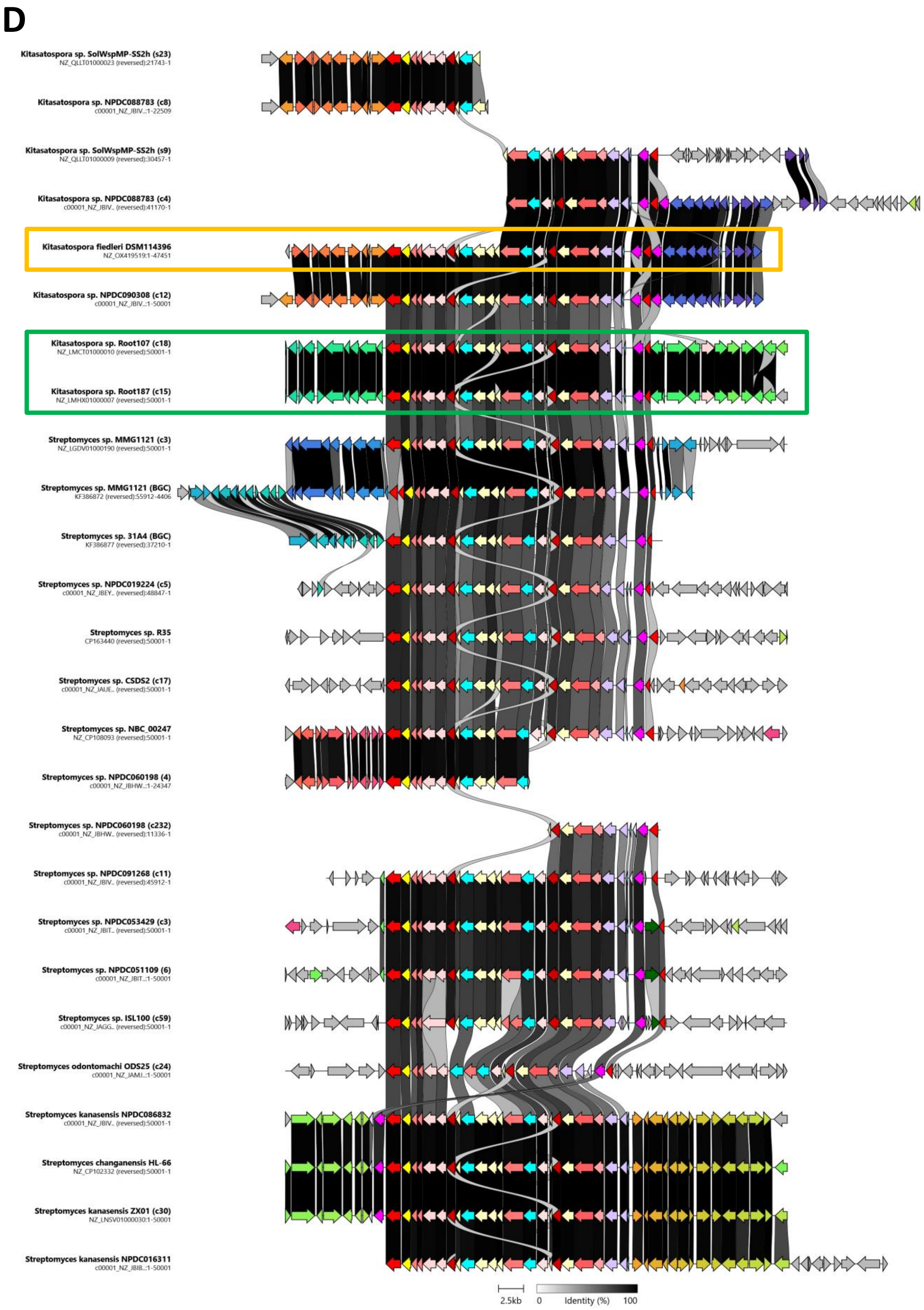
D
Kitasatospora sp. SolWspMP-SS2h (s23)
NZ_QLLT01000023 (reversed):21743-1
Kitasatospora sp. NPDC088783 (c8)
c00001_NZ_JBIV..:1-22509
Kitasatospora sp. SolWspMP-SS2h (s9)
NZ_QLLT01000009 (reversed):30457-1
Kitasatospora sp. NPDC088783 (c4)
c00001_NZ_JBIV.. (reversed):41170-1
Kitasatospora fiedleri DSM114396
NZ_OX419519:1-47451
Kitasatospora sp. NPDC090308 (c12)
c00001_NZ_JBIV..:1-50001
Kitasatospora sp. Root107 (c18)
NZ_LMCT01000010 (reversed):50001-1
Kitasatospora sp. Root187 (c15)
NZ_LMHX01000007 (reversed):50001-1
Streptomyces sp. MMG1121 (c3)
NZ_LGDV01000190 (reversed):50001-1
Streptomyces sp. MMG1121 (BGC)
KF386872 (reversed):55912-4406
Streptomyces sp. 31A4 (BGC)
KF386877 (reversed):37210-1
Streptomyces sp. NPDC019224 (c5)
c00001_NZ_JBEY.. (reversed):48847-1
Streptomyces sp. R35
CP163440 (reversed):50001-1
Streptomyces sp. CSDS2 (c17)
c00001_NZ_JAUE.. (reversed):50001-1
Streptomyces sp. NBC_00247
NZ_CP108093 (reversed):50001-1
Streptomyces sp. NPDC060198 (4)
c00001_NZ_JBHW..:1-24347
Streptomyces sp. NPDC060198 (c232)
c00001_NZ_JBHW.. (reversed):11336-1
Streptomyces sp. NPDC091268 (c11)
c00001_NZ_JBIV.. (reversed):45912-1
Streptomyces sp. NPDC053429 (c3)
c00001_NZ_JBIT.. (reversed):50001-1
Streptomyces sp. NPDC051109 (6)
c00001_NZ_JBIT..:1-50001
Streptomyces sp. ISL100 (c59)
c00001_NZ_JAGG.. (reversed):50001-1
Streptomyces odontomachi ODS25 (c24)
c00001_NZ_JAMJ..:1-50001
Streptomyces kanasensis NPDC086832
c00001_NZ_JBIV.. (reversed):50001-1
Streptomyces changanensis HL-66
NZ_CP102332 (reversed):50001-1
Streptomyces kanasensis ZX01 (c30)
NZ_LNSV01000030:1-50001
Streptomyces kanasensis NPDC016311
c00001_NZ_JBIB..:1-50001
2.5kb
0
Identity (%)
100

E

Table 2: Identification

- Your strain **'GCF_003259315.1_ASM325931v1_KitasatosporaSolWspMP-SS2h_genomic'** belongs to species *Kitasatospora fiedleri*.
- Your strain **'GCF_044750705.1_ASM4475070v1_KitasatosporaNPDC088783_genomic'** belongs to species *Kitasatospora fiedleri*.
- Your strain **'GCF_044769485.1_ASM4476948v1_KitasatosporaNPDC090308_genomic'** belongs to species *Kitasatospora fiedleri*.
- Your strain **'GCF_948472415.1_JK4103_genomic'** belongs to species *Kitasatospora fiedleri*.
- Your strain **'GCF_003751605.1_ASM375160v1_Kit.cineracea_DSM44780_genomic'** belongs to species *Kitasatospora niigatensis*. **Note:**
- Your strain **'GCF_003814885.1_ASM381488v1_Kit.niigatensis_DSM 44781_genomic'** belongs to species *Kitasatospora niigatensis*. **Note:**
- Your strain **'GCF_000269985.1_ASM26998v1_Kit.setaeKM-6054_genomic'** belongs to species *Kitasatospora setae*.
- Potential new species detected: your strain **'GCF_001424875.1_KitasatosporaRoot107_genomic'** does not belong to any species found in TYGS database.
- Potential new species detected: your strain **'GCF_001429805.1_KitasatosporaRoot187_genomic'** does not belong to any species found in TYGS database.
- Potential new species detected: your strain **'Strep_31A4_SRR7783782_contigs'** does not belong to any species found in TYGS database.

F

Query strain	Subject strain	dDDH (d_0, in %)	C.I. (d_0, in %)	dDDH (d_4, in %) ★	C.I. (d_4, in %)	dDDH (d_6, in %)	C.I. (d_6, in %)	G+C content difference (in %)
'GCF 000269985.1 ASM26998v1 Kit.setaeKM-6054 genomic'	*Kitasatospora fiedleri* DSM 114396	44.5	[41.1 - 47.9]	37.3	[34.8 - 39.8]	42.4	[39.4 - 45.4]	0.15
'GCF 001424875.1 KitasatosporaRoot107 genomic'	*Kitasatospora fiedleri* DSM 114396	23.5	[20.3 - 27.2]	24.1	[21.8 - 26.5]	22.6	[19.7 - 25.6]	2.63
'GCF 001429805.1 KitasatosporaRoot187 genomic'	*Kitasatospora fiedleri* DSM 114396	23.7	[20.4 - 27.3]	24.0	[21.7 - 26.5]	22.6	[19.8 - 25.7]	2.58
'GCF 003259315.1 ASM325931v1 KitasatosporaSolWspMP-SS2h genomic'	*Kitasatospora fiedleri* DSM 114396	79.6	[75.6 - 83.0]	70.0	[67.0 - 72.8]	80.6	[77.3 - 83.6]	0.41
'GCF 003751605.1 ASM375160v1 Kit.cineracea DSM44780 genomic'	*Kitasatospora fiedleri* DSM 114396	50.2	[46.8 - 53.7]	42.9	[40.4 - 45.5]	48.7	[45.6 - 51.7]	0.14
'GCF 003814885.1 ASM381488v1 Kit.niigatensis DSM 44781 genomic'	*Kitasatospora fiedleri* DSM 114396	52.3	[48.8 - 55.7]	42.8	[40.3 - 45.4]	50.4	[47.3 - 53.4]	0.02
'GCF 044750705.1 ASM4475070v1 KitasatosporaNPDC088783 genomic'	*Kitasatospora fiedleri* DSM 114396	75.4	[71.4 - 79.0]	74.3	[71.3 - 77.1]	77.9	[74.4 - 81.0]	0.11
'GCF 044769485.1 ASM4476948v1 KitasatosporaNPDC090308 genomic'	*Kitasatospora fiedleri* DSM 114396	85.3	[81.6 - 88.3]	74.2	[71.2 - 77.0]	86.2	[83.1 - 88.8]	0.25
'GCF 948472415.1 JK4103 genomic'	*Kitasatospora fiedleri* DSM 114396	100.0	[100.0 - 100.0]	100.0	[100.0 - 100.0]	100.0	[100.0 - 100.0]	0.0
'Strep 31A4 SRR7783782 contigs'	*Kitasatospora fiedleri* DSM 114396	15.1	[12.3 - 18.6]	21.4	[19.1 - 23.8]	15.3	[12.8 - 18.2]	3.03

G

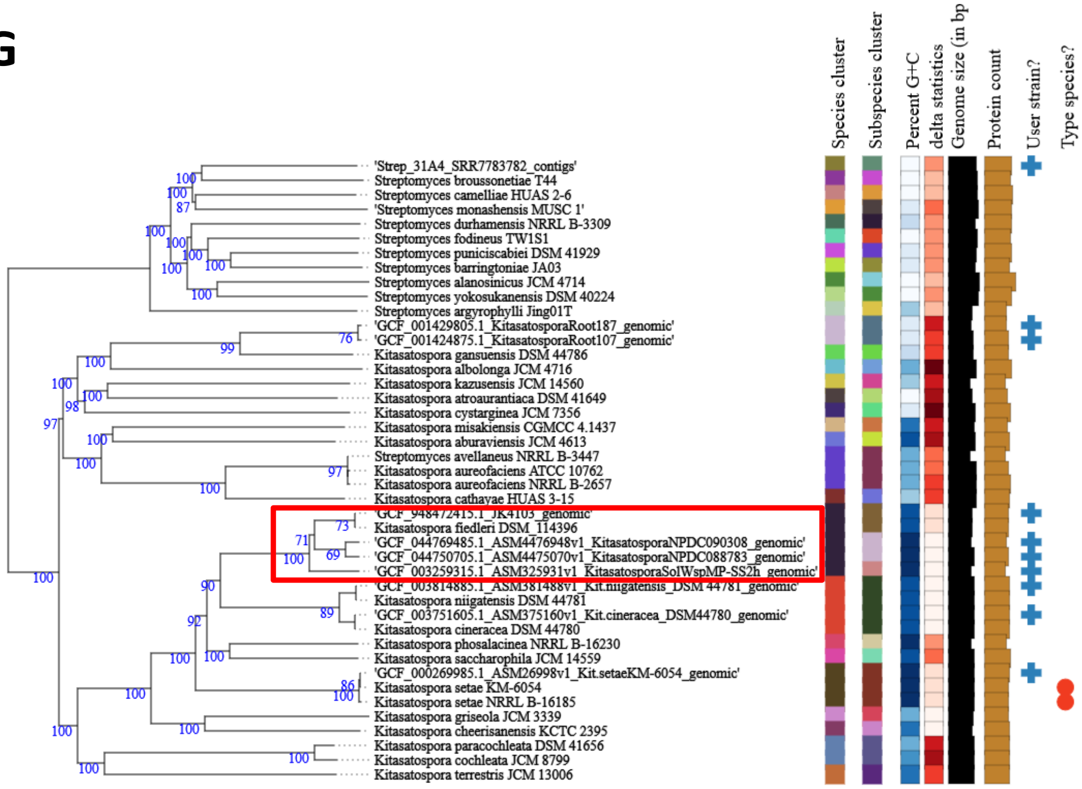
Species cluster
Subspecies cluster
Percent G+C
delta statistics
Genome size (in bp
Protein count
User strain?
Type species?
'Strep_31A4_SRR7783782_contigs'
Streptomyces broussonetiae T44
Streptomyces camelliae HUAS 2-6
'Streptomyces monashensis MUSC 1'
Streptomyces durhamensis NRRL B-3309
Streptomyces fodineus TW1S1
Streptomyces puniciscabiei DSM 41929
Streptomyces barringtoniae JA03
Streptomyces alanosinicus JCM 4714
Streptomyces yokosukanensis DSM 40224
Streptomyces argyrophylli Jing01T
'GCF_001429805.1_KitasatosporaRoot187_genomic'
'GCF_001424875.1_KitasatosporaRoot107_genomic'
Kitasatospora gansuensis DSM 44786
Kitasatospora albolonga JCM 4716
Kitasatospora kazusensis JCM 14560
Kitasatospora atroaurantiaca DSM 41649
Kitasatospora cystarginea JCM 7356
Kitasatospora misakiensis CGMCC 4.1437
Kitasatospora aburaviensis JCM 4613
Streptomyces avellaneus NRRL B-3447
Kitasatospora aureofaciens ATCC 10762
Kitasatospora aureofaciens NRRL B-2657
Kitasatospora cathayae HUAS 3-15
'GCF_948472415.1_JK4103_genomic'
Kitasatospora fiedleri DSM_114396
'GCF_044769485.1_ASM4476948v1_KitasatosporaNPDC090308_genomic'
'GCF_044750705.1_ASM4475070v1_KitasatosporaNPDC088783_genomic'
'GCF_003259315.1_ASM325931v1_KitasatosporaSolWspMP-SS2h_genomic'
'GCF_003814885.1_ASM381488v1_Kit.niigatensis_DSM 44781_genomic'
Kitasatospora niigatensis DSM 44781
'GCF_003751605.1_ASM375160v1_Kit.cineracea_DSM44780_genomic'
Kitasatospora cineracea DSM 44780
Kitasatospora phosalacinea NRRL B-16230
Kitasatospora saccharophila JCM 14559
'GCF_000269985.1_ASM26998v1_Kit.setaeKM-6054_genomic'
Kitasatospora setae KM-6054
Kitasatospora setae NRRL B-16185
Kitasatospora griseola JCM 3339
Kitasatospora cheerisanensis KCTC 2395
Kitasatospora paracochleata DSM 41656
Kitasatospora cochleata JCM 8799
Kitasatospora terrestris JCM 13006

Figure S9. Visualization of the putative P-BGC and GC-Frame plot in Artemis.

An inspection with Artemis and the GC-Frame Plot function, combined with an NCBI blast search of encoded proteins, indicates that there are no apparent errors in the sequence or CDS annotation of *Kitasatospora fiedleri* P-BGC[2].

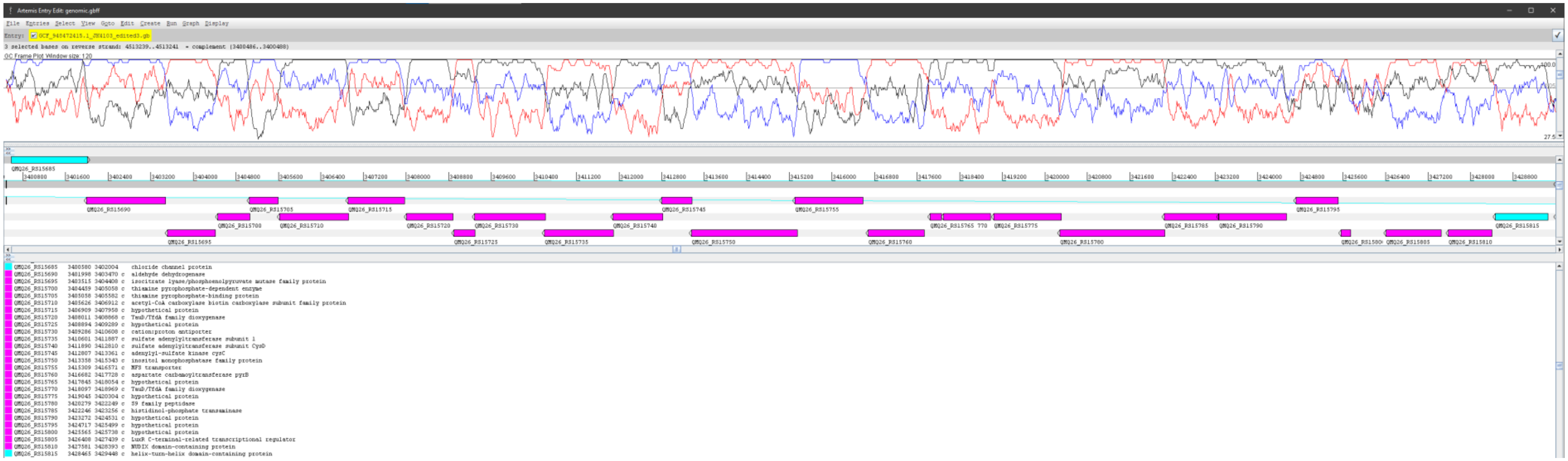

Figure S10. ^{31}P NMR spectra of concentrated supernatants from heterologous expression strains

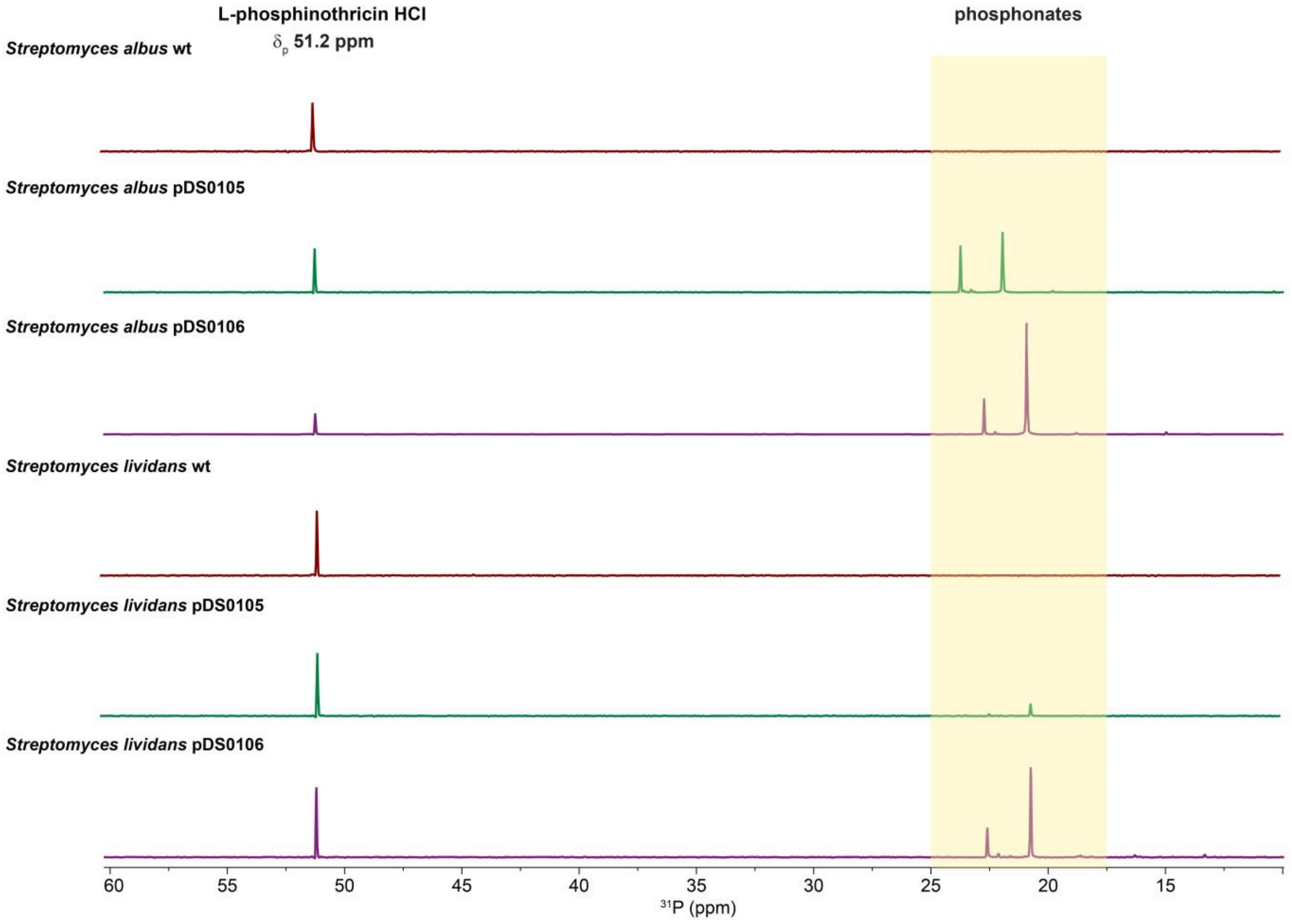

Figure S11. BLAST analysis of amino acid sequences from the *Kitasatospora fiedleri* P-BGC

Each amino acid sequence encoded by a predicted Protein Codding Sequence (CDS) from the antiSMASH predicted phosphonic acid cluster of *Kitasatospora fiedleri* DSM 114396^{T} was entered in a BLASTp search of the non-redundant protein sequences (limited to taxid2: bacteria) database and the number of proteins in the top 100 hits with sequence identity ≥70% (green), ≥50% (yellow), and <50% (orange) was tabulated revealing regions of high and low homology, respectively.

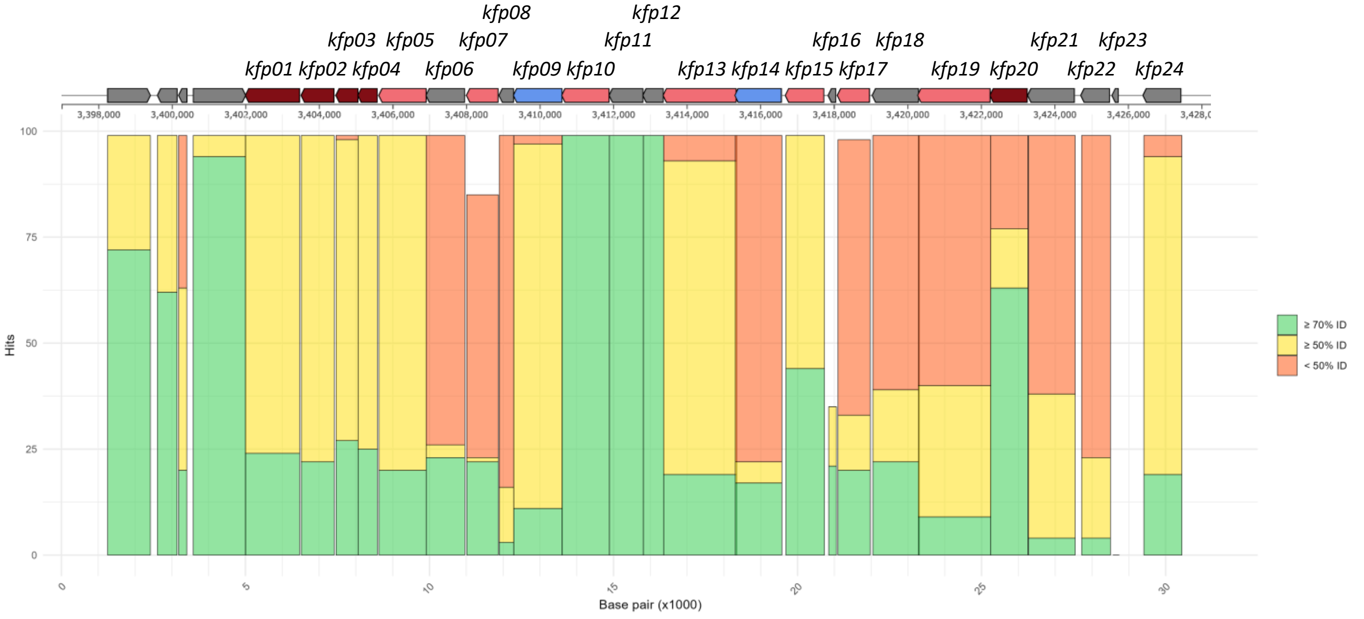

Figure S12. Alignment of aldehyde dehydrogenase enzymes from P-BGCs

(Next page) Alignment of the putative aldehyde dehydrogenase enzymes from the P-BGCs of *Kitasatospora fiedleri* DSM 114396 (Kfiedleri_adh; locus_tag="QMQ26_RS15690" of NZ_OX419519), *Streptomyces* sp. MMG1121 (MMG1121_adh; locus_tag="ADK64_RS20140" of NZ_LGDV01000190), *Streptomyces* sp. 31A4 (31A4_orf24; protein_id="AGZ94408.1 of KF386877), *Streptomyces* sp. NRRL F-525 proven phosphonoacetaldehyde dehydrogenase[3] (F-525_fpnG; protein encoded by sequence range complement(102849..104318) of JNXE01000019.1). A second putative acetaldehyde dehydrogenase encoding gene is present in the proximity of PepM in *Streptomyces* sp. NRRL F-525, just upstream of *fpnA* (*orf5* in Supplementary Figure 1 of Freestone et al 2017); we have included this protein in the alignment (F-525_adh2; protein encoded by sequence range 94228..95685 of JNXE01000019.1). On first position, the sequence of the best characterized phosphonoacetaldehyde dehydrogenase enzyme, PhnY from *Sinorhizobium meliloti* 1021[4]. Highlighted in blue or pink background and bold type are the residues important for activity according to the Supplementary Figure 1 of Agarwal et al, 2014. In all cases, all of the catalytic residues (pink) are conserved across all proteins. The residues involved in the phosphate/phosphonate binding pocket (blue) are completely conserved across all proteins proven (FpnG) or proposed to be involved in phosphonate biosynthesis in *Kitasatospora* and *Streptomyces*, and half of the residues also conserved with *S. meliloti*. However, the second putative aldehyde dehydrogenase from *Streptomyces* sp. NRRL F-525, not thought to be involved in phosphonate biosynthesis, is more variable at these important residues.

```
Smelilo_PhnY       --MTNAEVTIAVRHEPMRIAGRLVDT--DDRVEVRYPWND-TVVGTVPAGRAEHAREAFA 55
F-525_FpnG         MSLSVEIRADRIHRGAHLVGGQHVQG--DALLEKVNPARPGTSLGRIPVADRGVVDSAVR 58
31A4_orf24         MELRRAVADRAVADAHNHIAGRWCDARSGRTQENRNPGDLTEVVSRSADSGADDAQAAVA 60
MMG1121_adh        ------VADRAVADARNHIAGRWCDARSGRTQEHRNPGDLTEVVSRSADSATADAQAAVA 54
Kfiedleri_adh      MEPR-LTAGPRLAEARNHVAGRWCGAASGRSRDNRNPGDLAETVSRSADSAAADALAAVA 59
F-525_adh2         ----------MITYDRLFIAGSWTVPSSPDLLDIRSPHDR-SVIGRAAQALPADVDRSVA 49
                              :      :.*           :   *      :. .  .    .  :.

Smelilo_PhnY       IAAAYQP-----KLTRYERQKILLATAEALAARKEEISDVITLELG---ISKADSLYEVG 107
F-525_FpnG         TAHEQLA--AWRKLGSIARADLVHRLAGLMERDRGELALLITEEHG---KTRAEADSEVA 113
31A4_orf24         AARRALP--AWQALGSVRRGEIVMRAARLLAERRQEFAETITREQG---KLLSEAFGEVD 115
MMG1121_adh        AARAALP--AWQSLGSVRRGEIVMRAARLVAERRQEFAETITREHG---KLLSEAFGEVD 109
Kfiedleri_adh      AARAALA--AWRSLGPVRRGEVVLRAARLVAERRAEFAAITREQG---KLPHEALGEVD 114
F-525_adh2         AARAAFDEGPWPRTAPAERIAVIRRLNALREADAEKIAALISAENGSAGWFTLAGQPGLG 109
                    *                *  ::            :::  *: * *        .   :

Smelilo_PhnY       RAFDVFTLAGQMCIRDDGEIFSCDLTPHGKARKIFTMREPLTAISAITPFNHPLNMVAHK 167
F-525_FpnG         RSIEIVRFAAGLGRRLGG----RTLPSDSPDTWSSTTRHPIGVVGLITPWNFPLAIPVWK 169
31A4_orf24         RTVDLLEYTAGEGRRLGG----ATLPADNPRTLALTRREPIGVVGLVTPWNFPLAIPAWK 171
MMG1121_adh        RTVDVLEYTAGEGRRLGG----ATLPADNPRTLALTRREPIGVVGLVTPWNFPLAIPAWK 165
Kfiedleri_adh      RTVALLEFTAGEGRRLGG----ATLPADDPRTLALTRRDPIGVVALITPWNFPLAIPAWK 170
F-525_adh2         RQAGAYLKAAEELGWEET----LAPSDPGSPTRSVLRREPIGVVAAVIPWNSPFSAALAK 165
                   *       :.               .  .        *.*: .:. : *:* *:     *

Smelilo_PhnY       VAPAIATNNCVVVKPTELTPMTALLLADILYEAGLPPEMLSVVTGWPADIGMEMITNPHV 227
F-525_FpnG         LAPALVAGCTVVLKPSPLAPFTAQRLVTLAHEAGVPAGVVNLVHGD-GPTGAALVEHPLV 228
31A4_orf24         VAPALLAGCTSVLKPSPFTPLSASLLVDCFVQAGAGGGALNLVHGG-REVGEALVADPAV 230
MMG1121_adh        VAPALLAGCTSVLKPSPLTPLTASLLVDCFVQAGAGGGALNLVHGG-REVGEALVADPVV 224
Kfiedleri_adh      VAPALLSGCTAVLKPSPLTPLTATLLVDCFVEAGAGHGVLNLVHGG-REVGEALVDHPDV 229
F-525_adh2         VIPALLAGNTVVLKVSPENSLSMGYLAELLERSNLPEGVISVLPAD-RETSEYLVSHPGV 224
                   : **: :.   *:* :   .::   *.    .:.     :.:: .     .  :: .* *

Smelilo_PhnY       DLVTFTGSVPVGKLIAAN--AHYKRQVLELGGNDPLIILNDLSDDDLARAADLAVAGATK 285
F-525_FpnG         KGVSFTGSVDVGRSVQAAAAASLTRTQLEMGGKNAVVVL---ADADLEKAADAVAHGAFG 285
31A4_orf24         AGISFTGSVEVGRAIHTAGAPRFLRTQLEMGGKNAALVL---PDADLDEAADAITAGAFG 287
MMG1121_adh        AGISFTGSVEVGRAIHTAGAPRFLRTQLEMGGKNAALVL---ADADLDEAADAITAGAFG 281
Kfiedleri_adh      AGVSFTGSVEVGRAIHVAGAPRFLRTQLEMGGKNAALVL---ADADLDRAADAIVAGAFG 286
F-525_adh2         DKIAFTGSTGAGRRIASIAGEQLKRVSLELGGKSAAVIL---DDADVQQAVQGLKFASLM 281
                     ::****. .*: :         *  **:**:.. ::*    * *: .*.:    .:

Smelilo_PhnY       NSGQRCTAVKRILCQESVADRFVPLVLERAKRLRFGDPMDRSTDLGTVIHEKAAALFEER 345
F-525_FpnG         QAGQRCSATSRAIVQDDVFDEFVARLAARASALRVGRPDDPLTDLGPLINRASLERCLDA 345
31A4_orf24         QAGQRCSATSRVVVDRAVHRELVGRLAERATGLRVGTGMDPAARLGPVVSAERLEACLNG 347
MMG1121_adh        QAGQRCSATSRVVVDRSVHRELVARLAERAAGLRVGTGMDTAAALGPVISAERLEACLNG 341
Kfiedleri_adh      QAGQRCSATSRVVVDRAVHDALVRRLAARAEALRVGPGTDPDARLGPVVSAERLHACLEG 346
F-525_adh2         NNGEACIAQTRVLAPRGRYEEVVTALKDLVESLKVGDPNDPDTFIGPMVRPDQQQRVRDY 341
                   : *: * * .* :        .*  :   .  *:.*   *  : :*.::         :

Smelilo_PhnY       VMRAAEEGADILYHPGRS------GALLPPIVVDRVPHQSDLVLEETFGPIIPIVRVPDD 399
F-525_FpnG         VSAAVRDGARLVTGGTAVVTDD-GGYFMAPTVLTGIAADAPLANTEVFGPILVVLRCRDF 404
31A4_orf24         ILLATAEGAVVVTGGHRVTEGVPEGYFMAPTVLDEVRPDSYIAQEEIFGPVLSVIVCDGL 407
MMG1121_adh        ILLATAEGAVVVSGGHRVTEGVPEGYFMAPTVLDGVRPDSYIAQEEIFGPVLSVLVCDGL 401
Kfiedleri_adh      VRRATADGATVVTGGGRLTEGLPDGYFMAPTVLDLVRPDSHVAQEEIFGPVLSVITCDGP 406
F-525_adh2         IELGIKEGARLVTGGPQVPPGLEGGNYVTPTVFADVDNSMRIAQEEIFGPVLVVIPYDDE 401
                   :  .  :** ::            *  :.* *.  :  .  :.  * ***:: ::   .

Smelilo_PhnY       DDATITLSNSTAFGLSSGVCTNDYRRMQKYIAGLKVGTVNIWEVPGYRIEMSPFGGIKDS 459
F-525_FpnG         DEA-VAINNSVRYGMSATLFTASLPLIGRFLTEAEAGMLHVNRPGVGAFPHMPHIGTKES 463
31A4_orf24         DDG-LRIVNSVRYGMAAAVFTRNPSLALEALDRVEAGMLHVNRPGVGAYPHMPHIGAKDS 466
MMG1121_adh        DDG-LRIVNSVRYGMAAAVFTRNPSLALEALDRVEAGMLHVNRPGVGAYPHMPHIGAKES 460
Kfiedleri_adh      EDG-LRIVNGVRYGMAAAVFTRDTSLALDALDRIDVGMLHVNRPGVGAYPHMPHIGAKES 465
F-525_adh2         DDA-VRIANDSEYGLSGGVWSADQAHALAVARRLRTGTVTVN--GAPIAFDGPFGGFKAS 458
                   ::. : : *.  :*::. : : .            .* : :           *. * * *

Smelilo_PhnY       GNGYKEGVIEAMKSFTNVKTFSLPWP-- 485
F-525_FpnG         QVGPAECADDAIEFFTELRTATIGVG-- 489
31A4_orf24         QYGPAECSPQVWDFYTQWRTACISY--- 491
MMG1121_adh        QYGPAECSPQVWDFYTQWRTACISY--- 485
Kfiedleri_adh      QYGPAECSPQVWDFYTELRSACISY--- 490
F-525_adh2         GIG-REYGAVGLAQYVEYKTVTLPPAVS 485
                     *  *        :.: ::  :
```

Figure S13. Overview of characterized enzyme functions in phosphonate biosynthetic pathways detected as predicted proteins encoded in *K. fiedleri* pBGC

Some genes of the *K. fiedleri* P-BGC are predicted to be proteins which play known roles in characterized phosphonate biosynthetic pathways. **A** shows the reactions catalyzed by VlpG and VlpF, two ATP-grasp ligases (*kfp05* is predicted to encode an ATP-grasp enzyme) from the valinophos and DHPPA-phosphonate peptide pathway in *S. durhamensis* DSM 40539. VlpG catalyzes the ATP-dependent ligation of L-Val to dihydroxypropylphosphonate (DHPPA) to yield DHPPA-Val (Δ*vlpG* strains are deficient in valinophos production). In contrast, VlpF is inactive toward acetyl-Val but instead uses ATP to attach a broad range of L-amino acids onto DHPPA-Val, producing a suite of DHPPA-dipeptides. R = Gly, Ala, Val, Ser, Thr, Leu, Ile, Met, Lys, Arg, Asn, Gln, Trp, Phe, His. Adapted from Zhang et al., 2022 **B** depicts the proposed reaction catalyzed by a TauD dioxygenase (*kfp07* and *kfp17* are predicted to encode TauD/TfdA family dioxygenases) in *Burkholderia pseudomallei* 1710a leading to the synthesis of 2-hydroxy-phosphonoacetate (2-HPnA) from phosphonoacetic acid (PnA). Adapted from Yu et al., 2013. **C** shows the PLP-dependent aminotransferase-catalyzed formation of phosphonoalanine (PnAla). Phosphonopyruvate (PnPy) is transaminated by a PLP-dependent aminotransferase (*kfp20* is predicted to encode the same type of enzyme) from *Streptomyces* sp. NRRL S-515 in the biosynthesis of phosphonoalamides. L-aspartate donates the amino group producing PnAla. Coexpression of PepM and the aminotransferase is sufficient to reconstitute PnAla biosynthesis. Adapted from Kayrouz et al., 2020.

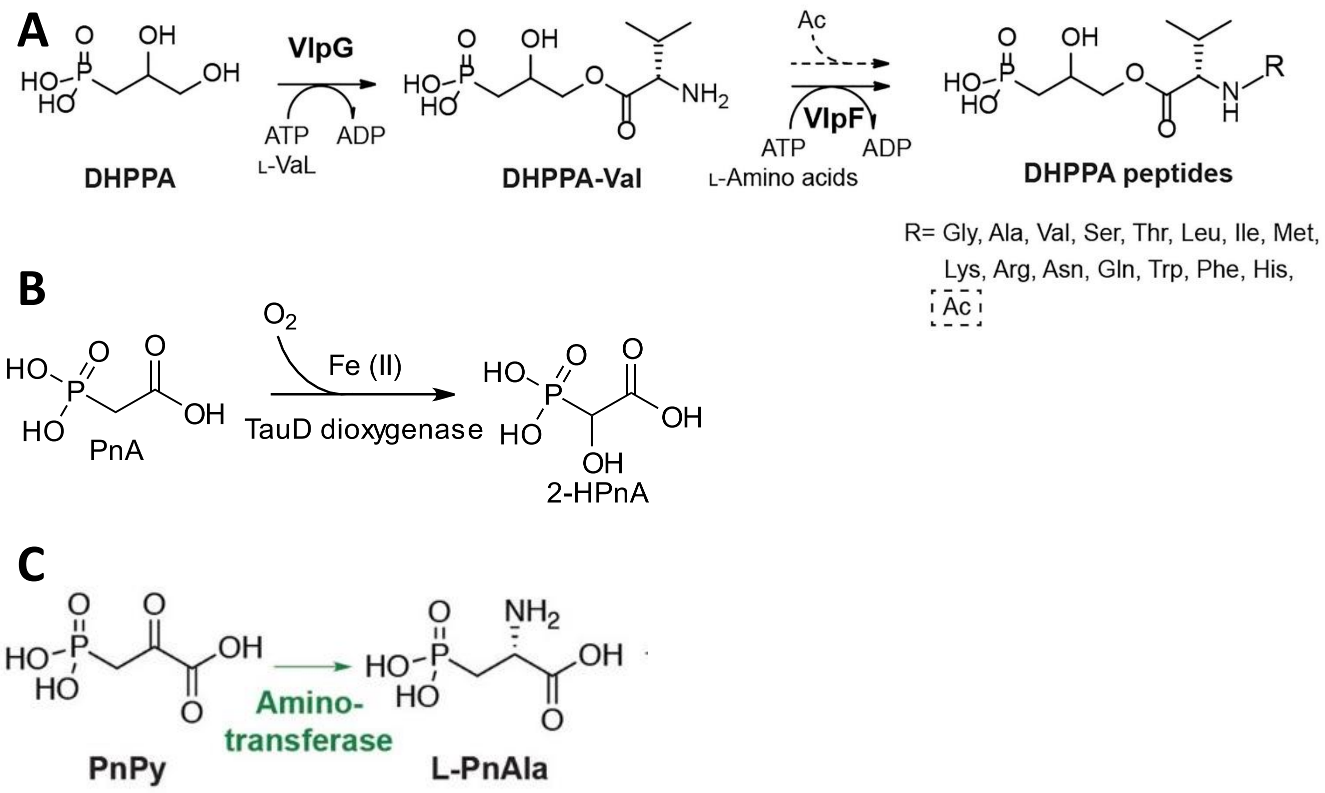

Table S1. Identified potential phosphonate producer strains from the DSMZ and Tübingen collection and known producers of reduced phosphorus compounds included in the analysis

Not all strains have publicly available genome information. (N/A).

Strain	strain designations	Genome accession	P-BGC accession	total no. BGCs	PepM sequence	PepM aa	PepM consensus motif (EDKX^5NS)	phosphonate product
**GCF01**								
Kitasatospora atroaurantiaca	DSM 41649	GCF_007828955.1	NZ_VIVR01000001.1	28	MQRVSYPSFRTVLGEHEIVRVAGANHALGGLLAEEAGFQAVWSSSLEVSASRGVPDASILSMAEYLESAANIQKVLRIPVVADCDTGYGGNLNVAHMVHEFEDAGITAVCIEDKLFPKMNSFVAGGQTLLDTAEFASKIVTAKRAQSTDDMFVIARTEALISGLDVAAALERCRAYADSGADAVLVHSKAKSRDQVLGFLEGWDFRCPVVIVPTTYPDWHIDEIKAAGVSVVIYANQGLRATVTALRDTYRAVYRHGDSTVVEDSIASVQDIFDLQRLPEWQKLDA	286	EDKLFPKMNS	unknown
Streptomyces bikiniensis	DSM 40581, ISP-5580	GCF_000716625.1	NZ_JNXJ01000013.1	30	MLADNEIVRVAGANHALGGLLAEEAGFQAVWSSSLEVSASRALPDASILSMTEYLEAAANIQKALRIPVVADCDTGYGGNLNVAHMVHEFEAAGITAICIEDKLFPKMNSFVAGGQTLLGTKEFADKIRIAKAAQATKEMFVIARTEALISGLDVDAALERCRAYADAGADAVLVHSKVKTRDQVVGFLEGWDFRCPVVIVPTTYPDWHIDEIKQAGVSVVIYANQGLRATVSALRETYRSVYENGDSTAVEDSIASVQDVFALQRLEAWQKLDV	275	EDKLFPKMNS	unknown
Streptomyces hokutonensis	DSM 102214, R1-NS-10	GCF_000376565.1	NZ_BARG01000110.1	35	MQRPIYPSFRSVLADNDVVRVAGANHALGGLLAEEAGFQAVWSSSLEVSASRGLPDASILSMTEYLEAAANIQKVLRIPVVADCDTGYGGNLNVGHMVHEFEAAGITGLCLEDKLFPKMNSFVAGGQTLLGTKEFARKIKIAKEAQASKDTFVIARTEALISGLDVAEALDRCRTYADSGADAVLVHSKAKTRDQVVAFLDGWDFRVPVVIVPTTYPDWHIDDIRQAGVSVVIYANHGLRATVSALRDTYRSVYENGDTTALEGSIASVKDIFALQGLDSWQKLDS	286	EDKLFPKMNS	unknown
Streptomyces regensis	DSM 40551, NRRL B-11479	GCA_001047335.1	LFVR01000169.1	49	MQRPIYPSFRSVLADNDVVRVAGANHALGGLLAEEAGFQAVWSSSLEVSASRGLPDASILSMTEYLEAAANIQKVLRIPVVADCDTGYGGNLNVAHMVHEFEAAGITGLCIEDKLFPKMNSFVAGGQTLLGTKEFAKKIKIAKEAQASTETFVIARTEALISGLDVDAALERCRTYADSGADAVLVHSKAKTRDQVVAFLEGWDFRVPVVIVPTTYPDWHIEDIRKAGVSVVIYANHGLRATVSALRDTYRSVYENGDTTALEGSIASVKDIFALQGLDTWQKLDS	286	EDKLFPKMNS	unknown
Streptomyces regensis	NRRL WC-3744	(N/A)	KF594335 Region on contig edge.	(N/A)	MQRPIYPSFRSVLADNDVVRVAGANHALGGLLAEEAGFQAVWSSSLEVSASRGLPDASILSMTEYLEAAANIQKVLRIPVVADCDTGYGGNLNVAHMVHEFEAAGITGLCIEDKLFPKMNSFVAGGQTLLGTKEFAKKIKIAKEAQASTETFVIARTEALISGLDVDAALERCRTYADSGADAVLVHSKAKTRDQVVAFLEGWDFRVPVVIVPTTYPDWHIEDIRKAGVSVVIYANHGLRATVSALRDTYRSVYENGDTTALEGSIASVKDIFALQGLDTWQKLDS	286	EDKLFPKMNS	hydroxynitrilaphos[5]
Streptomyces sp.	TUE 18	(N/A)	(N/A)	34	MQRSIHPSFRSVLADNEIVRVAGANHALGGLLAEEAGFQAVWSSSLEVSASRALPDASILSMTEYLEAAANIQKALRIPVVADCDTGYGGNLNVAHMVHEFEAAGITAICIEDKLFPKMNSFAAGGQTLLGTKE	286	EDKLFPKMNS	unknown

Strain	strain designations	Genome accession	P-BGC accession	total no. BGCs	PepM sequence	PepM aa	PepM consensus motif (EDKX^{5}NS)	phosphonate product
					FADKIRTAKAAQATQDMFVIARTEALISGLDVDAALERARAYADAGADAVLVHSKVKTRDQVVGFLEGWDNRVPVVIVPTTYPDWHIDEIKQAGVSVVIYANQGLRATVSALRETYRSVYETGDSTAVENSIASVQDIFALQGLEAWQKLDV			
Streptomyces xanthophaeous	DSM 116597, 11.9	(N/A)	(N/A)	33	MQRSIYPSFRSVLAENEIVRVAGANHALGGLLAEEAGFQAVWSSSLEVSASRALPDASILSMTEYLEAAANIQKALRIPVVADCDTGYGGNLNVAHMVHEFEAAGITAICIEDKLFPKMNSFVAGGQTLIGTKEFADKIRIAKAAQATQEMFVIARTEALISGLDVDAALERCRAYADAGADAVLVHSKVKTRDQVVGFLEGWDFRVPVVIVPTTYPDWHVDEIKQAGVSVVIYANQGLRATVSALRDTYRSVYETGDSTAVEGSIASVQDVFALQRLEAWQKLDV	286	EDKLFPKMNS	unknown
**GCF02**								
Streptomyces sp.	NRRL S-481	GCF_000721955.1	NZ_KL585164.1	29	MLKRSYPSFRSVLDAHDVVRLAGAHHALSGVLAQQAGFQAVWSSSLEVSASRCLPDASILSMTEYLEAAANIQKALDIPVVADVDTGYGGNYNVAHMVHEFEGAGITAVCMEDKLFPKMNSFANGDQTLLDTAAFTGKIQTAKAVQSTEDFFLIARTEALISGAGVDEAVSRCNAYADAGADAVLIHSKARTNEEVLAFLDRWDNRVPVVIVPTTYPDWHIDEVRAAGVSVVIYANQGMRAALSAMRNTYRAIYEAGHTTLLESEIASVRDIFDLQRMADWQKLDA	286	EDKLFPKMNS	phosphono-cystoximate[6]
Streptomyces sp.	I6	GCF_003725745.1	NZ_RHDP01000001.1	29	MLKRSYPSFRSVLAAHDVVRLAGAHHALSGVLAQEAGFQAVWSSSLEVSASRCLPDASILSMTEYLEAAANIQKALDIPVVADVDTGYGGNYNVAHMVNEFEGAGITAVCMEDKLFPKMNSFANGDQTLLDTAAFACKIRTAKTVQSTDDFFVIARTEALISGAGVDEAVRRCNAYADAGADAVLIHSKARTRDEVLAFLDRWDDRVPVVIVPTTYPDWHIDEVRAAGVSVVIYANQGMRAALSAMRSTYRAIYEAGHTTALESEIASVRDIFDLQRMADWQKLDA	286	EDKLFPKMNS	unknown
**GCF03**								
Kitasatospora setae	DSM 43861, KM-6054	GCF_000269985.1	NC_016109.1	41	MLRVNHQSFRTVLAESDVVRVAGAHHALSGYLAQEAGFQAVWSSSFEVSASRCLPDASILSMTEYLEAAANIQKALSIPVVADCDTGYGDNLNVAHMVHEFEGAGITAVCIEDKLFPKMNSFVGSGQALLDTAGFAAKIRTAKNAQHSSDFFVIARTEALISGLDVTQALERCRAYADSGADAVLVHSKQRTKDQVMAFLDGWDGRVPVVIVPTTYPDWHIDEVRAAGVSVVIYANQGLRASVSSLRDAYGAIVRDGDTTGIEESIASVGDLFELQRLAEWQKLGA	286	EDKLFPKMNS	unknown
**GCF04**								
Kitasatospora purpeofusca	DSM 40283, NRRL B-1817	GCF_000718025.1	NZ_JODS01000049.1 Region on contig edge. supercluster	45	MSPTPHSYPSFRSVLADHPLVRIAGAHSALGGVIAEQVGFQAVWASSFEISAARCLPDASLLSMSEYLDAAAHIQKALSVPVVADCDTGFGNSMNVAYMVAEYEQAGITAVCMEDKIFPKMNSFAAADHSLLDVNAFAHKIETAKKSQRSEDFFVIARTEAMISGLGVDEALKRCAVYADAGADAVLVHSKQATKDEVLSFLDQWDDRLPVVVVPTTYPDWHADEARAAGVSAVIYANQGLRATITSLRSALGSIYETGHSAHLEDSIAGVKDVFALQRLDQWQEFTA	288	EDKIFPKMNS	unknown
Saccharothrix sp.	ST-888	GCF_000955975.1	NZ_JYJF01000012.1	50	MSLNIHPSHPSFRSVMAEHPLTRIAGAHSALGGVIAEQVGFQAVWASSFEISAARCLPDASLLSMTDYLEAAANIQKALTVPVVADCDTGFGNSMNVAYMVAEYERAGITAVCMEDKIFPKMNSFAAAAHTLLDAGDFAHKIETAKKAQRSEDFFVIARTEAMISGL	289	EDKIFPKMNS	phosphonothrixin[7]

Strain	strain designations	Genome accession	P-BGC accession	total no. BGCs	PepM sequence	PepM aa	PepM consensus motif (EDKX^{5}NS)	phosphonate product
			Region on contig edge.		GVDEALKRCTTYADAGADAVLVHSKKATKDEVLEFLEQWDNRLPVVVVPTTYPDWHADEAHAAGVSAVIYANQGLRATITSLRTALGSIYQTGHSAHLEDSIAEVKDVFALQRLGQWQEFTA			
Streptomyces sp.	WM4235	GCF_001279725.1	NZ_LGDE01000429.1 Region on contig edge.	40	MIRNSYPSFRQVLADNPLTKIAGAHSSLGGRIAEQSGFQAVWASSFEISAARCLPDASLLSMTDYLEAAANIQKALSVPVVADCDTGFGNSMNVAYMVAEYESAGITAVSIEDKIFPKMNSFAAADHTLLDVSAFAHKIETAKKAQRTDDFFVIARTEAMISGLGVDEALKRCNAYADAGADAVLVHSKQKTKDQVLEFLAHWDDRLPVVVVPTTYPDWHADDARAAGVSAVIYANQGLRATITSLRSTYRSIYETGHTSHLEDSISGVKDVFDLQQLDEWQQLGA	286	EDKIFPKMNS	phosphonothrixin[8]
Streptomyces sp.	TUE 21470	(N/A)	(N/A) supercluster	42	MSPTPHSYPSFRSVLADNPLVRIAGAHSALGGVIAEQVGFQAVWASSFEISAARCLPDASLLSMSEYLDAAAHIQKALSVPVVADCDTGFGNSMNVAYMVAEYEQAGITAVCMEDKIFPKMNSFAAADHSLLDVNAFAHKIETAKKSQRSEDFFVIARTEAMISGLGVDEALKRCAVYADAGADAVLVHSKQATKDEVLSFLDQWDDRLPVVVVPTTYPDWHADEARAAGVSAVIYANQGLRATITSLRSALGSIYETGHSAHLEDSIAGVKDVFALQRLDQWQEFTA	288	EDKIFPKMNS	unknown
**GCF05**								
Streptomyces aureus	NRRL B-2808	GCF_001418135.1	KC733813.1 Region on contig edge.	(N/A)	MTASRPPQQDRNDPPDGAARRLRAALAAPRLTRAMGAHSPLSARLAEEAGFDVVWSSGLEISAAAGVPDANILAMGECLDAAAGLASSVDLPVLADCDSGFGNVNNVIHMVRSYEARGVAGVCIEDKQFPKLNSFVEGNQDLAPLDDFAAKIRGASETRTDMVVVARLEALISGQGMAEALRRADVYERAGADALLIHSKRKDPEEVFAFREAYRGDLPVIVVPTTYNQVTVEELEQRGFAMAIYANQALRSSIRAMRETLTRIMRDGTTLNVEPELAPLKEIFDLQRMPQMLEQQERYESLGRELAEAAQ	311	EDKQFPKLNS	phosacetamycin[9]
Streptomyces sp.	A41	(N/A)	(N/A)	37	MTESRSPQQNERTPSAAQRLRAALATPRLTRAMGSHSPLSARLAEEAGFDVVWSSGLEISATAGVPDANILAMGECLDSAAGLASAVDIPVLADCDSGFGNVNNVIHMVRAYESRGMAGVCIEDKQFPKLNSFIEGNQDLAPLDDFAGKIRAATETRTDMVVVARLEALISGQGMAEALRRADVYERAGADALLIHSKRKDPEEVFAFREAYRGHLPVIVVPTTYNQVTVEELEQRGFAMAIYANQALRSSIRAMRETLTQIMRDGTTRNVEPDIAPLKEIFDLQRMPQMLEQQERYESVGRELAEAAK	309	EDKQFPKLNS	unknown
Streptomyces sp.	A4-2	(N/A)	(N/A)	39	MTESRSPQQNERTPSAAQRLRAALATPRLTRAMGSHSPLSARLAEEAGFDVVWSSGLEISATAGVPDANILAMGECLDSAAGLASAVDIPVLADCDSGFGNVNNVIHMVRAYESRGMAGVCIEDKQFPKLNSFIEGNQDLAPLDDFAGKIRAATETRTDMVVVARLEALISGQGMAEALRRADVYERAGADALLIHSKRKDPEEVFAFREAYRGHLPVIVVPTTYNQVTVEELEQRGFAMAIYANQALRSSIRAMRETLTQIMRDGTTRNVEPDIAPLKEIFDLQRMPQMLEQQERYESVGRELAEAAK	309	EDKQFPKLNS	unknown
Streptomyces sp.	TUE 3678	(N/A)	(N/A)	36	MNKPNSQPSPAEALRAALHAERPARAMGAHSPLSARLAEEAGFEVIWSSGLEISAAAGVPDANILAMPQCLEAAASLAESVSVPVLADCDSGFGNVNNVIHMVRAYEARGVAGVCIEDKQFPKLNSFIEGNQDLAPVDDFAAKIRAATEVRRDLVVVARLEALISGQGMAEALRRADVYERAGADALLIHSKLADPGEVFAFREAYTGDLPVIAIPTTYHQVTIEDLRERGFAMAIYANQALRSSLRAMRATLTKIIQDGSTHGVEE	303	EDKQFPKLNS	unknown

Strain	strain designations	Genome accession	P-BGC accession	total no. BGCs	PepM sequence	PepM aa	PepM consensus motif (EDKX^5NS)	phosphonate product
					DIAPLKEIFDLQRMPQMLEQQARYEALGRDLAEAGR			
**GCF06**								
Streptomyces kutzneri	DSM 40907	GCF_032248635.1	NZ_JASTTI010000003.1 Region on contig edge.	34	MFKPVSYPSFRSVLAQRRALSQAVRVAGAHDALGGRLAQAAGFDAVWASSLEVSAARCLPDASVLTMTEYLEAAAHTQKALGIPVVADVDTGYGNNLNVAHMVHEYEAAGITAVCMEDKLFPKMNSFVATSHTLLDTEEYCSKVRVAKNAQRGEDFFFIARTEALIDGLGVEVALERCHAYVDAGADAVLIHSKSRTKDQVLEFLQGWRDRAPVVVVPTTYPDWHADDAVKDGVSTIIYANQGLRATVSALRDTYASILSHGETTRLEDKIASVKDVFALQRLSDWQELESA	292	EDKLFPKMNS	phosphonoalamide A-D[10]
Streptomyces resistomycificus	DSM 40133	GCF_001514265.1	NZ_KQ949005.1	30	MLKSALHPSFRSVLAARRAAGSAVRVAGAHDALGGRLAEAAGFDAVWASSLEVSAARCLPDASVLTMTEYLEAAANTQKALGIPVVADVDTGYGNNLNVAHMVREYEAAGITAVAIEDKQFPKMNSFVATSHTLLDTDTFASKLRVAKQAQRTEDFFVIARTEALIDGLGVEVALERCHAYVDAGADGVLVHSKKPTNEQVLAFLAEWQDRAPVVVVPTTYPDWHIDDAVKAGVSTVIYANQGLRATIRALRETFSTIVDQGATTELEDRIASVKDIFALQGLAEWQELESA	292	EDKQFPKMNS	unknown
Streptomyces sp.	B-2790	GCF_041695405.1	NZ_JBAFSW010000002.1 Region on contig edge.	47	MLKSTPHPSFRTVLTARRAAGSAVRVAGAHDALGGRLAEAAGFDAVWASSLEVSAARCLPDASVLTMTEYLEAAAHTQKALGIPVVADVDTGYGNNLNVAHMVREYEAAGITAVAIEDKQFPKMNSFVATSHTLLDTETFSSKLRVAKQAQRTEDFFVIARTEALIDGLGVEVALERCHAYVDAGADGVLVHSKKPTNEQVLAFLAEWGDRAPVVVVPTTYPDWHIDDAVKAGVSTVIYANQGLRATIRALRETYGTIVERGDTTELEDRIASVKDIFALQSLTDWQELESA	292	EDKQFPKMNS	phosphonoalamide A-D[11]
Streptomyces badungensis	DSM 114471, 195335CR	(N/A)	(N/A)	30	MFKPVSYPSFRTVLGERRAAAKAVRVAGAHDALGGRLAQSAGFDAVWASSLEVSAARCLPDASVLTMTEYLEAAANTQKALGIPVVADVDTGYGNNLNVAHMVHEYEAAGITAVCMEDKLFPKMNSFVATAHTLLDTDAFCSKIRVAKNAQRGEDFFFIARTEALIDGLGVEVALERCHAYVDAGADAVLIHSKSRTKDQVLEFLREWKDRAPVVVVPTTYPDWHADDAFKDGVSTIIYANQGLRATVSALRDTYASILSHGDTTHLEDKIASVKDVFALQRLSDWQELETA	292	EDKLFPKMNS	unknown
**GCF07**								
Salinispora pacifica	DSM 45543, CNS-863	GCF_000383995.1	NZ_KB913022.1	24	MSDSNVLVVRTGQSKAAALRALLSADHPVRAVGAHDALTAKLVEQAGFDAVWSSSFEISASHGLPDASIVTMSQYLASSEAMDLGVTIPVIADCDSGYGGPLHTAYAVQRFERAGIAAVCIEDKLFPKMNSFADVTQELVTAEEFAMKIKAAKEVQNDPDFMVIARTEALIAGLSITEALDRGHAYVEAGADAVLVHSKSKRPDEVLEFASRWDSDVPLVAVPTTYSSISEAALGAAGFRIVIYANQGLRAAVRGVQETLQELSRAGCAQAVSERIAPMHEVFALQGMRAGFRDKP	296	EDKLFPKMNS	unknown
Streptomyces chrestomyceticus	DSM 40545, NBRC 13444	GCF_003865135.1	NZ_BHZC01000001.1	45	MSENATTTNKSARLRELLAGPRPVAAVGAHDGLSAKLVEQAGFDAVWCSSFEVSASYGLPDASLVTMTQFLAAAEAMDAIIDIPVIADCDTGFGGPLNVAFAVERYERAGIAAMCIEDKLFPKINSFADAGQDLLPTKEFALKIEAGKQTQKDERFLLIARTEALISGQGVPEALERAHAYADAGADAVLVHSKSRRPDDILELGDAWDRDVPLVAVPTTYASVEEKALFDAGYRLVIYANQGMRAAVKNMREVLGKLRVEGRAESVDADIATMPEIFALQGMTAAFRTAP	291	EDKLFPKINS	unknown

Strain	strain designations	Genome accession	P-BGC accession	total no. BGCs	PepM sequence	PepM aa	PepM consensus motif (EDKX^{5}NS)	phosphonate product
Streptomyces monomycini	DSM 41801, NRRL B-24309	GCF_000715845.1	NZ_KL571064.1 Region on contig edge.	45	MSENTTTNKSALLRELLAGPRPVAAVGAHDGLS<br>AKLVEQAGFDAVWCSSFEVSASYGLPDASLVTM<br>TQFLAAAEAMDAIIDIPVIADCDTGFGGPLNVAF<br>AVERYERAGIAAMCIEDKLFPKINSFADAGQDLL<br>PTKEFALKIEAGKQTQKDERFLLIARTEALISGQG<br>VPEALERAHAYADAGADAVLVHSKSRRPDDILEL<br>GDAWDRDVPLVAVPTTYASVEEKALFDAGYRLV<br>IYANQGMRAAVKSVREVLGKLRAEGRAEAVDA<br>DIATMPEIFALQGMTAAFRTAP	290	EDKLFPKINS	argolaphos[6]
**GCF08**								
Streptomyces alboniger	DSM 40043, ATCC 12461	GCF_008704395.1	NZ_CP023695.1 supercluster	30	MTVVSNSYVETSSRALVLRDAFESGRLVKVAGA<br>HDGLSARLAMEAGFDAVWASGLEISAAQGLPD<br>VSLLGMAEYLAGATAMQQAVSIPVVADCDTGF<br>GGSLNAAYTMRRYEGAGVAGICIEDKIFPKRNSF<br>LDAGQKLLETDEFGSKLEAAKKAQARPETLLIART<br>EAFICGMGVDEALRRCHQYVDAGADAVLVHSKS<br>AEAGEVVSFMRQWQHRAPVVIVPTTYADFSVQ<br>EAQEAGISMVIYANQGMRASVKAVRDTWAVVL<br>AEGSTASVEPHIATVKDIFSLSGIDHWLGDDK	295	EDKIFPKRNS	unknown
Streptomyces luridus	NRRL 15101	(N/A)	GU199252 Region on contig edge.	(N/A)	MTIASYGPSTLPGSRAATLRDAFANGRLVRVAG<br>AHDGLSARLAEEAGFDSVWASGLEISAAHGLPD<br>VSLLGMAEYLSAAVAMQRSVSVPVVADCDTGF<br>GGVLNAAYTMMRYEEAGVAGVCIEDKIFPKRNS<br>FVNSGQKLLDVDEFGRKLEAAKRAQVSSDTVLIA<br>RTEAFICGLGLQEALTRCHHYVDAGADAVLVHSK<br>AVDSGEVVSFMKEWNHRAPVVVVPTTYADFSA<br>AEAQEAGISMVIYANQGMRAAITAIRSTWATVL<br>AEGSTAPVEPRIASVKDIFAISGMDQWLDLDR	296	EDKIFPKRNS	dehydrophos[12]
Streptomyces sp.	TUE 3997	(N/A)	(N/A) supercluster	37	MTITSSESSTTRTKSTVSSPRAAALREAFADARLV<br>RTAGAHDGLSARLAEEAGFDAVWASGLEISAAN<br>GLPDVSLLGMAEYLAGAVAIQRSVSLPVIADCDT<br>GFGGVLNAAYTMMRYEEAGIAGICIEDKIFPKRN<br>SFVNAGQKLLAADEFGRKLQAAKRAQLSADTVLI<br>ARTEAFICGLGLEEALSRCHLYVDAGADAVLVHS<br>KAVDSTEIVSFMREWDHRAPVVIVPTTYADFSAT<br>QAREAGIAMVIYANHGMRAAITAIRDTWADVL<br>AKGSTADLEPRIATVKDIFALSGMDQWLETDR	302	EDKIFPKRNS	unknown
**GCF09**								
Kitasatospora fiedleri	DSM 114396^T, TUE 4103	GCF_948472415.1	NZ_OX419519.1	25	MSKSRTLRDGLNGSEVIRLVGAHSALSAKLGQD<br>AGFEAIWASGLEISASRALPDANVLSMSECLEAA<br>AQIADAVDVPVLADCDSGFGGVGNVAHMVRSY<br>QARGLAGVCIEDKQFPKLNSFVEGHQDLAPIGDF<br>AAKITAAKEARTGEDFVVVARIEAFIAGAGLDEAL<br>RRATVYEAAGADALLIHSKLSTPEEVFAFRAAYTG<br>ALPVIVVPTTYPQVTAAELTERRFGGVIYANQGL<br>RAAISAMRDVLGQIGAAGSTHAVEGSIATLKDVF<br>ALQNVDALLARQERHDALTAQYAALG	297	EDKQFPKLNS	unknown
Streptomyces sp.	31A4	(N/A)	KF386877.1 Region on contig edge.	(N/A)	MSNTRTLRDGLNGSAITRLMGAHSALSAKLGEE<br>AGFEAIWASGLEISAARALPDANILSMAECLQAA<br>TEIAGAVDIPVLADCDSGFGGVGNVVHMVRSYE<br>ASGVAGVCIEDKQFPKLNSFVEGNQDLAPVGDF<br>AAKITAAKEARRDEDFVVVARIEALIAGAGMEEA<br>LRRARVYEAAGADALLIHSKRSTPEEIFAFRAAYD<br>GALPLIVVPTTYPQVTAAELAERGFGAVIYANQG<br>LRASISAMRDVLARIDAAGSTYGVEESIAGLKDVF<br>ALQKVDELLARQERHDALTAAYASLG	297	EDKQFPKLNS	unknown

Strain	strain designations	Genome accession	P-BGC accession	total no. BGCs	PepM sequence	PepM aa	PepM consensus motif (EDKX^5NS)	phosphonate product
**GCF10**								
Streptomyces viridochromo-genes	DSM 40736, TUE 494	GCF_000158955.1	NZ_GG657757.1 supercluster	33	MNATERPGSDGTGSPESVGSRLKNLLHGPGTCQLMGVHDGLSARIAVAEGFEALWASGLCMSTARGVRDSDEASWTELLTLVGTMTEAAPGAPVLVDGDTGYGNFNTARRFAARAERVGAAGVCFEDKVFPKMNSFFGDGHQLAPIGEFSGKIKACKDTQRDPGFVVVARTEALISNLPMEEALTRAHAYVEAGADGLFIHSRMSTPQQIAEFMRQWDGSAPILIAPTTYHRPSLDDFAALGIAGCIWANHSMRAAFSAMRDVCQQIRADRGIFGVEERVAPLKEIFGLLDYESLEQDENRYTQAPDLAPVQG	313	EDKVFPKMNS	phosphinothricin tripeptide[13,14]
Streptomyces mooreae	DSM 41527, ATCC 21705	GCF_031845725.1	NZ_JAVRFE010000017.1 supercluster	39	MNATEQAASGDRGTTRSAGGRLRYLLHAPGACQLMGVHDGLSARIAVAEGFEALWASGLCMSTARGVRDSDEASWTELLTLVGTMTDAVPGVPVLVDGDTGYGNFNTARRFAGRAERVGAAGVCFEDKVFPKMNSFFGDGHQLAPVAEFCGKIRACKDAQRDPDFVVVARTEALISKLPMEEALDRAAAYAEAGADALFIHSRMNTPQQIATFMERWEGSTPVLIAPTTYHTPSVDDFAALGIAGCIWANHSMRAAFAAMRDVCQRIRTDRGIYGIEDQVAPLKEIFGLFDYEGLEKDENCYTQAPDLAAVQG	313	EDKVFPKMNS	phosphinothricin tripeptide[15]
**GCF11**								
Goodfellowiella coeruleoviolacea	DSM 43935	GCF_024171785.1	NZ_JAMTCK010000002.1	51	MDSTRPAQPPSRSARLRAMLAAPEISFLMEAHNGLSARIAQHAGFPGIWASGFAISTALGVRDSNELSTKELLDTVAFMVDATTVPIVVDGDTGYGNFNNARRLVRQLGRLGVSAVCLEDKLFPKTNSFLGTGQPLADEREFCGKIRACKDSQLDAEFCVVARVEALVSGRGLTEALHRAESYRRAGADAVFIHSKQQDGREVLDFAREWAGRCPLVITPTTYHGVGVDAFERAGVAMLIWANQNMRAAVLAMRQVCQSIAANRGLTAVEPALVSVQEVFDFLDYDELDAATLRYSSWHGDETTAGAGP	309	EDKLFPKTNS	unknown
Streptomyces rimosus	DSM 40260, ATCC 10970	GCF_000331185.2	NZ_CP048261.1 supercluster	46	MGDTGGSIQREDLMHHNHHTPTTAKTTMMRELLRSRSLGFLMEAHNGLSARIVEDVGFPGIWASGFSISTALGVRDSNEVSAGEVLDTVAYMADATRIPIMVDGDTGYGNFNNARRFVRQLCRLGVAGVCIEDKLFPKTNSFIGDRQPLADTDEFRGKIAACKDSQTDDDFCVIARVEALVSGFSLAEALGRAEAYRQAGADAVFIHSKRKDGKEVLAFAREWGRRSPLVITPTTYHRSVGVDTFERAGISTLIWANQNMRASMRAMREVSEEVFKKRRVSDVEQDLVPVQEVFDFLDYGELDDASRRYLPADPAPASRRDAS	323	EDKLFPKTNS	unknown
**GCF12**								
Pantoea ananatis (1)	LMG 5342(-1)	GCF_000283875.1	NC_016816.1	9	MIKKLIAEKGTLIFIEAHNPLSALIASKAEQTNSEGRIVKFDGIWSSSLTDSASRGIPDNETLALSSRLENIADIRNVTDMPIIMDADTGGKPEHFSYYVKRMINNGVNGVIIEDKTGLKKNSLFGTEVEQTLADINDFSEKIKRGKSAVYIDDFMIIARLESLIAGFDVEHALERADAYVEAGADGIMIHSCKKTPDEVFLFSTKFRKKYPSVPLICVPTTYSATSNRELSEAGFNVIIYANHMLRAAYKAMENVSKEILRYGRTAEIEKSCMSVKEIISLIP	284	EDKTGLKKNS	pantaphos[16]
Streptomyces thermoviolaceus	DSM 116655, SFG18	(N/A)	(N/A)	23	MSISTIGNETRLTALRESLSRGEPVRIIEVHSPLAAVIAEHAQGKGPDRREFHGFWSSSLTDSALRGLPDIELLDMEVRLSWIDQIFSVSTLPLVMDGDTGGQTLHFEYLVKAMERRGVSAVVIEDKCGEKRNSLL	306	EDKCGEKRNS	unknown

Strain	strain designations	Genome accession	P-BGC accession	total no. BGCs	PepM sequence	PepM aa	PepM consensus motif (EDKX^5NS)	phosphonate product
					PGEGLHTMAPVDEFCDKISRGKAAQSSDAFMIIARLEGLITGLSRQEILDRAGAYVAAGADGLVLHSRSSDEAPLLDLARELRRRHPDTPLVAIPTAYPSVTEEELHAAGFNMIIYANQMLRAATRAMEEVSHRILDHGRALEAGEVCIETSKLLTMPDDKRPIVRAPRN			
**GCF13**								
Streptomyces doudnae	DSM 41981	GCA_031845505.1	NZ_JAVRES010000001.1	38	MTATGRTVGRTAALRHLLERAELTFLMEAHNGLSAKLVEQAGFEGIWASGLSIAAALGVRDSNEASWTQVLEVAEFMSDATRIPILLDGDTGYGNFNSVRRLVRKLEQRGVAGVCVEDKLFPKTNSFVRGGAQPLADAEEFAGKIGAAKASQQDQDFVVVARTEALIAGLGLAEALRRAELYRRAGADAILVHSARADAGEVRAFKAEWGDRLPVVIVPTAYYRTPTEVFAEDGISTVVWANHLMRSALRAMQITASTIFAEQHLRNVEGTVAPLAEVFALQGEDELAEAEARWLPRRNTPSR	303	EDKLFPKTNS	unknown
Streptomyces hintoniae	DSM 41014	GCA_031845745.1	NZ_JAVRFF010000014.1	41	MTAPGRTTSRAAALRHLLDRPELSFLMEAHNGLSAKLVERAGFEGIWASGLSIAAALGVRDSNEASWTQVLEVAEFMSDATRIPILLDGDTGYGNFNSVRRLVRKLEQRGVAGVCVEDKLFPKMNSFVRGGAQPLADAEEFAGKIGAAKSAQHDPDFVVVARTEALIAGQGLAEALRRAELYRCAGADAILVHSARSDADEVRAFKAEWGDRLPVVIVPTKYYRTPTEVFAEDGFSTVVWANHLMRSALRAMQITASTIFAEQHLRNVEGAVAPLAEVFEIQGEDELAEAEQRWLPQRNTPRR	303	EDKLFPKMNS	unknown
**GCF14**								
Amycolatopsis xylanica	DSM 45285	GCF_900107045.1	NZ_FNON01000001.1	45	MDAFSPALRELLDRPGPVRAAGAHNPLGARLAERAGFDAVWSSGLEISASQGLPDADILTMTELLAVAQSMAAAVSVPVIADCDAGYGNASNVMHLVRRYEAAGVAAVCIEDKTFPKVNSFVPGRQTLLPAEDFCNKIAAAKAAQSRPDLVVIARLEALVAGWGLDEALRRGEAYAEAGADMVLIHDKGKSPQPVLEFLQRWRRPVPVAVVPTTYHTVTADELSDAGAKLVIYANHGLRAAITAITSAFDTILRDGRTTGIEDRISPLSTVFDLQGMRQFIEAERRFVAERGPVEGVA	298	EDKTFPKVNS	unknown
Kitasatospora cheerisanensis	DSM 101999, KCTC 2395	GCF_000696185.1	NZ_KK853997.1	34	MRAPSRRPAARLRALLSGPEPIRLVGAHNALGARLAERAGFDGIWASGLEVSASHGVPDADILTMSELLATAQSMARAVEVPVVADCDTGYGNAINVMNTVRRYEAAGIAGVCIEDKVFPKMNSFVSGRQQLAQLGEFCGKVEAAKAAQEDPDFVVIARVEALIANRGMEEAVRRARAYADAGADAILIHDKDRSPASIFEFIDRWDFRKPLVVVPTTYYSVTVDELAAAGVKMVIYANQGLRASIAAVSRTFEEILSAGTSAPVEERIAPLDLVFDLQGVPELKRNEELYLRSDAPVEEGAPHRATPDPAPADAAALVASHAN	324	EDKVFPKMNS	unknown
**GCF15**								
Streptomyces aureocirculatus	DSM 40386, ISP-5386	GCF_000720475.1	NZ_JOAP01000003.1	45	MSQGDLVRVAGAHDAMGATLAQQAGFQAVWSSSLEVSASRCLPDASVLTMTEYLAAAANMQKAIDIPVVADVDTGYGNNLNVAHMVHEYEAAGITAVAIEDKLYPKVNSFAEVAQTLLPLTDFAQKIETAKNAQRGEDFYVIARTEALIDGLGVEEALKRCTAYADAGADAILIHSKKKDEGEIVEFLDGWDGRKPVVIVPTTYPQWSAAEATKHGVSVVIYANQGLRATVQ	274	EDKLYPKVNS	unknown

Strain	strain designations	Genome accession	P-BGC accession	total no. BGCs	PepM sequence	PepM aa	PepM consensus motif (EDKX^{5}NS)	phosphonate product
					ALRTTFGTIYRDGTSLGVESEIAPVSDVFALQRLQDWLALEA			
Streptomyces silvensis	ATCC 53525	GCF_001482415.1	NZ_LOCL01000042.1	51	MTSHRASFRAVMSQGDLVRVAGAHDAMGATLAQQAGFQAVWSSSLEVSASRCLPDASVLTMTEYLAAAANMQKAIDIPVVADVDTGYGNNLNVAHMVHEYEAAGITAVAMEDKLYPKVNSFAEAAQTLLPLADFAQKIETAKNAQRGEDFYVIARTEALIDGLGVEEALKRCTAYADAGADAILIHSKKKDEGEIVEFLDGWDGRKPVVIVPTTYPQWSAAEATKHGVSVVIYANQGLRATVQALRTTFGTIYRDGTSLGVESEIAPVSDVFALQRLQDWLALEA	285	EDKLYPKVNS	unknown
**GCF16**								
Nonomuraea sp.	G22-1	(N/A)	(N/A)	38	MSVYGAQRRDRPTAEPAPSPGARLRALLGGSEPAVLMGAHDGLSARVAAEAGFPALWASGLCISTALGVRDSDEASWTELLEMAARVVAASGLPVLVDGDTGHGNFNTARRFTARVEQIGGAGVCLEDKLFPKMNSFVGDGHELTKIGEFCGKIAACRDALADPDFVIVARTEALIAGAGLDEALRRGEAYRRAGADALFIHSRQSTVSEIAQFAREWQSRLPLVIAPTTYHTTPMEEYVKLGISGVIWANQSMRASLAAMRKACESLRRDGPAAVEDGISSLEELFSLMRYQELAEDEKRYGDQAPGIAELMATRKPGS	320	EDKLFPKMNS	unknown
Nonomuraea candida	DSM 45086, NRRL B-24552	GCF_000725485.1	NZ_JOAG01000015.1	34	MSVYGAQRRDRPTAEPAPSPGARLRALLGGSEPAVLMGAHDGLSARVAAEAGFPALWASGLCISTALGVRDSDEASWTELLEMAARVVAASGLPVLVDGDTGHGNFNTARRFTARVEQIGGAGVCLEDKLFPKMNSFVGDGHELTKIGEFCGKIAACRDALADPDFVIVARTEALIAGAGLDEALRRGEAYRRAGADALFIHSRQSTVSEIAQFAREWQSRLPLVIAPTTYHTTPMEEYVKLGISGVIWANQSMRASLAAMRKACESLRRDGPAAVEDGISSLEELFSLMRYQELAEDEKRYGDQAPGIAELVATRKPGS	320	EDKLFPKMNS	2-phosphinomethyl-malic acid + des-methylphosphino-thricin, possibly other H-phos-phinates[6]
**GCF17**								
Streptomyces fradiae	HZ1738	(N/A)	EU924263.1 Region on contig edge.	(N/A)	MQRPIVYVGMSADLIHPGHINILSRAAELGDITIGLLTDAAIASYKRLPHMTYEQRKAVVENLKGVASVVPQRTLDYAENLRTVRPDFVVHGDDWQTGVQRHTRERVIEVLSEWGGKLIEIPYTPGISSTHLHSSVKEVGTTPNVRLSRLRRLLDSKEIVRILEVHNGLTGLIIENSKVTVDNQAREFDGMWSSSLTDSLARGKPDTEAVDVSSRLQMVNELFEVTTKPLVFDGDTGGKPEHFGFTVRSLERLGVSAVIVEDKEGLKRNSLFGTDVPQTQSSVEDFSARIRIGKRAQITDDFMVIARIESLILEKGMADAVHRAEAYVDAGADGIMIHSRQSDPAEIFEFCRYFDKLPRRVPLVVVPTSYSSVRESELADAGVNMVIYANHLMRAVYPQVTKVVQSILQHGRAHEAESMLASIKDALSIIPENSG	435	EDKEGLKRNS	fosfomycin[17]
Streptomyces wedmorensis	DSM 41676, ATCC 21239, NRRL 426	GCF_000716445.1	NZ_JNWK01000026.1 supercluster	37	MQRPIVYVGMSADLIHPGHINILSRAAELGDITIGLLTDAAIASYKRLPHMTYEQRKAVVENLKGVASVVPQRTLDYAENLRTVRPDFVVHGDDWQTGVQRHTRERVIEVLSEWGGKLVEIPYTPGISSTRLHSSVKEVGTTPNVRLSRLRRLLDSKDIVRILEVHNGLTGLIIENSKVTVDNQAREFDGMWSSSLTDSLARGKPDTEAVDVSSRLQMVNELFEVTTKPLVFDGDTGGKPEHFGFTVRSLERLGVSAVIVEDKEGLKRNSLFGTDVPQTQSSVEDFSERIRIGKRAQITDDFMVIARIESLILEKGMADAVHRAEAYVDAGADGIMIHSRQSDPAEIFEFCRYFDKLPRRVPLVVVPTSYSSVRESEL	435	EDKEGLKRNS	fosfomycin[18,19]

Strain	strain designations	Genome accession	P-BGC accession	total no. BGCs	PepM sequence	PepM aa	PepM consensus motif ($EDKX^5NS$)	phosphonate product
					ADAGVNMVIYANHLMRAVYPQVTKVVQSILQHGRAHEAESMLASIKDALSIIPENAG			
**Singletons**								
Actinoalloteichus cyanogriseus	DSM 43889	GCF_000429185.2	NZ_AUBJ02000001.1	22	MIREKELTYLMEAHDGLSARIAEVEGFQAIWASGLSMSTALGVRDSDEASWSQLLGVVEAMVEATTVPIVVDGDTGYGNFNTARRFVAKAERLGVAGVCLEDKIFPKMNSFVGDSHTLAEVSDFTAKIRACKDSQRSSDFVLVARVEALIAGRGLPEALDRAHAYHEAGADAIFIHSRRSDATEILDFCREWGERLPLVIAPTTYASTPSSEFQAAGVSAVIWANHSMRAAVSAMRRVCREIFAKQSVHRTESELASLAEVFELMEYSEIDEAERRAYRV	280	EDKIFPKMNS	unknown
Actinokineospora auranticolor	DSM 44650	GCF_041897805.1	NZ_CP154825.1	35	MTEDTRGQVRQVETRAAALREAFAEGRLLRVAGAHDGLSARLVAEAGFDAVWASGLEISAAHGLPDISLLGMAEFLAAAATMQSATAVPVVADCDTGFGGDRNAAFTMMRYENVGIAAVCIEDKVFPKLNSFLGAGQELLATEDFGAKLRAAKQAQHDPDTVLIARTEAFICGYGVEEALARCHHYVDVGADAVLVHSKAADSSEVVSFTRRWQRRAPVVVVPTTYTAFSVAEAQEAGVAMVIYANQGMRATVRAVRDAWAAVLADGSTVGIESTIASVKDIFELSGMSQWLGRG	295	EDKVFPKLNS	unknown
Actinopolyspora mzabensis	DSM 45460	GCF_900101095.1	NZ_FNFM01000005.1	21	MFLNHHYVLETVLSISTRTESPESARQPEAGSGRPGRFRRMLASPELSFAMEAHDGLSARIAQAEGFRALWASGLSISTAMGLRDNNEASWSQFLQVVELMADATSVPIVVDGDSGHGNFNSARRFFRKAEQVGAAAVSLEDKIFPKMNSFVGTEHELAPAEEFCAKIRACKDSQQDPEFCLIARTESLIAGVGMREAIDRAEQYRLAGADAIFIHSKKQVSDEIVEFSQEWGERLPLVIAPTTYAATTPTDIFRRCGISTVIWANHSMRAAFAAIRQTCREIRDTESVSHLETLAPLSNVFDLLDYDELERAESRFFTDHPMNG	325	EDKIFPKMNS	unknown
Bacillus spizizeni	ATCC 6633, NRS-231	GCF_000177595.1	NZ_ADGS01000025.1	11	MKAKKLRELLYSNQVVRVMGAHNGLSAKLAEQAGFHAIWASGLEISASYAVPDANILTMTENLQAAVVMNESTSIPIICDCDSGYGSVNNVIRMVKEYERNGIAGICIEDKQFPKLNSFVKGSQKLADIDEFSNKIRAAKDVQKNPDFVVIARIEALIAGQGMDEALNRAYAYEAAGADAILIHSKENQPNEIKEFVKQFTGAVPIVIVPTTYPHITVKEMELLGINMVIYANHGLRSSIKAMQETFSQILLDGNTVGVEDNIVSMKTVFELQGMYDMRKQEDMYNSGTSVISTIK	296	EDKQFPKLNS	rhizocitins[20]
Bacillus velezensis	AA3, NRRL B-41580	GCF_001461825.1	NZ_LLZC01000022.1	19	MVKNRKSSIFRDALDSKSLVKVAGAHDGLSAKLAEKNGFNAVWASGLGISAVQTVPDASILTMTEFLEAAVIMNESCNLPVIADCDSGYGNIHNVTRMIKKYEAAGIAGVCIEDKVYPKLNSFDDRQQILVSTEEFCAKIRAAKMAQQNDDFVLIARVEALIAKLGQEEAYTRAKAYVHAGADAILIHSKEQSPDEIIEFVNNWDVDAPLVVVPTKYPTLSMEQLEKLGVKVSIYANQALRASVKAINDTFESIINNKSSLQIENDIVSVNEIFDIQDVPGMKQLERLIHRQPAN	295	EDKVYPKLNS	phosphonoalamide E + F[21]
Glycomyces sp.	NRRL B-16210	GCF_000719515.1	NZ_JOGR01000003.1 Region on contig edge.	11	MSADLIHPGHINILNRAAELGDVTIGLLTDAAIASYKRLPHMTFEQRKAVVANLKGVTAVIPQETLDYVPNLRSVKPDFVVHGDDWQTGVQRETRQRVVDALAEWGGELVEVPYTQGISSTQLNASVKEVGTTPDVRLTRLRRLIDAKPIVRVLEAHSGLTGLIIETAQAERGGLKVEFDAMWSSSLTDSTARGKPDIELVDLNSRLQTINDLFEVTTKPLIYDGDTGGKPEHFSYTVRSLERLGVSAVIIEDKEGLKRNSLFGTEVAQTQSTIEDFVHRIQVGKKAQVTDDFMIIARIESLILEMGMEDAVTRAKAYIEGGADGIMIHSRQKSPDEIFEFCERFAAFETKVPLVVVPTSYNQVTEEEFIERGVN	426	EDKEGLKRNS	phosphonoglycans (2-HEP containing polysaccharides)[22]

**Strain**	**strain designations**	**Genome accession**	**P-BGC accession**	**total no. BGCs**	**PepM sequence**	**PepM aa**	**PepM consensus motif (EDKX^{5}NS)**	**phosphonate product**
					VVIYANQLMRASYKAMSGVAKSILENARSAEVDSQIANIKEALAIIPENES			
Kibdelosporangium banguiense	DSM 46670	GCF_017876405.1	NZ_JAGINW010000001.1 supercluster	47	MTVDELSNKVTHSPGSRLLRILESPGVSTFLGVHDALSARIAADCGYPALWASGLGMSTALGLRDCDEASWTELLDITARMVEATNLPILVDGDTGYGNFNTARRFAMRAERAGAAGVCIEDKVFPKMNSFVGDGHALAPIPEFCGKIAACKDAVRDSSFVLVARVEALIAGAGMGVALERAEAYRAAGADAIFIHSRMPTVAQIAEFTLGWNDKLPLIIAPTTYHRTPIAEFERLGISGVIWANQAMRAAVAAVKRVCAALHDGPAGIDSSISSLPEVFSLLDYDALERDEQLYGSYEHRNPL	304	EDKVFPKMNS	unknown
Kitasatospora phosalacinea	DSM 43860, NRRL B-16230	GCF_000716545.1	NZ_JNWZ01000012.1 supercluster	24	MTSPTSLPDGGTAPSTPGQRLRHLLAQTEPVQLMGAHDGLSARIAAAEGFPALWASGLCMSTARGVRDSDEATWSELLDLAATMIEAAPGVPVLIDGDTGYGNFNTARRFAARAERIGAAGVCFEDKVFPKMNSFFGDGHALAPVPEFCGKIRACKDGQRDPGFLVVARTETLIAGRPVEEALERAEAYAEAGADALFIHSRKPTPVEIAGFLERYDGRLPVVIAPTTFHTPTVDEFGRLGVSGVIWANHSMRASFAAMRDVCQQIRSNRGISAVEGQVASLKEVFGLLEYEALEADEAAYTGALGGAAVAG	312	EDKVFPKMNS	phosalacine[23,24]
Lentzea sp.	TUE H45	(N/A)	(N/A) supercluster	35	MTKAAELRELLTRPAVSRIVGARDPLTARMVEEAGFDGVWVSSFELSATRALPDLGLLTMTECLDAAALIDGATVLPSLVDCDTGFGTAINLVRVVRLCEAAGIAGICVEDKVFPKRNSYLDGGQVLEDPDAFAQRVEAAVRSRRDQDFTVVARCEALIAGAGMDEAVRRAHHYVDAGADAVLVHSKRREPDEIVEFLHRWRRRAPVVVVPTTYYRWSLAEAQDAGVSLVIYANQALRASVLAVRTVLSEIHLRGDAATAEDSIAPVKEVFALTRTAEWEEWGA	284	EDKVFPKRNS	unknown
Nocardia mexicana	DSM 44952	GCF_003350525.1	NZ_QQAZ01000001.1	38	MTHTNGHGSKSAQLRKLLAGDRLARVVGVGDGLSALLASRHGFDALWGSGLSISAAHALPDASILTMTEFLQATAVIDAATPLPTIADCDTGFGDVNVVMRATREYERRGIAGICMEDKQFPKRNSFSGGQQLAPIAEFAQKIRAAKSVQVSPDFVVIARVESLIAETGMADALERGARYAQAGADALLIHSKSESPDEVLEFAAEWNRQSDPLPLVAVPTTYYSASVDELERGGFSMVIYANQSLRAVVRVVNDVFARLSSAASTAEMEEQLIPVVEVLDLIGMDSLLTSDVDG	295	EDKQFPKRNS	unknown
Nocardia pseudobrasiliensis	DSM 44290	GCF_003350585.1	NZ_QQBC01000006.1	54	MRENLPVRTRPPAAGVAGAQLRGLLAEPEPSYLMGAHDGLSARIAAEAGFPAIWASGLCMSTILGCRDNDEVSWSELLDLTARMVEAGGVPVLVDADTGYGNFNTARRFAARAQRIGAAGICVEDKVFPKMNSFFGEQHPLAPIGEFCAKIAACRDCVDDEFVIVARVEALIAGAGMAEALRRAEAYREAGADAIFIHSRQRTVEEIAEFTREWGARLPLVIAPTTYYTVPAQTFAELGIAAVIWANHAMRASVAAMREACRALRRDGAAAVEPVITPLDELFSLMGYPELEADQAHYSSLAQKWVDS	308	EDKVFPKMNS	unknown
Nocardia tenerifensis (1)	DSM 44704(-1), NBRC 101015	GCF_003202065.1	NZ_QKFIF01000021.1	47	MSENFPGRTGATAGLRLRGLLADPQPSYLMGAHDGLSARIAAEAGFTGVWASGLCMSTVLGARDSDEISWAELLDLVARIVEGGGLPVLVDADTGYGNFNTARRFAARAERIGAAGMCLEDKVFPKMNSYFGDSHPLAPVGDMCAKLAACRDQVDAEFVLVARTEALIAGAGLAEALRRAEAYRQAGADAIFIHSRRRTVDEIAEFTAEWGARLPLVIAPTTYHTVPRATFAELGIAAVIWANQSMRAAVAAMRQVCRSLLADGAAAVEPGIASLRELFDLMGYPELEADEAHYTALAQKWSDAKPVRVQSHSMSDEDVKHVS	323	EDKVFPKMNS	unknown

Strain	strain designations	Genome accession	P-BGC accession	total no. BGCs	PepM sequence	PepM aa	PepM consensus motif (EDKX^5NS)	phosphonate product
Nocardia tenerifensis (2)	DSM 44704(-2), NBRC 101015	GCF_003202065.1	NZ_QKFIF01000015.1 supercluster	47	MEKVRALASLFERAGLDFLMGAHDAVSAQIAQDAGFPGIWVSGLGLSATNGLRDSNELSWTQVMERIEMLSDRIAVPALVDMDTGYGDFNNVRLAVRRLRRIGAGGACIEDKLFPKTNSFLGDGQALAEPGEFCGRLKAAKDTAADVFLVARCEALVAGRPLAEALDRCGMYAESGADAVLIHSKKTNPDEILAFMAEWDGSAPVAVVPTKYADVPASVLEEAGVSVAIWANQSLRAAIAAMRRLCTQLAQQRTMRGLEGEIAELSQVFELANNAELDQAKTRYSRYVPPSREAVANNGARTRAAETVDDVLRLREAWSAVQSTEDPAETLLAHVRDTVPFYQGIGTQELSDLPVVDRTAYENDVAEFTSAASTGKYCLTSSGTTGNPLTVTLDDASWYAVNYHFFTQICDLAGLPPDGFRAGELAVLFVSNKPGRDTFVRPLPSLNYGLYARIQLDLSAKSTRTYSRLGAEILYGKPTYLLDLRAALIAQGFATPPWSLRLVLVSGEPLHADDRARLTDYYGAPVVDALASTEGGLIAATRPGEETYQVFPENVRLEVLTDDGVVRDSGVGELVLTNLVYRDTVFLRYRTGDRAELETDAGGSQRLRRLWGREPRTLSFGDRRLPTRDLTERFGSLPGMGDFQIVSRLDGALLRWMPDAGYQEPETLRRSLRAAVDELLPDQDVEFELCSRITPPGGKKRRFR	704	EDKLFPKTNS	unknown
Pantoea ananatis (2)	LMG 5342(-4)	GCF_000283875.1	NC_016816.1	9	MQKKIDRLGSVNSKNRILREMISSTELSFLMEAHNGLSAKIAQNSGFSGLWASGLTISASLGLSDRNEASWTQVLDVAEFMADHVDIPILLDGDTGFGSYLNVMRLVKKLCQKGISGVCLEDKVFPKMNSFIGEKQELIAIEDFCSKIKAAKDTQLDDDFVLVARTEALISGRGMAEALRRAEAYHEAGADAILIHSKKTDASEIVEFSNEWAERSPLVIVPTKYYSTPTELYRDLKISTVIWANHSLRASVVAIENTTKKIFETQSIKEVEQDIVSLQDLFLLTNEVNSAIAEKKYGL	299	EDKVFPKMNS	unkown
Pseudomonas syringae	PB-5123	(N/A)	JX102649 Region on contig edge.	(N/A)	MSSITHAEQFRRSLFNGKLDLILEAHNGLSARIVEEAGFSAIWASGLSISSALGLRDCNEASWSQVVEVTQSIHDAVTIPILFDGDSGFGNFNNVRHIVKRLSHYGIAGISLEDKLFPKMNSFITGAHALSPVEEFCGKIKAAQDSKCDRHFTVIARTEALIAGLGVEAALERAQHYHDAGADGIFIHSKTGDGLDILEFGRRWERRSPLVVAPTTYINTPLALLEEVGVSIYICANHLMRASIQAMRKAAGQIRKDNSISTINSDISSLTEVFSLLNYDELQAAERLYAR	291	EDKLFPKMNS	fosfomycin[25]
Saccharopoly-spora spinosa	DSM 44228, NRRL 18395	GCF_000194155.1	NZ_GL877879.1	38	MLDSPEMVFLMEAHDGLSARIAQSEGFDALWASGFSISTSLGLRDSDEASWSQLLSVVEYMVAGTSVPIVVDGDTGYGNFNTARRFLVSAERQGAAGVCFEDKVFPKMNSFVGDSHRLADIDEFAAKIAACSEARNDEDFCIIARTEALIAGRGVDEALKRAEAYRKAGATAIFVHSRRQVADEIEQFAAAWADRAPLVIAPTTYASTSADTFRELGISAVIWANHSMRAAVTAMRDVCRSIKASESVLSAEAKIASLKEIFEFMDYAEIEETERRLLLRGTSAADD	287	EDKVFPKMNS	unknown
Stackebrandtia nassauensis	DSM 44728, NRRL B-16338	GCF_000024545.1	NC_013947.1	18	MSSKPTVYVGMSADLVHPGHINILQRAAELGDVTIGLLTDAAIASYKRLPHMTYEQRKAVVENIKGVAAVVPQETLDYVENLEKLKPNFVVHGDDWKTGVQQQTRQRVIDALAQWNGELVEVGYTEGISSTQLNESVKEVGTTPNVRLSRLRRLIENKPIVRIMESHSPLTGLIIETTTAEHNGRKVEFDGMWSSSLTDSTARGKPDIELVDPASRMQGINDLFDVTTKPLIYDGDTGGKPEHFSYTVRSLERLGVSAIIIEDKEGLKRNSLFGTGAQQTQCSIEDMVHKIQVGKKAQVTKDFMVIARIESLILEQGMDDAVKRARAYIDEGGVDGIMIHSRQKTPDEVFEFCELFQKFPKRVPLVVVPTSYNTVVEDEFAKRGVNVVIYANQLMRASYKAMASVATSILENGRSAEVDSQIANIKEALAIIPENLA	437	EDKEGLKRNS	phosphonoglycans[22]

Strain	strain designations	Genome accession	P-BGC accession	total no. BGCs	PepM sequence	PepM aa	PepM consensus motif ($EDKX^5NS$)	phosphonate product
Streptomyces durhamensis	DSM 40539, NRRL B-3309	GCF_000725475.1	NZ_JNXR01000046.1 Region on contig edge.	43	MLKRSYPSFRSLVEKGELVRVAGAHDALGAVLAEQAGFDAVWSSSLEVSAARCLPDASVLTMTEYLDAAANMQKALGIPVIADCDTGYGNNLNVVHMVHEYEAAGITAVCMEDKLFPKLNSFASGDQTLLSTEAFCSKIRAAKGAQATGDFFVVARTEALINGLSVDVALERSHAYCDAGADAVLIHSKSKTKDQVEEFLSRWDGRRPVVIVPTTYPDWHVDDIAKAGVSVVIYANQGLRATVASLRETFESICRHGDSTDLEDRIAAVSDVFELQKLKEWQKLDA	286	EDKLFPKLNS	valinophos[6,26]
Streptomyces glauciniger	DSM 41867, CGMCC 4.1858	GCF_900188405.1	NZ_FZOF01000041.1 Region on contig edge. supercluster	35	MTSPQLTAPTMPRPKPGTGAAKLRALFMRPGITRIVGAHNPLGARLAERAGFDGVWSSGLEISASQGLPDTDILTMTELHTVAASLAAAVDIPVVADCDAGYGNAFNVMHMIRRYEASGIAAVSIEDKLFPKVNSFIPRRQKLASIEEFSGKLRAAKSVQEDPDFMVIARIEALIAGWDMEEALRRGEAYAAAGADAVLIHAKGSEAEPVLEFLSRWQPDLPVVVVPTTYHTVTADELSAAGAKMVIYANQGLRAGISAVSETFDTILRDGRTTALEDRIAPMKTVFDLQGMPQVMADEARFLPNSGKTDDQ	312	EDKLFPKVNS	unknown
Streptomyces iranensis	DSM 41954	GCF_042466515.1	NZ_CP136563.1 supercluster	70	MANGNLIRAAGAHDALGSALAQQAGFQAVWASSLEVSASRCLPDASVLTMTEYLAAAANMQKAVDIPVVADVDTGFGNNLNVAHMVREYEAAGITAVCIEDKVYPKVNSFAATDHELLPVGTFTRKLATAKAAQQDEDLYVIARTEALINNKGVDEALDRCYAYAEAGADAVLVHSKKKDQGEIVEFLDSWDGRSPVVIVPTTYPQWSADEAEKHGVSMVIYANQGLRATIQALRDAFRTIYDDGTSLGVENAIAPVSDIFALQKLDDWLALEA	274	EDKVYPKVNS	unknown
Streptomyces lavendulae	Fujisawa #8006	GCF_000715625.1	NZ_JNXL01000002.1 supercluster	26	MTAAATRASEGITDGTSLRDLFERPGVVRIAGAHNPLGARLAERAGFDGVWSSGLEVSASQGVPDTDILTMSELLAVAGSLASAVSVPVVADCDAGYGNAHNVMNMIRRYEAAGISAVSIEDKRFPKVNSFIPGRQELAPIGEFCGKLAAAKAAQRGSELMVIARIEALIAGWGMDEALLRGEAYADAGADAVLIHAKGSSPDPILEFLNRWRLPVPVVVVPTTYHTITAAELGEAGAKMVIYANHGLRAGITAVSRAFEAILREGRTTGIEEQIAPLATVFDLQGMPQQKQHEKLYITPYGTSPRAFMVPGEQQQTGLDQRADVLVCQTAELRRSGVEQVAVYAADQPPTRELQDVDVVAGVGTDAAAWVLSTPANYPGSTLVLPADVFLEAGPLRQLASNESDVAVLVDVSTRSGAARRPDAVGVSLSSSIRGGRRLTAGSSLVTGFGGSEVEAEFTGAAVFSARGFAALVDAAEKRRAEGSTATVVELLADVLLGGLQVHAIEVASGWTELRTADDLRYVGEAMATGGAER	534	EDKIFPKRNS	fosmidomycin[27,28]
Streptomyces mutomycini	DSM 41691, NRRL B-65393	GCF_001700505.1	NZ_MAPV01000141.1 Region on contig edge.	54	MTTSSARLRELLTAPQAARAAGAHDGLTARLVEEAGFDVVWASSFETSASRALPDMSLLTMTDYLQAASWMVQSTSLPVLADCDTGFGNRLNIAYTVRQYEAAGVAGICLEDKVFPKKNSFLAVGQTLEDPAEFAARIRVAKQAQHNPEFVVVARVEALIAGTGMADALARAHQYADAGADAILIHSKSQEPHEIQEFLEQWQRRTPVVVVPTTYFRWKTEEASRAGASLVIYANQGLRAAVQAVRDVLLEIRLTGDSTSSEDVIAPVKEIFRLTDVDTWNALES	285	EDKVFPKKNS	unknown
Streptomyces phyllanthi	DSM 117319, TISTR 2346	GCF_009377205.1	NZ_OX419519.1 supercluster	45	MTVEGPGTPVPRGPAPSQPADRLRELLTSDSLGFLMEAHSGLSAKIVQDEGFDAIWASGLSIATAFGVRDSNELSWTQVVDVVGSITEVVSTPVLMDGDTGFGNFNNARRLVTSLCRMGVAGVCIEDKVFPKLNSFVGERHPLAEIDEFCGKIKACKDAQTVPAFTVVARVEALVAGHGFGEALRRAEAYREAGADAVFIHSKKTDGKEILRFAQEWAERCPLIVTPTTYHTVPTDAFQEHGVSAVIWANHMMRASIAAMRSACRE	314	EDKVFPKLNS	unknown

Strain	strain designations	Genome accession	P-BGC accession	total no. BGCs	PepM sequence	PepM aa	PepM consensus motif (EDKX^{5}NS)	phosphonate product
					VRQTRTVTGVERRIAPLKEVFDLLDYPELERASHRYLPQRMPLGEEVW			
Streptomyces rubellomurinus	ATCC 31215	GCF_000961885.1	NZ_JZKH01000051.1 Region on contig edge. supercluster	59	MSIAAGVHDGLSARIAQDAGFDVLWASGLGISAAHAVPDDSILTMTEFLEAARVMNDSTDLPVLADCDTGFGDERNVARMVERYERAGIAGVCIEDKVFPKRNSLGDGAQDQETIEGFAAKLAAAKKAQQTDDFVVVARIETFIAGGVKEDAIARADAYVAAGADAILIHSKSRDGLEILDFGRSWQRKDVPLVAVPTTYPAVTADDLHEAGFSLAIYANQALRASITSMRESLRQITHDRSSLRIEPKVAKLSEVFELQR	261	EDKVFPKRNS	FR900098[28,29]
Streptomyces seoulensis	DSM 41840, KCTC 9819	GCF_004328625.1	NZ_CP032229.1 supercluster	23	MSAATTEASPVSARTAADRLRDLFARPGVVRIAGAHNPLGARLAERAGFDGVWSSGLEVSASQGVPDTDILTMSELLGVASSMAAAVDIPVVADCDAGYGNAHNVMNMIRRYEAAGIAAVSIEDKRFPKVNSFIPGRQELAPIGEFCGKLAAAKEAQRHPGLMVIARIEALIAGWGMDEALRRGEAYARAGADAVLIHAKGASPEPILQFLREWRLPTPVVVVPTTYHTITATELGEAGAKLVIYANHGLRAGITAVSDTFQAILRDDRTTGVEDRIAPLATVFDLQGMAAQKRHEAEFITPFESRARAAVVPAAEEPSGRAADLLDHQTAALRRAGIESVSLVTSGEVPAQLPQDTAVLAGHTDAAGAVLELPATQLSATLLLSSDAYVQSEPLARLLAAGGDVTLLVDVSARGKHRADAVPLRLDSRVHEGRRLSAGDGIRVTGVGGQGADGEFAGAAVFSPQGFAALREAAGKRRANGSPATLADLLTDLVEGGHQVGAVEIGSGWMELRTAADAEHAAGLLTGQGEVR	532	EDKRFPKVNS	unknown
Streptomyces sp.	NRRL F-525	GCF_000716605.1	NZ_JNXE01000019.1	43	MSPVKSLRALLHDADLAKAAGAHNPLTAQLVEQAGFDVVWASGLEIAASLGVPDANILSMNECLAVARAMVEKVSVPVLADCDSGFGGIGNVVHMVRSYEAAGVQGVCIEDKTFPKLNSFVAGNQALVPIEDFAGKIAAAVATRVSEDFVIIARIEAFISGYGLDEALRRAEAYELAGADALLIHSKRTTPDEIYSFCAAYDGQLPIVVVPTTYNSVTMEELRAGGASLAIYANQGLRGAITSIRQIFGSILAEGTAAPVENQLAPLGDVFGYQDVAALLSAEDRFESIGQGVAKQILNGAREERRNADVVER	313	EDKTFPKLNS	*O*-phosphonoacetic acid serine[3]
Thermomonospora echinospora	DSM 43163	GCF_900108175.1	NZ_FNVO01000023.1	27	MPMSLPEIRALPALLRPPRNRPVLVAGAHDGMTARLAQRNGFGAVWASGFSLATSHAVPDASVLSMSESLAAVRMMRAATDLPIIADCDTGFEDTADITSLVQSYEAAGVSAVCIEDKAFPKYNSFLSAPQTLETTSVFSAKIEAAKKAQRGTGFMVAARTEAFIAGLGARQAIARAEAYVNAGADMILVHSKASSPHEIYEFMELWDHRSPVAVIPTSYHGVTIDELGSAGIAMVIYAHHAMRAAARAVNMALRSIIDAGTTTHIEKDLATLQEVFEIQGATGLRGSRSAGGEAGSRTRNASKNFISPGDGTGRDTV	318	EDKAFPKYNS	unknown
Umezawaea tangerina	DSM 44720	GCF_003002815.1	NZ_PVTF01000007.1 supercluster	43	MLRREHQSFRTVLAANKVVRLAGAHDAMGAALAEQAGFQGVWASSLEMSAARCLPDASVMTMTEYLEGASNMQKALSIPVVADCDTGYGNNLNVAHMVHEYEAAGITAVCMEDKLFPKMNSFAGGAQTLLSTEAFASKVETAKNAQRDPDFFVIARTEALISGLSVEEALERCHAYADAGADAVLIHSKAKVNDQVLEFLRAWNGHRPVVVVPTTYPEWHVDDAAAAGVSVVIYANQGLRATVSALRDTFKTILTLGETTSLESRIASVTEVFELQNLDDWQKLEV	286	EDKLFPKMNS	unknown
Total no. of strains: 72 (with 74 BGCs / 74 *pepM* genes with EDKX$_5$NS consensus motif)		∅ No. of PBGCs: **36.3**			∅ No. of PepM aa: **316**		DSMZ/Tue strains: 54<br>No. of additionally included non-DSMZ/Tue strains: 18	

Strain	strain designations	Genome accession	P-BGC accession	total no. BGCs	PepM sequence	PepM aa	PepM consensus motif (EDKX^{5}NS)	phosphonate product
							And unknown: 2 (*S. silvensis* and *S.* sp. 31A4)	
45 not previously reported Tue/DSM strains identified as phosphonate producers (**17 BGCs from 16 strains in singletons + 8 BGCs from 8 strains in GCFs with no known producers** (á 5 GCFs, since GCF09 + GCF15 contain non-TUE/DSM strains, that are not known producers) + 21 BGCs from 21 strains in GCFs with known producers)<br>25 known PNP producers (16 non DSMZ/TUE strains (17 BGCs due to *P. ananatis* harbouring 2 BGCs) + 9 DSM/Tue strains)<br>2 non DSMZ/TUE unknown producer strains, included for close homology with strains of interest (*S. silvensis* and *S.* sp. 31A4)								

Table S2. Bioassay results in the test of putative phosphonate producers against *Escherichia coli* WM6242, *E. coli* K12 and *Kocuria rhizophila.*

Inhibition zones were documented by measurement of diameter including the 8 mm diameter agar-block of grown actinomycetes when inhibition of test strains was observed. Values are in [mm]. Strain did not grow on the selected cultivation media (N/A) or was not measured (N/A). Plus (+) indicates presence of putative phosphonate peaks in ^{31}P NMR, minus (-) absence under any tested condition. Strain names and designations highlighted in bold lettering do not belong to any GCF that includes sequences from known producers at a cutoff value of 0.612 in the BiG-SCAPE analysis.

Strain	DSMZ	other strain desigations	^{31}P NMR measured	Inhibition zones in the test against *E. coli* WM 6242										Inhibition zones in the test against *E. coli* K12										Inhibition zones in the test against *Kocuria rhizophila*									
			Media	HM	OM	R5	SFM	NL200	NL300	NL400	NL410	NL500	NL800	HM	OM	R5	SFM	NL200	NL300	NL400	NL410	NL500	NL800	HM	OM	R5	SFM	NL200	NL300	NL400	NL410	NL500	NL800
Actinoalloteichus cyanogriseus	**43889**		**N/A**	0	0	0	0	N/A	N/A	0	0	0	0	0	0	0	0	N/A	N/A	0	0	0	0	20	26	26	26	N/A	N/A	26	26	26	26
Actinokineospora auranticolor	**44650**		**N/A**	0	0	0	0	0	0	0	0	0	0	0	0	0	0	0	0	0	0	0	0	0	12	14	16	15	11	12	13	16	0
Actinopolyspora mzabensis	**45460**		**N/A**	N/A	N/A	N/A	N/A	N/A	N/A	N/A	N/A	N/A	N/A	N/A	N/A	N/A	N/A	N/A	N/A	N/A	N/A	N/A	N/A	N/A	N/A	N/A	N/A	N/A	N/A	N/A	N/A	N/A	N/A
Amycolatopsis xylanica	**45285**		**N/A**	0	0	N/A	0	0	0	0	0	0	0	0	0	N/A	0	0	0	0	0	0	0	0	15	12,5	N/A	0	0	9	12	17	0
Goodfellowiella coeruleoviolacea	**43935**		**N/A**	0	0	N/A	13	0	0	15	13	0	13	0	0	N/A	13,5	0	10	14	12	0	11	10	14	23	N/A	0	18	23	18	0	18
Kibdelosporangium banguiense	**46670**		**+**	0	N/A	N/A	0	0	0	9	0	0	0	0	0	N/A	10	0	0	9	0	0	0	9	9,5	13	N/A	18	N/A	18	15	10	11
Kitasatospora cheerisanensis	**101999**	**KCTC 2395**	**+**	0	0	0	0	0	N/A	0	0	N/A	0	0	0	0	0	0	N/A	0	0	N/A	0	0	0	0	0	0	N/A	0	0	N/A	11
Kitasatospora fiedleri	**114396**	**Tü 4103**	**+**	0	N/A	0	0	0	N/A	0	0	N/A	0	0	0	0	0	N/A	N/A	N/A	0	N/A	0	0	0	9	0	0	N/A	0	10	N/A	9
Saccharopolyspora spinosa	**44228**	**NRRL 18395**	**+**	0	0	0	0	0	0	0	0	0	0	0	0	0	0	0	0	0	0	0	0	0	0	0	0	0	0	0	0	0	0
Streptomyces glauciniger	**41867**	**CGMCC 4.1858**	**-**	0	0	16	0	0	N/A	0	0	N/A	0	0	0	10	0	0	N/A	0	0	N/A	0	9,5	0	13	0	0	N/A	12	12	N/A	0
Streptomyces iranensis	**41954**		**+**	9,5	N/A	N/A	0	0	N/A	N/A	12	N/A	N/A	11	N/A	N/A	0	0	N/A	N/A	13,5	N/A	N/A	11	N/A	N/A	10	0	N/A	13	N/A	N/A	11,5
Streptomyces mutomycini	**41691**	**NRRL B-65393**	**+**	0	0	0	0	0	0	0	0	0	0	0	0	0	0	0	0	0	0	0	0	0	10	9,5	0	0	9	8,5	9	13	8,5
Streptomyces rimosus	**40260**	**ATCC 10970**	**N/A**	15	11	N/A	9	11	N/A	10	10	N/A	14	10,5	13,5	0	10,5	12	N/A	14	10,5	N/A	14	10	11	0	13	9	N/A	12	10,5	N/A	16
Streptomyces seoulensis	**41840**	**KCTC 9819**	**+**	0	0	0	0	0	N/A	0	0	N/A	0	0	0	0	0	0	N/A	0	0	N/A	0	0	0	0	0	0	N/A	0	0	N/A	0
Thermomonospora echinospora	**43163**		**N/A**	N/A	0	N/A	0	N/A	N/A	N/A	0	0	0	N/A	0	N/A	0	N/A	N/A	N/A	0	0	0	11	0	13	0	0	0	11	11	0	13
Umezawaea tangerina	**44720**		**N/A**	0	0	0	0	0	0	0	0	0	0	0	0	N/A	0	0	0	0	0	0	0	0	0	18	N/A	0	0	0	0	0	0
Kitasatospora atroaurantiaca	41649		+	0	0	0	0	9,5	N/A	0	10	N/A	0	0	0	0	0	0	N/A	0	0	N/A	0	9	0	0	0	9	N/A	10	0	N/A	9
Kitasatospora phosalacinea	43860	NRRL B-16230	N/A	0	0	18	0	14	N/A	0	0	N/A	0	0	0	0	0	0	N/A	0	0	N/A	0	0	0	0	0	0	N/A	0	0	N/A	0
Kitasatospora purpeofusca	40283	NRRL B-1817	+	0	0	0	10	0	N/A	11	0	N/A	0	0	0	0	0	0	N/A	0	0	N/A	0	9	0	0	9	0	N/A	10,5	0	N/A	10
Kitasatospora setae	43861	KM-6054	+	0	0	0	0	11	N/A	0	0	N/A	0	0	0	0	0	0	N/A	0	0	N/A	0	0	0	10	0	9	N/A	0	0	N/A	10
Salinispora pacifica	45543	CNS-863	N/A	0	0	N/A	N/A	N/A	N/A	N/A	N/A	N/A	N/A	0	N/A	N/A	N/A	N/A	N/A	N/A	N/A	N/A	N/A	10	0	N/A	N/A	N/A	N/A	N/A	N/A	N/A	N/A
Streptomyces alboniger	40043	ATCC 12461	-	0	0	0	0	0	N/A	0	0	N/A	0	0	0	12	0	0	N/A	0	0	N/A	0	0	9,5	10,5	9,5	9	N/A	0	10	N/A	9,5
Streptomyces bikiniensis	40581	ISP-5580	+	0	9	9	9	11	N/A	0	0	N/A	0	9	11	9,5	0	12	N/A	9	0	N/A	0	9	12,5	12,5	10,5	13	N/A	13	10,5	N/A	10
Streptomyces chrestomyceticus	40545	NBRC 13444	N/A	11,5	0	9	9	0	N/A	9	0	N/A	9	9	0	0	0	0	N/A	9	0	N/A	8,5	10	0	9	9,5	0	N/A	10,5	9	N/A	10
Streptomyces fradiae	40943		N/A	14	9,5	0	9	13	N/A	9	10,5	N/A	10,5	0	0	0	0	0	N/A	9	0	N/A	0	0	0	0	0	0	N/A	9	11	N/A	0
Streptomyces hokutonensis	102214	R1-NS-10	-	0	0	0	9,5	0	11,5	0	0	0	0	0	0	0	0	0	0	0	0	0	0	0	8,5	0	10,5	0	0	0	0	0	0
Streptomyces monomycini	41801	NRRL B-24309	+	0	12	N/A	12	15	13,5	0	13	13	9	0	9,5	0	10	0	0	0	8,5	0	0	10	9	10	10	10,5	9	10	15	11	9
Streptomyces regensis	40551	NRRL B-11479	N/A	0	0	11	0	0	N/A	0	0	N/A	0	0	0	9	0	0	N/A	0	0	N/A	0	15,5	14,5	18	18	14,5	N/A	18	15	N/A	15,5
Streptomyces resistomycificus	40133		+	0	0	0	0	0	N/A	0	0	N/A	0	0	0	0	0	0	N/A	0	0	N/A	0	9	0	9,5	0	0	N/A	0	0	N/A	10
Streptomyces sp.		A4-2	N/A	0	0	0	0	0	0	0	0	0	0	0	0	0	0	0	0	0	0	N/A	N/A	0	0	0	0	0	0	0	0	0	0
Streptomyces sp.		I6	+	0	N/A	10	13	N/A	N/A	N/A	13,5	N/A	0	0	N/A	0	13	N/A	N/A	N/A	0	N/A	0	10,5	N/A	0	18	N/A	N/A	N/A	19	N/A	0
Streptomyces sp.		TUE 18	+	0	9,5	0	0	0	0	0	0	0	0	0	0	0	0	0	0	0	0	0	0	10,5	9	0	8,5	0	10,5	10	10,5	9	12
Streptomyces sp.		TUE 21470	+	0	0	0	0	0	0	0	0	0	0	0	0	0	0	0	0	0	0	0	0	10	10	10	0	9	0	10,5	13	10	13
Streptomyces sp.		TUE 3678	+	0	0	N/A	0	0	0	0	0	N/A	N/A	0	0	N/A	0	0	0	0	0	N/A	N/A	8,5	0	0	N/A	0	0	8,5	0	N/A	N/A
Streptomyces sp.		TUE 3997	+	0	0	0	0	0	0	0	0	0	0	0	0	0	0	0	0	0	0	0	0	12	13	12	11	0	11	9	10	8,5	14
Streptomyces viridochromogenes	40736	TUE 494	+	0	0	14	0	12	N/A	9	0	N/A	0	0	0	14	0	0	N/A	0	0	N/A	0	0	0	0	0	0	N/A	0	0	N/A	0
Total tested		36																															

Table S3. Plasmids used in this study

Name	Description	Source
pIJ10257	*Streptomyces* expression vector; *ermEp**, *hygB*, phiBT1-*int*, phiBT1-*attP*, RK2-*oriT*, ColE1-*ori*	JIC StrepStrains; Hong et al., 2005[30]
pRM4	*Streptomyces* expression vector; *ermEp**, *aac(3)IV*, phiC31-*int*, phiC31-*attP*, RK2-*oriT*, ColE1-*ori*	University of Tübingen; Menges et al., 2007[31]
pGus21	Knock out vector, non-replicative in *Streptomyces*; *aac(3)IV*, RK2-*oriT*, *ermEp**::*gusA*, pMB1- *ori*, *I-SceI-site*	University of Tübingen; Ladwig et al., 2015[32]
pTC192-km	Source of kanamycin resistance gene *neo* ColE1-*ori*, *bla*, *neo*	University of Leon; Rodríguez-García et al., 2006[33]
pBluescript II KS(+)	General *E. coli* cloning vector; ColE1-*ori*, *bla*	Alting-Mees and Short, 1989[34]
pDS0101	pIJ10257 derivative, *ermEp*::kfp25*; *luxR* overexpression	This work
pDS0102	pRM4 derivative, *ermEp*::kfp25*; *luxR* overexpression	This work
pDS0105	pRM4 derivative, *ermEp*::kfp02-kfp04*; *pepMppdAB* overexpression	This work
pDS0106	pIJ10257 derivative, *ermEp*::kfp02-kfp04*; *pepMppdAB* overexpression	This work
pDS0107	pGus21 derivative, gene replacement of *kfp02-kfp04* (*pepMppdAB*) with *neo*	This work

Table S4. Strains used in this study

Name	Description	Source
Escherichia coli ET12567/pUZ8002	Donor strain for conjugation of plasmids to *Kitasatospora* sp.	MacNeil et al. 1992, Paget et al. 1999[35,36]
Escherichia coli DH5a DSM 6897	General cloning strain	DSM[37,38]
Escherichia coli WM6242	Phosphonate antimicrobial indicator microorganism	University of Illinois[29]
Kocuria. rhizophila DSM 348	Antimicrobial indicator microorganism	DSM
Streptomyces albus J1074	Non-phosphonate producer used as heterologous host for *pepMppdAB* overexpression	Chater and Wilde 1976, Chater and Carter 1979[39,40]
Streptomyces lividans T7	Non-phosphonate producer used as heterologous host for *pepMppdAB* overexpression	Lussier et al. 2010[41]
Kitasatospora fiedleri DSM 114396	University of Tübingen collection Tü4103; type strain	Zimmermann et al.[1]
Kitasatospora fiedleri YM0107	*K. fiedleri* DSM 114396 phiBT1*attB*::pDS0101 (*luxR* overexpression)	This work
Kitasatospora fiedleri YM0108	*K. fiedleri* DSM 114396 phiC31 *attB*::pDS0102 (*luxR* overexpression)	This work

Kitasatospora fiedleri YM0128	*K. fiedleri* DSM 114396 phiC31 *attB*::pDS0105 (*pepMppdAB* overexpression)	This work
Kitasatospora fiedleri YM0129	*K. fiedleri* DSM 114396 phiBT1*attB*::pDS0106 (*pepMppdAB* overexpression)	This work
Kitasatospora fiedleri YM0167	*K. fiedleri* YM0107 phiC31 *attB*::pDS0105 (*luxR pepMppdAB* overexpression)	This work
Kitasatospora fiedleri YM0168	*K. fiedleri* YM0108 phiBT1*attB*::pDS0106 (*luxR pepMppdAB* overexpression)	This work
Kitasatospora fiedleri YM0173	*K. fiedleri* DSM 114396 Δ*kfp02-04::neo* (*pepM-ppdAB* null mutant)	This work
Kitasatospora fiedleri YM0178	*K. fiedleri* YM0173 phiBT1*attB*::pDS0106 (complementation of *pepM-ppdAB* null mutant)	This work
Streptomyces albus YM0158	*Streptomyces albus* J1074 phiC31 *attB*::pDS0105 (*pepMppdAB* overexpression)	This work
Streptomyces albus YM0159	*Streptomyces albus* J1074 phiBT1*attB*::pDS0106 (*pepMppdAB* overexpression)	This work
Streptomyces lividans YM0152	*Streptomyces lividans* T7 phiC31 *attB*::pDS0105 (*pepMppdAB* overexpression)	This work
Streptomyces lividans YM0153	*Streptomyces lividans* T7 phiBT1*attB*::pDS0106 (*pepMppdAB* overexpression)	This work

Table S5. Oligonucleotides used in this study

**Name**	**Sequence (5'→ 3')**	**Description**
M13F	CGCCAGGGTTTTCCCAGTCACGAC	Universal primer for sequencing of insert in many *lacZ*-containing vectors
M13R	TCACACAGGAAACAGCTATGAC	Universal primer for sequencing of insert in many *lacZ*-containing vectors
JP503	GCGATCACCGACAAGCTC	PCR test for *pepM-ppdAB* mutant screening
JP508	GGAAGTTCCACGGGGTGAT	PCR test for *pepM-ppdAB* mutant screening
JP525	TTCATATGCGTGCATTGGGCGA	Cloning of *kfp24* (putative LuxR-like regulator)
JP526	AAGCTTGAACGCCGAACCGGATATGT	Cloning of *kfp24* (putative LuxR-like regulator)
JP549	TTCATATGCACACGTCCAGCGTG	Cloning of *ppdB*, *ppdA*, and *pepM* (*kfp02*, *kfp03*, and *kfp04*) with NdeI site
JP550	AAAAGCTTGCTCCATCGTGATCCCTTC	Cloning of *ppdB*, *ppdA*, and *pepM* (*kfp02*, *kfp03*, and *kfp04*); HindIII underlined
JP551	TTGGATCCTGGCGCTGGTGCTGTTCAAG	Cloning of homologous region downstream of *kfp02-04* (*pepM-ppdAB);* BamHI underlined
JP552	AAACTAGTCCACGAAGGGATCACGATGG	Cloning of homologous region downstream of *kfp02-04* (*pepM-ppdAB);* SpeI underlined
JP553	TTGACCTTCCGTCGGTCGGTTC	Cloning of homologous region upstream of *kfp02-04* (*pepM-ppdAB)*
JP554	TTCTCGAGAGGTCGGCAAGACCACCTTC	Cloning of homologous region upstream of *kfp02-04* (*pepM-ppdAB*); XhoI underlined

JP555	GCTCCATCGTGATCCCTTC	Cloning of *ppdB*, *ppdA*, and *pepM* (*kfp02*, *kfp03*, and *kfp04*)/ PCR test for *pepM-ppdAB* mutant screening
JP556	GCTGGTGTTCTGCAAGAC	Cloning of *ppdB*, *ppdA*, and *pepM* (*kfp02*, *kfp03*, and *kfp04*)/ PCR test for *pepM-ppdAB* mutant screening
JP569	CCCTGGTTGGCGTAGATCAC	PCR test for *pepM-ppdAB* mutant screening
JP570	GAACGGTTCCGAGGTCATCC	PCR test for *pepM-ppdAB* mutant screening
JP571	CTCGACGAAGCTGTTGAG	PCR test for *pepM-ppdAB* mutant screening
JP572	ATGTCCAAGAGCCGTACC	PCR test for *pepM-ppdAB* mutant screening
JP573	ATGCCGTTGTCGACCACCAC	PCR test for *pepM-ppdAB* mutant screening
JP574	GTGGTCCTGATGCAGAACTC	PCR test for *pepM-ppdAB* mutant screening
JP575	TCTCGTGCATGCCGTTGTC	PCR test for *pepM-ppdAB* mutant screening
JP576	CGTGGTCCTGATGCAGAACTC	PCR test for *pepM-ppdAB* mutant screening
JP581	GGCTACCCGTGATATTGC	PCR test for *pepM-ppdAB* mutant screening
JP582	GCGGTGATGATGATCGAG	PCR test for *pepM-ppdAB* mutant screening
JP583	CCTCGTCCTGCAGTTCATTC	PCR test for *pepM-ppdAB* mutant screening
JP584	ACTCCACCACGTCGATCTAC	PCR test for *pepM-ppdAB* mutant screening
pepMupfw	ATGAATTCTTCCCGGAGCTGGTGTTCTG	PCR test for *pepM-ppdAB* mutant screening
pepMuprv	ATTCTAGAGGATGACCTCGGAACCGTTC	PCR test for *pepM-ppdAB* mutant screening
RTpepM2fw	CTGAACGGTTCCGAGGTC	PCR test for *pepM-ppdAB* mutant screening
RTpepM2rv	TGGTACGAGCGGACCATGTG	PCR test for *pepM-ppdAB* mutant screening

***Table S6.* Proposed phosphonate biosynthetic gene cluster from Kitasatospora fiedleri DSM 114396, based on the chromosome sequence with NCBI accession NZ_OX419519.1**

Gene	Locus Tag	From position	To position	Strand	Size (bp)	Size (aa)	Genbank annotated product [new assigned gene and function]	Match
kfp01	QMQ26_RS15690	3401998	3403470	c	1473	490	aldehyde dehydrogenase [*adh* gene, phosphonoacetaldehyde dehydrogenase]	Adh
kfp02	QMQ26_RS15695	3403515	3404408	c	894	297	isocitrate lyase/phosphoenolpyruvate mutase family protein [*pepM* gene, PEP mutase]	PepM
kfp03	QMQ26_RS15700	3404459	3405058	c	600	199	thiamine pyrophosphate-dependent enzyme [*ppdA* gene, phosphonopyruvate decarboxylase subunit A]	PpdA
kfp04	QMQ26_RS15705	3405058	3405582	c	525	174	thiamine pyrophosphate-binding protein [*ppdB* gene, phosphonopyruvate decarboxylase subunit A]	PpdB
kfp05	QMQ26_RS15710	3405626	3406912	c	1287	428	acetyl-CoA carboxylase biotin carboxylase subunit family protein	
kfp06	QMQ26_RS15715	3406909	3407958	c	1050	349	hypothetical protein	
kfp07	QMQ26_RS15720	3408011	3408868	c	858	285	TauD/TfdA family dioxygenase	
kfp08	QMQ26_RS15725	3408894	3409289	c	396	131	hypothetical protein	
kfp09	QMQ26_RS15730	3409286	3410608	c	1323	440	cation:proton antiporter	
kfp10	QMQ26_RS15735	3410601	3411887	c	1287	428	sulfate adenylyltransferase subunit 1	CysN
kfp11	QMQ26_RS15740	3411890	3412810	c	921	306	sulfate adenylyltransferase subunit CysD	CysD
kfp12	QMQ26_RS15745	3412807	3413361	c	555	184	adenylyl-sulfate kinase CysC	CysC
kfp13	QMQ26_RS15750	3413358	3415343	c	1986	661	inositol monophosphatase family protein	CysQ
kfp14	QMQ26_RS15755	3415309	3416571	c	1263	420	MFS transporter	
kfp15	QMQ26_RS15760	3416682	3417728	c	1047	348	aspartate carbamoyltransferase PyrB	
kfp16	QMQ26_RS15765	3417845	3418054	c	210	69	hypothetical protein	
kfp17	QMQ26_RS15770	3418097	3418969	c	873	290	TauD/TfdA family dioxygenase	
kfp18	QMQ26_RS15775	3419045	3420304	c	1260	419	hypothetical protein	
kfp19	QMQ26_RS15780	3420279	3422249	c	1971	656	S9 family peptidase	
kfp20	QMQ26_RS15785	3422246	3423256	c	1011	336	histidinol-phosphate transaminase	
kfp21	QMQ26_RS15790	3423272	3424531	c	1269	422	hypothetical protein	
kfp22	QMQ26_RS15795	3424717	3425499	c	783	260	hypothetical protein	
kfp23	QMQ26_RS15800	3425565	3425738	c	174	57	hypothetical protein	
kfp24	QMQ26_RS15805	3426408	3427439	c	1032	343	LuxR C-terminal-related transcriptional regulator	
kfp25	QMQ26_RS15810	3427581	3428393	c	813	271	NUDIX domain-containing protein	

References

1. Zimmermann A, Nouioui I, Pötter G, Neumann-Schaal M, Wolf J, Wibberg D, et al. *Kitasatospora fiedleri* sp. nov., a novel antibiotic-producing member of the genus *Kitasatospora.* International Journal of Systematic and Evolutionary Microbiology. 2023;73(11):006137.

2. Bibb MJ, Findlay PR, Johnson MW. The relationship between base composition and codon usage in bacterial genes and its use for the simple and reliable identification of protein-coding sequences. Gene. 1984 Oct 1;30(1):157–66.

3. Freestone TS, Ju KS, Wang B, Zhao H. Discovery of a Phosphonoacetic Acid Derived Natural Product by Pathway Refactoring. ACS Synth Biol. 2017 Feb 17;6(2):217–23.

4. Agarwal V, Peck SC, Chen JH, Borisova SA, Chekan JR, van der Donk WA, et al. Structure and function of phosphonoacetaldehyde dehydrogenase: the missing link in phosphonoacetate formation. Chem Biol. 2014 Jan 16;21(1):125–35.

5. Cioni JP, Doroghazi JR, Ju KS, Yu X, Evans BS, Lee J, et al. Cyanohydrin Phosphonate Natural Product from *Streptomyces regensis.* J Nat Prod. 2014 Feb 28;77(2):243–9.

6. Ju KS, Gao J, Doroghazi JR, Wang KKA, Thibodeaux CJ, Li S, et al. Discovery of phosphonic acid natural products by mining the genomes of 10,000 actinomycetes. Proceedings of the National Academy of Sciences. 2015 Sept 29;112(39):12175–80.

7. Takahashi E, Kimura T, Nakamura K, Arahira M, Iida M. Phosphonothrixin, a Novel Herbicidal Antibiotic Produced by *Saccharothrix* sp. ST-888 I. Taxonomy, Fermentation, Isolation and Biological Properties. J Antibiot. 1995 Oct 25;48(10):1124–9.

8. Bown L, Hirota R, Goettge MN, Cui J, Krist DT, Zhu L, et al. A Novel Pathway for Biosynthesis of the Herbicidal Phosphonate Natural Product Phosphonothrixin Is Widespread in Actinobacteria. Journal of Bacteriology. 2023 Apr 19;0(0):e00485-22.

9. Evans BS, Zhao C, Gao J, Evans CM, Ju KS, Doroghazi JR, et al. Discovery of the Antibiotic Phosacetamycin via a New Mass Spectrometry-Based Method for Phosphonic Acid Detection. ACS Chem Biol. 2013 May 17;8(5):908–13.

10. Nouioui I, Zimmermann A, Hennrich O, Xia S, Rössler O, Makitrynskyy R, et al. Challenging old microbiological treasures for natural compound biosynthesis capacity. Front Bioeng Biotechnol. 2024;12:1255151.

11. Kayrouz CM, Zhang Y, Pham TM, Ju KS. Genome Mining Reveals the Phosphonoalamide Natural Products and a New Route in Phosphonic Acid Biosynthesis. ACS Chem Biol. 2020 July 17;15(7):1921–9.

12. Circello BT, Eliot AC, Lee JH, Donk WA van der, Metcalf WW. Molecular Cloning and Heterologous Expression of the Dehydrophos Biosynthetic Gene Cluster. Chemistry & Biology. 2010 Apr 23;17(4):402–11.

13. Schwartz D, Recktenwald J, Pelzer S, Wohlleben W. Isolation and characterization of the PEP-phosphomutase and the phosphonopyruvate decarboxylase genes from the phosphinothricin

tripeptide producer *Streptomyces viridochromogenes* Tü494. FEMS Microbiology Letters. 1998 June 15;163(2):149–57.

14. Grammel N, Schwartz D, Wohlleben W, Keller U. Phosphinothricin-Tripeptide Synthetases from *Streptomyces viridochromogenes.* Biochemistry. 1998 Feb 1;37(6):1596–603.

15. Hidaka T, Shimotohno KW, Morishita T, Seto H. Studies on the biosynthesis of bialaphos (SF-1293). 18. 2-phosphinomethylmalic acid synthase: a descendant of (R)-citrate synthase? J Antibiot (Tokyo). 1999 Oct;52(10):925–31.

16. Polidore ALA, Caserio AD, Zhu L, Metcalf WW. Complete Biochemical Characterization of Pantaphos Biosynthesis Highlights an Unusual Role for a SAM-Dependent Methyltransferase. Angewandte Chemie International Edition. 2023;n/a(n/a):e202317262.

17. Woodyer RD, Shao Z, Thomas PM, Kelleher NL, Blodgett JAV, Metcalf WW, van der Donk WA, Zhao H. Heterologous Production of Fosfomycin and Identification of the Minimal Biosynthetic Gene Cluster. 2006. Chemistry & Biology 13, 1171–1182.

18. Hidaka T, Goda M, Kuzuyama T, Takei N, Hidaka M, Seto H. Cloning and nucleotide sequence of fosfomycin biosynthetic genes of *Streptomyces wedmorensis.* Molec Gen Genet. 1995 May 1;249(3):274–80.

19. Kobayashi S, Kuzuyama T, Seto H. Characterization of the *fomA* and *fomB* Gene Products from *Streptomyces wedmorensis*, Which Confer Fosfomycin Resistance on *Escherichia coli*. Antimicrob Agents Chemother. 2000 Mar;44(3):647–50.

20. Borisova SA, Circello BT, Zhang JK, van der Donk WA, Metcalf WW. Biosynthesis of Rhizocticins, Antifungal Phosphonate Oligopeptides Produced by *Bacillus subtilis* ATCC6633. Chemistry & Biology. 2010 Jan 29;17(1):28–37.

21. Cui J, Ju KS. Biosynthesis of *Bacillus* Phosphonoalamides Reveals Highly Specific Amino Acid Ligation. ACS Chem Biol. 2024 July 19;19(7):1506–14.

22. Yu X, Price NPJ, Evans BS, Metcalf WW. Purification and Characterization of Phosphonoglycans from *Glycomyces* sp. Strain NRRL B-16210 and *Stackebrandtia nassauensis* NRRL B-16338. J Bacteriol. 2014 May;196(9):1768–79.

23. Omura S, Murata M, Hanaki H, Hinotozawa K, Oiwa R, Tanaka H. Phosalacine, a new herbicidal antibiotic containing phosphinothricin. Fermentation, isolation, biological activity and mechanism of action. J Antibiot (Tokyo). 1984 Aug;37(8):829–35.

24. Blodgett JA, Zhang JK, Yu X, Metcalf WW. Conserved biosynthetic pathways for phosalacine, bialaphos and newly discovered phosphonic acid natural products. J Antibiot. 2016 Jan;69(1):15–25.

25. Shoji J, Kato T, Hinoo H, Hattori T, Hirooka K, Matsumoto K, et al. Production of fosfomycin (phosphonomycin) by *Pseudomonas syringae.* J Antibiot (Tokyo). 1986 July;39(7):1011–2.

26. Zhang Y, Chen L, Wilson JA, Cui J, Roodhouse H, Kayrouz C, et al. Valinophos Reveals a New Route in Microbial Phosphonate Biosynthesis That Is Broadly Conserved in Nature. J Am Chem Soc. 2022 June 8;144(22):9938–48.

27. Parkinson EI, Erb A, Eliot AC, Ju KS, Metcalf WW. Fosmidomycin biosynthesis diverges from related phosphonate natural products. Nat Chem Biol. 2019 Nov;15(11):1049–56.

28. Iguchi E, Okuhara M, Kohsaka M, Aoki H, Imanaka H. Studies on new phosphonic acid antibiotics II. Taxonomic studies on producing organisms of the phosphonic acid and related compounds. J Antibiot. 1980;33(1):18–23.

29. Eliot AC, Griffin BM, Thomas PM, Johannes TW, Kelleher NL, Zhao H, et al. Cloning, Expression, and Biochemical Characterization of *Streptomyces rubellomurinus* Genes Required for Biosynthesis of Antimalarial Compound FR900098. Chemistry & Biology. 2008 Aug 25;15(8):765–70.

30. Hong HJ, Hutchings MI, Hill LM, Buttner MJ. The role of the novel Fem protein VanK in vancomycin resistance in *Streptomyces coelicolor.* J Biol Chem. 2005 Apr 1;280(13):13055–61.

31. Menges R, Muth G, Wohlleben W, Stegmann E. The ABC transporter Tba of *Amycolatopsis balhimycina* is required for efficient export of the glycopeptide antibiotic balhimycin. Appl Microbiol Biotechnol. 2007 Nov 1;77(1):125–34.

32. Ladwig N, Franz-Wachtel M, Hezel F, Soufi B, Macek B, Wohlleben W, et al. Control of Morphological Differentiation of *Streptomyces coelicolor* A3(2) by Phosphorylation of MreC and PBP2. PLOS ONE. 2015 Apr 30;10(4):e0125425.

33. Rodríguez-García A, Santamarta I, Pérez-Redondo R, Martín JF, Liras P. Characterization of a two-gene operon *epeRA* involved in multidrug resistance in *Streptomyces clavuligerus.* Res Microbiol. 2006 Aug;157(6):559–68.

34. Alting-Mees MA, Short JM. pBluescript II: gene mapping vectors. Nucleic Acids Res. 1989 Nov 25;17(22):9494–9494.

35. MacNeil DJ, Gewain KM, Ruby CL, Dezeny G, Gibbons PH, MacNeil T. Analysis of *Streptomyces avermitilis* genes required for avermectin biosynthesis utilizing a novel integration vector. Gene. 1992 Feb 1;111(1):61–8.

36. Paget MSB, Chamberlin L, Atrih A, Foster SJ, Buttner MJ. Evidence that the Extracytoplasmic Function Sigma Factor ςE Is Required for Normal Cell Wall Structure in *Streptomyces coelicolor* A3(2). J Bacteriol. 1999 Jan 1;181(1):204–11.

37. Bethesda Research Laboratories. BRL pUC Host: *E. Coli* DH5alpha Competent Cells'. 1986;Focus 8(2).

38. Grant SG, Jessee J, Bloom FR, Hanahan D. Differential plasmid rescue from transgenic mouse DNAs into *Escherichia coli* methylation-restriction mutants. Proceedings of the National Academy of Sciences. 1990 June;87(12):4645–9.

39. Chater KF, Wilde LC. Restriction of a bacteriophage of *Streptomyces albus* G involving endonuclease SalI. Journal of Bacteriology. 1976 Nov;128(2):644–50.

40. Chater KF, Carter AT. A New, Wide Host-range, Temperate Bacteriophage (R4) of *Streptomyces* and its Interaction with some Restriction-Modification Systems. Microbiology. 1979;115(2):431–42.

41. Lussier FX, Denis F, Shareck F. Adaptation of the Highly Productive T7 Expression System to *Streptomyces lividans*. Applied and Environmental Microbiology. 2010 Feb;76(3):967–70.
